# LncRNA109897-JrCCR4-JrTLP1b forms a positive feedback loop to regulate walnut resistance against anthracnose caused by *Colletotrichum gloeosporioides*

**DOI:** 10.1093/hr/uhad086

**Published:** 2023-05-03

**Authors:** Rui Zhou, Yuhui Dong, Changxi Wang, Jianning Liu, Qiang Liang, Xiaoye Meng, Xinya Lang, Shengyi Xu, Wenjun Liu, Shuhui Zhang, Nan Wang, Ke Qiang Yang, Hongcheng Fang

**Affiliations:** State Forestry and Grassland Administration Key Laboratory of Silviculture in the Downstream Areas of the Yellow River, Shandong Taishan Forest Ecosystem Research Station, College of Forestry, Shandong Agricultural University, Tai’an, Shandong, China, 271018; State Forestry and Grassland Administration Key Laboratory of Silviculture in the Downstream Areas of the Yellow River, Shandong Taishan Forest Ecosystem Research Station, College of Forestry, Shandong Agricultural University, Tai’an, Shandong, China, 271018; State Forestry and Grassland Administration Key Laboratory of Silviculture in the Downstream Areas of the Yellow River, Shandong Taishan Forest Ecosystem Research Station, College of Forestry, Shandong Agricultural University, Tai’an, Shandong, China, 271018; State Forestry and Grassland Administration Key Laboratory of Silviculture in the Downstream Areas of the Yellow River, Shandong Taishan Forest Ecosystem Research Station, College of Forestry, Shandong Agricultural University, Tai’an, Shandong, China, 271018; State Forestry and Grassland Administration Key Laboratory of Silviculture in the Downstream Areas of the Yellow River, Shandong Taishan Forest Ecosystem Research Station, College of Forestry, Shandong Agricultural University, Tai’an, Shandong, China, 271018; Department of Natural Resources Of Shandong Province, Forestry Protection and Development Service Center, Jinan, Shandong, China， 250000; State Forestry and Grassland Administration Key Laboratory of Silviculture in the Downstream Areas of the Yellow River, Shandong Taishan Forest Ecosystem Research Station, College of Forestry, Shandong Agricultural University, Tai’an, Shandong, China, 271018; State Forestry and Grassland Administration Key Laboratory of Silviculture in the Downstream Areas of the Yellow River, Shandong Taishan Forest Ecosystem Research Station, College of Forestry, Shandong Agricultural University, Tai’an, Shandong, China, 271018; State Key Laboratory of Crop Biology, College of Horticulture Sciences and Engineering, Shandong Agricultural University, Tai’an, Shandong, China， 271018; State Key Laboratory of Crop Biology, College of Horticulture Sciences and Engineering, Shandong Agricultural University, Tai’an, Shandong, China， 271018; State Key Laboratory of Crop Biology, College of Horticulture Sciences and Engineering, Shandong Agricultural University, Tai’an, Shandong, China， 271018; State Forestry and Grassland Administration Key Laboratory of Silviculture in the Downstream Areas of the Yellow River, Shandong Taishan Forest Ecosystem Research Station, College of Forestry, Shandong Agricultural University, Tai’an, Shandong, China, 271018; State Forestry and Grassland Administration Key Laboratory of Silviculture in the Downstream Areas of the Yellow River, Shandong Taishan Forest Ecosystem Research Station, College of Forestry, Shandong Agricultural University, Tai’an, Shandong, China, 271018

## Abstract

Walnut anthracnose induced by *Colletotrichum gloeosporioides* is a disastrous disease that severely restricts the development of the walnut industry in China. Long non-coding RNAs (lncRNAs) are involved in adaptive responses to disease, but their roles in the regulation of walnut anthracnose resistance response are not well defined. In this study, transcriptome analysis demonstrated that a *C. gloeosporioides*-induced lncRNA, lncRNA109897, located upstream from the target gene *JrCCR4*, upregulated the expression of *JrCCR4*. JrCCR4 interacted with JrTLP1b and promoted its transcriptional activity. In turn, JrTLP1b induced the transcription of *lncRNA109897* to promote its expression. Meanwhile, transient expression in walnut leaves and stable transformation of *Arabidopsis thaliana* further proved that lncRNA, JrCCR4, and JrTLP1b improve the resistance of *C. gloeosporioides*. Collectively, these findings provide insights into the mechanism by which the lncRNA109897-JrCCR4-JrTLP1b transcriptional cascade regulates the resistance of walnut to anthracnose.

## Introduction

The identification and characterization of resistance genes (R) encoding pathogen-associated molecular patterns (PAMPs), immune receptors, pathogen Avr effectors, and key signaling components of plant immune responses have resulted in the two-tiered plant innate immune machinery, namely PAMP-triggered immunity (PTI) and effector-triggered immunity (ETI), being established [1–6]. Up till the present moment, there are more than 213 R genes in barley (*Hordeum vulgare* L.), rice (*Oryza sativa* L.), wheat (*Triticum aestivum* L.), maize (*Zea mays* L.), and other plant species, and all of them have been identified as pathogen resistant [7, 8]. Added to that, the common nucleotide-binding leucine-rich repeat proteins (NLRs) and pattern recognition receptors (PRRs), R genes also encode serine–threonine kinase without leucine-rich repeats (LRRs) [9–11]. The Avr protein AvrPto or AvrPtoB was originally identified to interact with the serine–threonine protein kinase Pto, and subsequently, Pto was reported to act as the guardee of a nucleotide-binding and leucine-rich repeat (NB-LRR) protein (Prf) to trigger ETI [12, 13]. The resistance of susceptible tobacco (*Nicotiana tabacum* L.) to *Phytophthora parasitica* var. *nicotianae* increased significantly with the overexpression of serine–threonine protein kinase NrSTK, indicating that *NrSTK* enhances the black shank of tobacco [14].

Meanwhile, pathogenesis-related (PR) proteins act as essential components of the plant innate immune response to play an important role in the defense system [15]. Among the 17 PR protein families, PR5 family proteins named as thaumatin-like proteins (TLPs), which have been studied to confer antifungal activity in plants through inhibiting mycelial growth and spore germination [16, 17]. Overexpression of *TaTLP1* can significantly improve wheat resistance to leaf rust induced by *Puccinia triticina* and common root rot induced by *Bipolaris sorokiniana* [18]. Furthermore, the interaction of TaTLP1-TaPR1 contributes to wheat resistance to leaf rust fungus in a reactive oxygen species (ROS)-dependent manner [19]. In potato (*Solanum tuberosum*), the heterologous expression of CsTLP, isolated from *Camellia sinensis,* conferred resistance to *Phytophthora infestans* and *Macrophomina phaseolina* [20]. GhPR5 acts as a positive regulator in cotton (*Gossypium* spp.) resistance against *Verticillium dahliae*, whose antifungal activity is decreased by the interaction of PevD1–GhPR5 [21]. The PR5 osmotin interacts with BcIEB1 to regulate the elicitor activity of BcIEB1 and protect plants from *Botrytis cinerea* [22]*.*

Walnut (*Juglans regia* L.) is an important nut tree species globally with various health benefits due to its abundance of proteins, unsaturated fatty acids, vitamins, and minerals [23–25]. Walnut anthracnose, induced by *Colletotrichum gloeosporioides* (Penz.) Penz. and Sacc, has become a major factor in the decline of walnut yield, and it leads to leaf defoliation and fruit gangrene [26]. The main measure used to prevent walnut anthracnose is chemical control, which is limited and inefficient due to pathogen resistance and environmental problems [27–29]. Therefore, effectively controlling walnut anthracnose through genomics-based tools and molecular functional research is urgently needed. The former study evaluating the functional genomics and mechanisms of walnut resistance to *C. gloeosporioides* has primarily concentrated on omics analysis and protein-coding genes [30–32]. However, the mechanism by which long non-coding RNAs (lncRNAs) regulate walnut anthracnose resistance is still unclear.

LncRNAs are a class of RNA molecules whose transcript length exceeds 200 nt and lack an encoding function, and these were originally recognized as the transcription ‘noise’ of genes [33, 34]. However, the research in the past ten years has led to a deeper understanding of the *cis-* or *trans-* regulation of gene expression by lncRNA, and they can act as target mimics, or recruit epigenetic complexes to participate in plant biological processes [35–38]. LncRNAs can regulate their target genes through the interaction of DNA, RNA or protein, and control the expression of target genes at the level of transcription, post-transcription and translation [39]. LncRNAs in *cis* mediate neighboring genes at the transcriptional level, while lncRNAs in *trans* regulate gene expression at the transcriptional or posttranscriptional level in diverse biological processes [40]. Meanwhile, increasing evidence has also demonstrated that lncRNAs are of great importance in the plant immune response. *GhlncNAT-ANX2* and *GhlncNAT-RLP7* increase the expression of LOX1 (lipoxygenase 1) and LOX2 (lipoxygenase 2) to enhance seedling resistance toward *V. dahliae* and *B. cinerea* in cotton [41]. Additionally, *GhlncLOX3* can promote resistance to *V. dahliae* by regulating *GhLOX3* expression and jasmonic acid (JA) content [42]. LncRNA33732, activated by WRKY1, enhances tomato (*Solanum lycopersicum*) resistance to *P. infestans* by upregulating the expression of the *RBOH* gene and increasing the accumulation of H_2_O_2_ [43]. The tomato lncRNA16397 also enhances resistance to *P. infestans* through inducing *SlGRX* expression to alleviate cell membrane injury and reduce ROS accumulation [44].

In the current research, we utilized the anthracnose resistant and anthracnose susceptible strains of walnut described in previous studies for lncRNA sequencing using the Illumina HiSeq 4000 platform (https://www.illumina.com) [32]. A total of 645 differentially expressed lncRNAs (DELs) were identified in L423 vs. L37 during five infection stages of *C. gloeosporioides*. LncRNA109897 (MSTRG.109897.16) was identified through sequencing data analysis and was shown to be induced by *C. gloeosporioides* and to regulate walnut resistance to anthracnose by mediating the positive regulation of the target gene *JrCCR4* (serine/threonine-protein kinase-like protein CCR4, LOC108987657) expression. Furthermore, JrCCR4 interacts with JrTLP1b and induces its expression. In turn, JrTLP1b can induce the transcription of *lncRNA109897* to promote its expression. Thus, our findings indicate that the lncRNA109897-JrCCR4-JrTLP1b transcriptional cascade is a positive feedback regulation of walnut resistance to anthracnose and provided insights for the role of lncRNA in the coordinated regulation of walnut resistance to anthracnose.

## Results

### Genome-wide identification of lncRNAs responding to *C. gloeosporioides*

**Figure 1:**
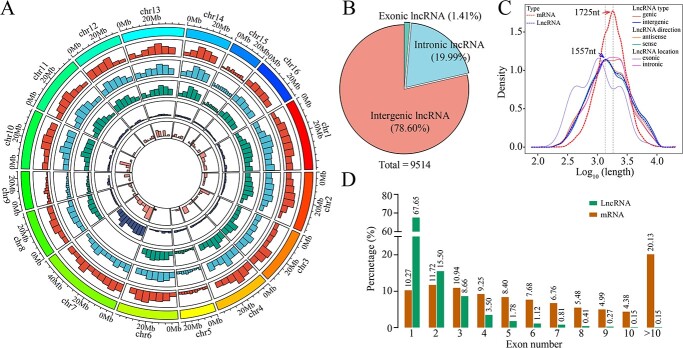
Genome-wide identification of lncRNAs responding to *Colletotrichum gloeosporioides*. **A** Distribution of different types of lncRNAs along each walnut chromosome. From outside to inside: sense, antisense, intergenic, intronic, exonic. **B** Genome location statistics of lncRNAs. **C** Length comparison of lncRNAs and mRNAs. **D** Percentage of lncRNAs and mRNAs containing different numbers of exons.

To explore the lncRNAs involved in the resistance of walnut to anthracnose and identify the lncRNAs responding to *C. gloeosporioides*, our previously published transcriptome database (Bioproject Number: PRJNA776681) was analysed, in which the leaves of resistant (L423) and susceptible strains (L37) at five infection times were used as sequencing samples [32]. After assembly, annotation, and filtering of all transcripts from the RNA-Seq results, approximately 9514 unique lncRNAs [transcript length ≥200 nt, open reading frame (ORF) coverage <50%, and potential coding score <0.5] were obtained using FEELnc. As shown in Fig. 1A, these lncRNAs were more evenly distributed across the 16 chromosomes in walnut. The majority of lncRNAs (78.60%) were located in intergenic regions, and 21.40% of the lncRNAs (intronic: 19.99%, exonic: 1.34%) overlapped with protein-coding genes (Fig. 1B). The lncRNAs range in length from 200 to 12 311 nucleotides (nt), with a median length of 1557 nt that is shorter than the median length (1725 nt) of the mRNAs (Fig. 1C), and the number of exons of the lncRNAs was significantly different from that of the mRNAs (Fig. 1D).

### Functional prediction and target analysis of DELs

**Figure 2:**
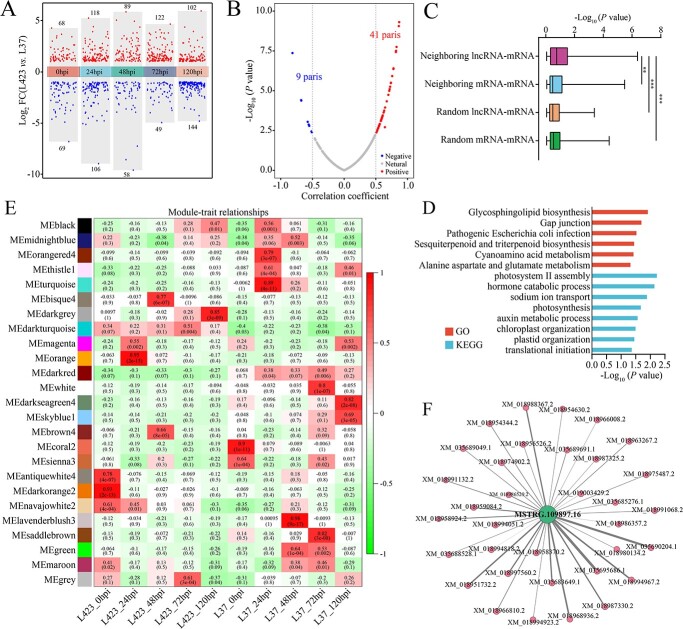
Functional prediction and targets analysis of DELs. **A** Scatter plot of DELs in L423 vs L37 in five infection time. The red and blue dots are upregulated and downregulated, respectively. **B** Scatter plots of Pearson correlation coefficient and *P*-values between the expressions of the lncRNAs and their neighboring protein-coding genes. The x-axis indicates the correlation coefficient and the y-axis indicates the log10 (*P*-values). **C** Comparison of correlation between neighboring and random co-expression of lncRNA and mRNA The Mann–Whitney *U* test was used to calculate significant differences between groups. **D** Analysis of GO term and KEGG enrichment for DELs *cis*-targets. **E** The statistical analysis of module-trait correlations. The modules and traits were indicated by the rows and columns, respectively. **F** Coexpression network diagram of hub lncRNA (MSTRG.109897.16) and protein coding genes. The green and orange circles represented MSTRG.109897.16 and protein coding genes, resepctively.

A total of 645 lncRNAs as DELs (*P*-value <0.05 and | log2foldchange | ≥1) were identified in L423 vs. L37 at five infection times. The DELs were the highest at 120 hpi and included 102 upregulated lncRNAs and 144 downregulated lncRNAs (Fig. 2A; Table S1, see online supplementary material). To explore the function of the DELs, the *cis-*regulatory role in mediating neighboring genes and the *trans*-regulatory role were predicted. We first analysed the 100 kb upstream and downstream regions of the 645 DELs for *cis* targets. A total of 431 lncRNA-mRNA pairs comprising 304 lncRNAs and 389 mRNAs were identified through co-expression analysis, in which 41 neighboring lncRNA–mRNA pairs had a positive correlation (Pearson’s correlation coefficient, |PCC| ≥0.5) (Fig. 2B; Table S2, see online supplementary material). Meanwhile, the correlation of neighboring lncRNA-mRNA pairs was significantly higher than that of the neighboring mRNA-mRNA, random lncRNA-mRNA, and random mRNA-mRNA pairs (Fig. 2C), which indicated that the lncRNAs may be able to positively upregulate their neighboring gene expression. Gene Ontology (GO) analysis showed that the lncRNA *cis* targets were enriched in the ‘glycosphingolipid biosynthesis’ and ‘gap junction’ categories. Additionally, KEGG analysis revealed that these *cis*-targets were largely representative of the ‘photosystem II assembly’ and ‘hormone catabolic process’ categories (Fig. 2D; Table S3, see online supplementary material).

In addition to the *cis-*targets, the lncRNA *trans-*targets were also predicted by weighted gene co-expression network analysis (WGCNA) of the DELs and DEGs [45]. Accordingly, we obtained 24 distinctly expressed modules (Fig. 2E). Among the modules, the MEdarkturquoise module was more closely related to *C. gloeosporioides* infection in L423 than that in L37 at five infection times, which was of particular interest in this study (Fig. 2E). The MEdarkturquoise module contained 106 lncRNAs and 1502 mRNAs, whose eigengene expression was significantly higher in L423 than in L37 (Fig. S1, see online supplementary material). The GO analysis showed that the genes in the MEdarkturquoise module were enriched in the ‘oxidation–reduction process’ and ‘defense response’ categories. The KEGG analysis revealed that these genes were substantially represented in the ‘plant-pathogen interaction’ and ‘glutathione metabolism’ categories (Fig. S2, see online supplementary material).

### Determination of hub lncRNA109897 and target gene JrCCR4

To examine the hub lncRNA in the MEdarkturquoise module significantly related to walnut disease resistance, we constructed a lncRNA-mRNA co-expression network. The co-expression network analysis showed that the hub lncRNA109897 (MSTRG.109897.16) had the highest connectivity in the network (Fig. S3, Table S4, see online supplementary material). Among the lncRNA109897-connected genes, the weight value between JrCCR4 (serine/threonine-protein kinase-like protein CCR4, XM_035695686.1) and lncRNA109897 was the highest (Fig. 2F; Table S4, see online supplementary material), indicating that there may be a positive regulatory relationship between lncRNA109897 and JrCCR4. Interestingly, in addition to the *trans* regulatory relationship, JrCCR4 is also the *cis*-target of lncRNA109897 (Fig. 2B; Table S2, see online supplementary material). These results are sufficient to provide an omics basis for subsequent analysis of the relationship between lncRNA109897 and JrCCR4 in walnut anthracnose resistance.

### LncRNA109897 positively regulates the expression of *JrCCR4*

**Figure 3:**
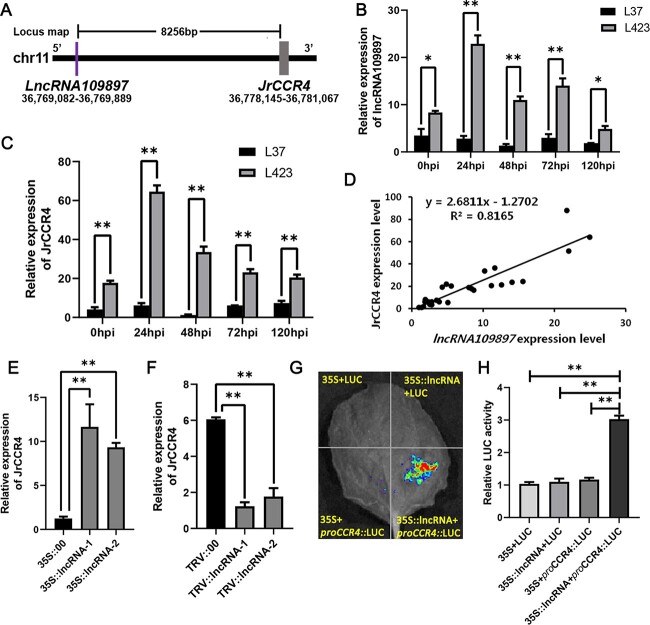
LncRNA109897 positively regulates the expression of *JrCCR4.***A** The location of lncRNA109897 and JrCCR4 on walnut chromosome 11. **B** and **C**, Relative expression of lncRNA109897 and JrCCR4 in L423 vs L37 in five infection time, respectively. **D** Correlation analysis of gene expression of lncRNA109897 and JrCCR4. The x-axis shows the relative expression of *lncRNA109897*. The y-axis shows the relative expression of *JrCCR4*. **E** and **F**, Relative expression of JrCCR4 in 35S::lncRNA109897 and TRV:: lncRNA109897, respectively. The 18S rRNA used as housekeeping gene, ^*^P < 0.05, ^**^P < 0.01. **G** and **H**, Dual-luciferase reporter assay between lncRNA109897 and proJrCCR4. Mean values ± SDs are shown from three biological replicates (*n* = 3).

To validate the putative relationships between lncRNA109897 and JrCCR4, their locational relationship was analysed and the expression levels were detected by qRT-PCR. LncRNA109897 (36769082–36 769 889) was located 8256 bp upstream of JrCCR4 (36778145–36 781 067) on chromosome 11 based on walnut genome annotation information (PRJNA291087) (Fig. 3A) [46]. The relative expression of lncRNA109897 and JrCCR4 exhibited the same trend during the process of infection of walnut by *C. gloeosporioides*, and the expression levels of lncRNA109897 and JrCCR4 in L423 were significantly higher than that in L37 (Fig. 3B and C). Additionally, the LncRNA109897 expression level was found to be positively correlated with the expression of its target gene JrCCR4 (*R*^2^ = 0.8165) (Fig. 3D). To further confirm that lncRNA109897 upregulates the expression of JrCCR4, we integrated the lncRNA109897-overexpressed vector (35S-lncRNA109897) and the virus-induced gene silencing (VIGS) vector (TRV-lncRNA109897) into the genome of walnut leaves. The transgene in 35S::lncRNA109897 and 4 TRV::lncRNA109897 was detected by the PCR amplification (Fig. S4, see online supplementary material). Compared with the control, the expression of JrCCR4 in walnut leaves overexpressing lncRNA109897 was significantly up-regulated. By contrast, in walnut leaves silenced with lncRNA109897, the expression of JrCCR4 was also down-regulated (Fig. 3E and F). Further, the luciferase reporter gene experiment (LUC) found that lncRNA109897 was covalently bound to the specific sequence in the target gene JrCCR4 promoter. Compared with the control, the co-expressed 35S:: lncRNA109897 and pro JrCCR4:: LUC showed stronger fluorescence signals in tobacco compared with pro JrCCR4:: LUC alone (Fig. 3G and H). These results showed that LncRNA109897 positively regulates the expression of target gene *JrCCR4.*

### LncRNA109897 and JrCCR4 enhance the resistance to *C. gloeosporioides*

**Figure 4:**
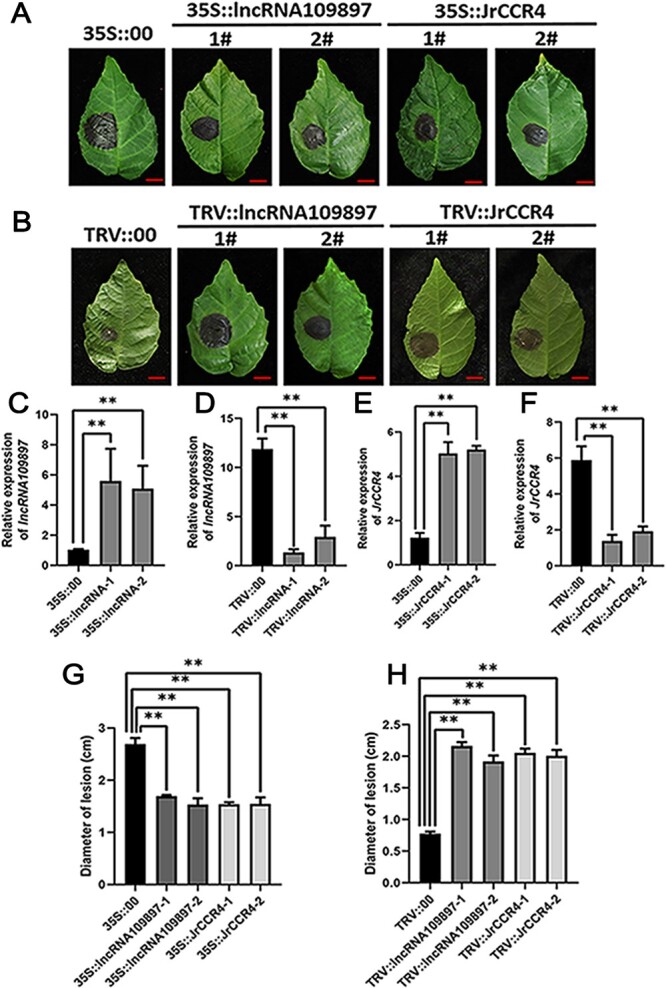
LncRNA109897 and JrCCR4 enhance the resistance of walnut to *C.gloeosporioides*. **A** Leaf phenotypes of 35S::lncRNA109897 and 35S::JrCCR4 walnut leaves infected with *C. gloeosporioides*. Bars = 1 cm. **B** Leaf phenotypes of TRV::lncRNA109897 and TRV::JrCCR4 walnut leaves infected with *C. gloeosporioides*. Bars = 1 cm. **C** and **D**, Relative expression of lncRNA109897 in 35S::lncRNA109897 and TRV::lncRNA109897 walnut leaves, respectively. **E** and **F**, Relative expression of JrCCR4 in 35S::JrCCR4 and TRV:: JrCCR4 walnut leaves, respectively. **G** Diameter of lesions in 35S::lncRNA109897 and 35S::JrCCR4 walnut leaves. **H** Diameter of lesions in TRV:: lncRNA109897 and TRV::JrCCR4 walnut leaves. The 18S rRNA used as housekeeping gene, *P < 0.05, **P < 0.01.

In an effort to preliminarily explore the effects of LncRNA109897 and JrCCR4 on the resistance of walnut to *C. gloeosporioides*, we obtained transgenic ‘B37’ walnut plants with transient overexpression of LncRNA109897 and JrCCR4 (Fig. 4A), and the lncRNA109897- and JrCCR4-specific fragments were also designed for VIGS experiments in L423 (Fig. 4B), which were proved to have been successfully introduced into walnut leaves by qRT-PCR test (Fig. 4C–F) and PCR amplification (Figs S4 and S5, see online supplementary material). Next, we examined the disease phenotype of the different transgenic materials. Leaves of the 35S::lncRNA109897 and 35S::JrCCR4-transgenic walnut inoculated with *C. gloeosporioides* exhibited fewer disease symptoms than the 35S::00-transgenic walnut leaves (Fig. 4A). Meanwhile, these 35S::lncRNA109897-transgenic and 35S::JrCCR4 walnut leaves also had smaller lesions than the 35S::00-transgenic walnut leaves (Fig. 4G). By contrast, the TRV::lncRNA109897-treated and TRV::JrCCR4-treated walnut leaves had more symptoms of disease and presented larger lesions than the control (Fig. 4B and H).

In addition, we generated transgenic lines that overexpressed lncRNA109897 (OE-lncRNA109897) and JrCCR4 (OE-JrCCR4) in the wild-type *Arabidopsis thaliana* (Fig. S6A, see online supplementary material). Both the OE-lncRNA109897 and OE-JrCCR4 transgenic lines were evaluated (Fig. S7, see online supplementary material), of which OE-lncRNA109897 lines 6 and 10 showed notably changed expression of lncRNA109897 through qRT-PCR and PCR (Fig. S6B and D, see online supplementary material). Similarly, OE-JrCCR4 lines 7 and 14 changed significantly at both the transcriptional and protein levels (Fig. S6C, E and F, see online supplementary material). After *C. gloeosporioides* infection, the lesion sizes of the OE-lncRNA109897 and OE-JrCCR4 transgenic line leaves were significantly smaller than those of the WT, which suggested that OE-lncRNA109897 and OE-JrCCR4 could enhance the resistance to *C. gloeosporioides* (Fig. S6A, see online supplementary material). These results demonstrated that lncRNA109897 and JrCCR4, as positive regulators, participate in walnut anthracnose resistance.

### JrCCR4 interacts with JrTLP1b *in vitro* and *in vivo*

**Figure 5:**
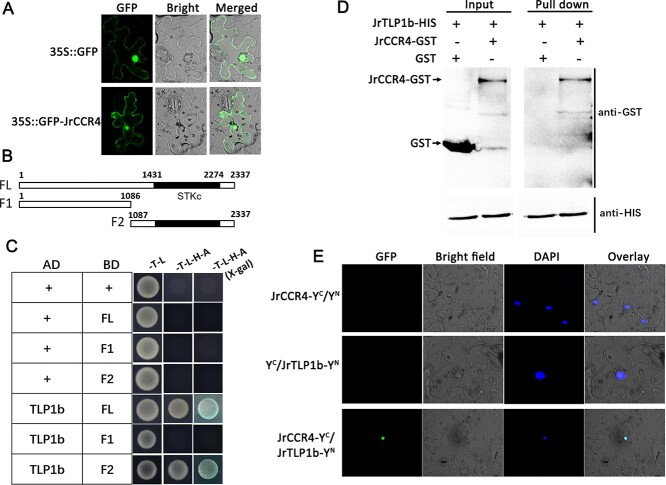
JrCCR4 interacts with JrTLP1b. **A** Subcellular localization of JrCCR4. **B** The CDS of JrCCR4 is divided into fragment 1 and fragment 2. **C** Y2H between JrCCR4 and JrTLP1b. **D** Pull-down assay between JrCCR4 and JrTLP1b. **E** BiFC between JrCCR4 and JrTLP1b.

JrCCR4 is a serine/threonine-protein kinase-like protein that contains a 2337-bp ORF encoding 778 amino acids. We compared JrCCR4 with its homologs in other plants, and the alignment of the amino acid sequences showed that the Ser/Threonine protein kinase domain was conserved at the N-terminus (Fig. S8, see online supplementary material). Subcellular localization of 35S::JrCCR4-GFP was carried out in tobacco cells, and the greener fluorescence signals were displayed in the nucleus and cell membrane of tobacco cells (Fig. 5A). To explore the potential proteins that interact with JrCCR4, a yeast two-hybrid (Y2H) screening assay was performed using the JrCCR4 protein as the bait to prey possible interaction partners from the cDNA library of walnut. As a result, the thaumatin-like protein JrTLP1b (LOC109012599) was filtered as a potential JrCCR4 interacting protein. The *JrTLP1b* gene contained a 699-bp ORF encoding 232 amino acids.

To verify the interaction between JrCCR4 and JrTLP1b, the full-length coding region of JrCCR4 was cloned and inserted into the pGBKT7 vector. Then, *JrTLP1b* was expressed from the pGADT7 vector. Yeast cell growth and blue-color precipitate were detected in yeast containing JrTLP1b-AD+JrCCR4-BD, indicating the interaction between JrCCR4 and JrTLP1b (Fig. 5C). Meanwhile, to identify the region of JrCCR4 that is responsible for its interaction with JrTLP1b, we divided JrCCR4 into two fragments (F1–F2), and F2 contained the STKc domain compared with F1 (Fig. 5B). The results indicated that the STKc domain in F2 determines its interaction with JrTLP1b (Fig. 5C). The recombinant protein of JrTLP1b with HIS tag was co-purified with the protein that only expressed GST and the recombinant protein of JrCCR4-GST, respectively, and the pull-down assays were performed. JrCCR4 was observed to be pulled down by JrTLP1b, which also indicated that JrCCR4 interacts with JrTLP1b (Fig. 5D). Bimolecular fluorescence complementation (BiFC) assays further confirmed that JrCCR4 interacts with JrTLP1b *in vivo* (Fig. 5E).

### JrCCR4 activates JrTLP1b transcriptional activity and stabilizes the protein

**Figure 6:**
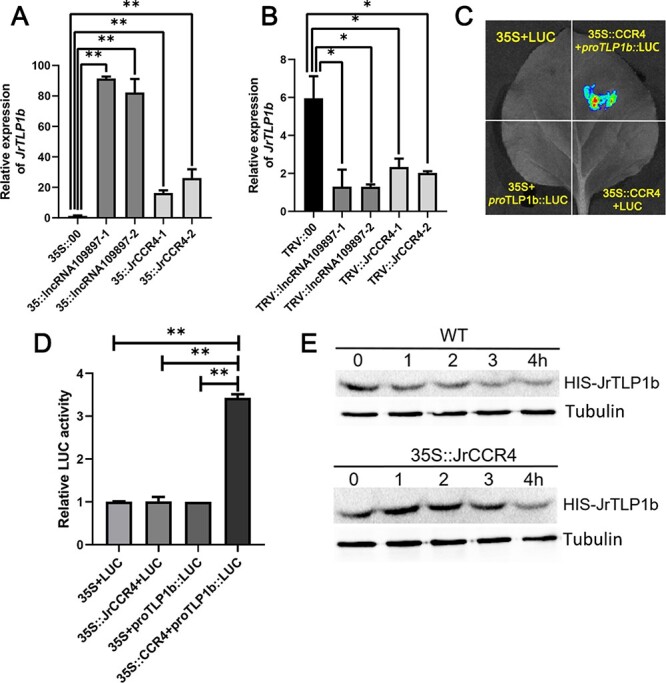
JrCCR4 activates the JrTLP1b transcriptional activity and stabilizes protein. **A** Relative expression of JrTLP1b in 35S::lncRNA109897 and 35S::JrCCR4 walnut leaves. **B** Relative expression of JrTLP1b in TRV::lncRNA109897 and TRV::JrCCR4 walnut leaves. **C** and **D**, Dual-luciferase reporter assay between JrCCR4 and pro JrTLP1b. **E** Cell-free degradation assay of JrTLP1b-HIS in WT and 35S::JrCCR4 transgenic *Arabidopsis thaliana*.

To examine whether JrTLP1b transcriptional activity is mediated by JrCCR4, we analysed the JrTLP1b expression level in 35S-JrCCR4 and TRV-JrCCR4. Compared with the control, the expression of JrTLP1b in 35S-JrCCR4 and TRV-JrCCR4 walnut leaves was significantly increased and decreased, respectively. Interestingly, the expression of JrTLP1b also increased and decreased in the 35S-lncRNA109897 and TRV-lncRNA109897 walnut leaves, respectively (Fig. 6A and B). Accordingly, we transiently co-infiltrated *N. tabacum* leaves with encoding JrCCR4 effector constructs and the LUC reporter gene fused with JrTLP1b promoters. Compared with the other three co-expressed controls, the transient expression of JrCCR4 significantly affected the relative LUC activity of the construction of JrTLP1b promoter (Fig. 6C and D). These results demonstrated that JrCCR4 activates JrTLP1b transcriptional activity.

In addition, to check whether the stability of JrTLP1b protein is affected by JrCCR4 protein, the prokaryotic expressed and purified JrTLP1b-HIS was co-incubated with the total protein extracted from WT and the 35S::JrCCR4-GFP transgenic *A. thaliana* at five time points, respectively. Subsequently, protein degradation assays showed that the stability of JrTLP1b protein was stronger in the protein extract of JrCCR4 transgenic *A. thaliana* than in WT (Fig. 6E).

### JrTLP1b enhances the resistance of *C. gloeosporioides*

**Figure 7:**
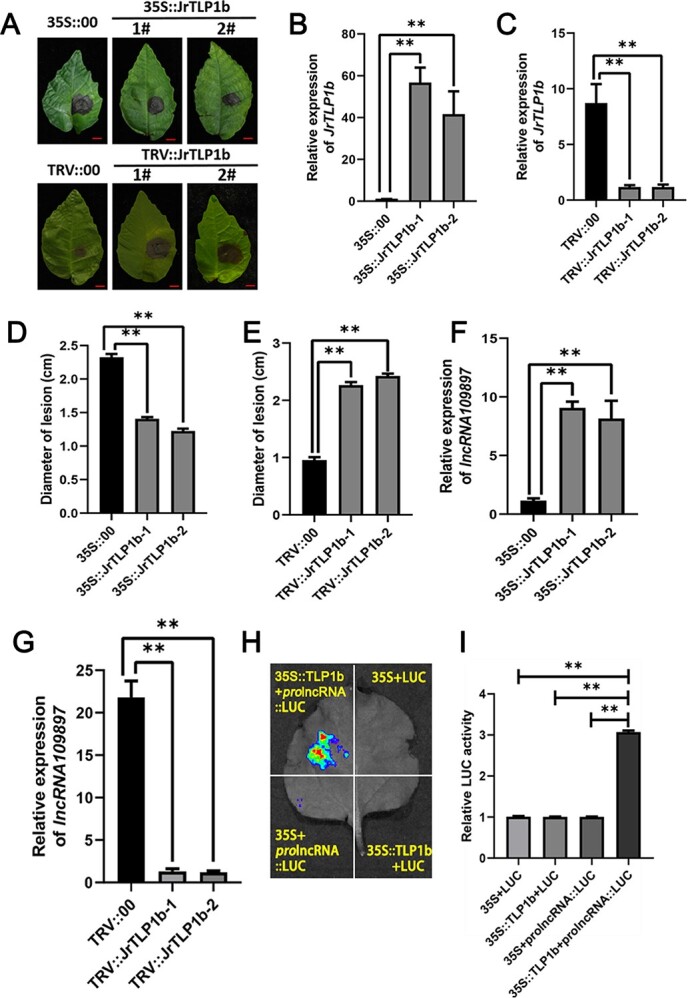
JrTLP1b enhances disease resistance of walnut and positively regulates lncRNA109897 transcription. **A** Phenotype of 35::JrTLP1b and TRV::JrTLP1b walnut leaves infected with *C. gloeosporioides*. Bars = 1 cm. **B** Relative expression of JrTLP1b in 35S::JrTLP1b walnut leaves. **C** Relative expression of JrTLP1b in TRV::JrTLP1b walnut leaves. **D** and **E**, Diameter of lesions in inoculated walnut leaves with 35S::JrTLP1b and TRV::JrTLP1b, respectively. **F** and **G**, Relative expression of lncRNA109897 in 35S::JrTLP1b and TRV::JrTLP1b walnut leaves. **H** and **I**, Dual-luciferase reporter assay showing the transcriptional activation of JrTLP1b to lncRNA109897.

JrTLP1b was identified as an interacting protein of JrCCR4, suggesting that it plays an important role in the plant defense system against *C*. *gloeosporioides*. To confirm its function in the process of walnut anthracnose resistance, 35S::JrTLP1b and TRV::JrTLP1b walnut leaves were obtained (Fig. 7A) and verified by qRT-PCR (Fig. 7B and C) and PCR amplification (Fig. S9, see online supplementary material). The pathogenicity test results showed that after inoculation of 35S::JrTLP1b-treated walnut leaves with *C. gloeosporioides*, the disease symptoms were less than empty vector treated walnut leaves, and the transgenic walnut leaves with transient overexpression of JrTLP1b showed less damage (Fig. 7A and D). By contrast, the TRV::JrTLP1b-treated walnut leaves had more symptoms of disease and bigger lesions than the control (Fig. 7A and E). Moreover, the OE-JrTLP1b lines 5 and 13 in the wild-type *A. thaliana* were obtained and evaluated, and *JrTLP1b* expression was altered significantly at both the transcriptional and protein levels (Fig. S10), see online supplementary material. After *C. gloeosporioides* infection, the lesion sizes of the OE-JrTLP1b transgenic lines leaves were significantly smaller than those of the WT (Fig. S10B, see online supplementary material). These results suggested that JrTLP1b enhances the resistance of walnut to anthracnose.

### JrTLP1b positively regulates lncRNA109897 transcription

To investigate whether JrTLP1b functions to upregulate lncRNA109897, we constructed a lncRNA109897-JrCCR4-JrTLP1b transcriptional loop to regulate the resistance of walnut to anthracnose. We first detected the expression of *lncRNA109897* in walnut leaves overexpressing JrTLP1b and silencing JrTLP1b through qRT-PCR. In the cDNA of walnut leaves with increased expression of JrTLP1b, the expression of lncRNA109897 was contrary to the result of VIGS, which also increased significantly (Fig. 7F). The opposite results were observed in the *JrTLP1b*-silenced walnut leaves (Fig. 7G). Next, to verify the effects of JrTLP1b on the promoter activity of lncRNA109897, we performed the LUC assay in *N. tabacum* leaves with the promoter sequence of lncRNA109897 and the CDS of JRTLP1b fused with pGreen II 0800-LUC and pGreen II 62-SK vectors, respectively.

Compared to the individual expression of prolncRNA109897::LUC, the co-expression of 35S::JrTLP1b with prolncRNA109897::LUC had remarkably stronger luminescence intensity (Fig. 7H–I). To summarize, these results suggest that JrTLP1b induces the expression of lncRNA109897.

## Discussion

Walnut anthracnose caused by *C. gloeosporioides*, which can cause fruit gangrene and leaf loss, is a catastrophic disease, and decreases the production of walnut [26]. In our previous study, it was found that walnut anthracnose was regulated by JrWRKY21, in which JrWRKY21-JrPTI5L-JrPR5L forms a transcriptional cascade and positively regulates walnut disease resistance [31, 32]. Recent research has shown that lncRNAs also participate in the control of walnut anthracnose resistance [30], but the function of lncRNAs in walnut disease resistance is unclear. In this study, we carried out strand-specific paired-end deep sequencing of resistant (4–23) and susceptible (B37) cloned leaf samples collected at different time points after *C. gloeosporioides* infection and obtained 645 DELs and 13 659 DE mRNAs. Our data show that there are fewer exons and shorter lengths in lncRNAs compared with the mRNAs, which is consistent with previously reported lncRNA characteristics [47, 48]. We then screened a hub lncRNA (MSTRG.109897.16, LncRNA109897) with the highest connectivity in the walnut anthracnose resistance-related module through WGCNA and qRT-PCR. To explore the role of lncRNA109897 in walnut anthracnose resistance, we carried out inoculation experiments on silenced and overexpressed lncRNA109897 walnut leaves and heterologous lncRNA109897-transformed *A. thaliana* leaves. The results showed that lncRNA109897 positively regulated walnut resistance to anthracnose, but its specific mechanism needs further study.

Accumulating evidence has shown that lncRNAs regulate gene expression in both a *trans* and *cis* way to participate in various plant biological processes [49]. In apple (*Malus domestica*), the high-light-induced MdLNC610 is a positive regulator, which can not only promote the expression of MdACO1, but also promote ethylene biosynthesis [50]. In cotton, *GbPMEI13* was identified as the target gene of lncRNA7, which was under positive regulation disease resistance using VIGS, while the target gene of lncRNA2 was *GbPG12* that negatively regulates resistance to *V. dahliae* through auxin-mediated signaling [51]. In *Populus trichocarpa*, the salt-induced Ptlinc-NAC72 can up-regulate the expression of simultaneously *cis-* and *trans*-regulating salt-responsive genes *PtNAC72.A/B* through identifying the tandem elements (GAAAAA) in the PtNAC72.A/B 50 non-translated region (50-UTR) [52]. Here, we identified that the lncRNA109897 potentially *cis-* and *trans*-target JrCCR4 and determined that the lncRNA109897 expression level was positively correlated with that of JrCCR4. The expression of *JrCCR4* in overexpressed and silenced lncRNA109897 walnut leaves was significantly increased and decreased, respectively. Meanwhile, lncRNA109897 could activate the activity and transcription of the JrCCR4 promoter. These results showed that lncRNA109897 positively regulates the expression of the target gene *JrCCR4.*

The serine–threonine kinase plays an important part in the signal transduction pathway and activation of the plant defense mechanism [53]. Pto protein in tomato with race-specific resistance to *Pseudomonas syringae* pv. tomato is a serine–threonine protein kinase [54]. The regulatory network of the Pto response to plant defense is mediated by the interaction between Pto and Pto (Pti) proteins. Among these Pti proteins, Pto phosphorylates Pti1 at Thr231, providing further evidence of the interaction between the two proteins [55, 56]. Moreover, Pti4, an ERF transcription factor, interacts with Pto and binds to GCC-box *cis* elements of the PR gene promoter to induce expression [57]. In tobacco, *NrSTK* containing an ATP binding site and a serine/threonine protein kinase activation sequence was identified to enhance resistance to black shank [14]. In this study, we identified a serine/threonine protein kinase-like protein JrCCR4 and determined its function and regulatory mechanism. It was found that JrCCR4 improved the disease resistance of walnut by inoculating the transiently transformed walnut leaves and heterologously transformed *A. thaliana* leaves. Meanwhile, JrCCR4 interacts with JrTLP1b, and the STKc domain is required for the interaction. JrCCR4 can also reduce the protein degradation of JrTLP1b and promote its expression. The above evidence indicates that the lncRNA109897 target JrCCR4 regulates walnut disease resistance through interaction with JrTLP1b.

In addition, TLPs classified as the PR-5 protein family are associated with host defense and developmental processes in plants [17]. Many previous studies have shown that the transgenic lines of the *TLP* gene exhibit antifungal properties. In potato, the overexpression of gene *CsTLP* induced by *P. infestans* spores proved that it has disease resistance [20]. In sea-island cotton (*Gossypium barbadense* L.), the up-regulation expression of GbTLP1 proved that it showed extremely high resistance to *Fusarium oxysporum* [58]*.* Similar to the above results, walnut and *A. thaliana* leaves overexpressing *JrTLP1b* showed resistance to *C. gloeosporioides,* while walnut leaves with silenced *JrTLP1b* showed the reverse resistance in our study. Strikingly, the expression of lncRNA109897 was significantly increased in *JrTLP1b-* overexpressing walnut but was significantly decreased in silenced *JrTLP1b* walnut. Additionally, JrTLP1b could activate lncRNA109897 promoter activity and induce its expression and further research is needed to determine the binding of JrTLP1b to specific elements on the lncRNA109897 promoter.

**Figure 8:**
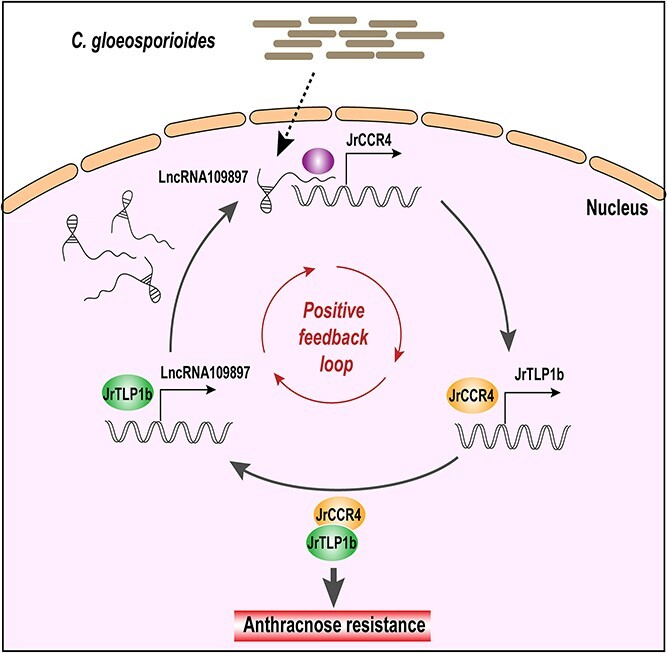
Proposed model for lncRNA109897-JrCCR4-JrTLP1b loop regulate walnut resistance to *Colletotrichum gloeosporioides.*

In summary, we have provided evidence that the lncRNA109897-JrCCR4-JrTLP1b positive feedback regulates walnut anthracnose resistance. In response to *C. gloeosporioides* infection, lncRNA109897 positively affects its target JrCCR4, thereby increasing the expression of *JrTLP1b* through interaction. In turn, JrTLP1b causes an increase in lncRNA109897 expression (Fig. 8). The identification of the loop regulation of lncRNA109897-JrCCR4-JrTLP1b is helpful for further understanding the disease resistance mechanism of walnut.

## Materials and methods

### Experimental materials, cDNA library construction, and RNA-seq

The transcriptome data applied for analysis in this study included the PRJNA776681 dataset we uploaded previously, the experimental materials, cDNA library construction, and the sequencing process, which has been depicted in our previous research [32].

### Identification and characterization of lncRNAs

The high quality clean reads without adaptors were mapped to the walnut genome (PRJNA291087) using HISAT 2.0.5 [46]. The transcriptomes obtained were assembled using StringTie (version 1.3.1) [59]. LncRNAs were identified premised on the screening procedure and previously described criteria [30]. According to their corresponding locations in the reference genome and the positional relationships between the lncRNAs and partner RNAs, the lncRNAs were sorted into intergenic, exonic, intronic, antisense, and divergent lncRNAs using multiple tools [60]. CIRCOS was used to visualize the localization and abundance of lncRNAs across walnut chromosomes [61]. In addition, the c-means was used to estimate the co-expression of pairwise transcripts between the lncRNAs and the nearest mRNAs [62]. The Pearson’s correlation coefficient, |PCC| ≥0.5, of the lncRNA-mRNA pairs was used to detect a positive or negative correlation.

### Expression analysis

For the expression analysis of lncRNAs and mRNAs, StringTie (v2.1.4) was used and the fragments per kilobase of exon per million fragments (FPKM) was calculated [63]. Ballgown (v2.20.0) was used for differential expression analysis. A *P*-value ≤0.05 and a |log2foldchange| ≥1 were used to designate differentially expressed lncRNAs and mRNAs.

### Network construction of co-expressed lncRNA-mRNA

A co-expression network of lncRNA-mRNA was formed using the WGCNA package (v1.67) as previously described [45] (https://horvath.genetics.ucla.edu/html/CoexpressionNetwork/Rpackages/WGCNA/). To optimize the network, we tested the different parameter values of the transcription data and determined the following parameter values: soft thresholding power, 12; minModuleSize, 30; split sensitivity, 2.0. The Module Eigengenes (MEs) of each module were calculated, and the correlation matrix between the module and the trait was plotted using the PHEATMAP package in R (www.r-project.org). The co-expression networks of the hub-lncRNA and mRNA in specific modules were obtained through Cytoscape software (v3.7.1) [64].

### Functional enrichment analysis

To understand the biological functions and metabolic pathways of the lncRNA target mRNA, GO and KEGG enrichment analyses were performed with topGO (v2.26.0) software and KOBAS (v2.0) software, respectively. The GO terms and KEGG metabolic pathways that satisfied *P* ≤ 0.05 were defined as a significantly enriched GO term and KEGG metabolic pathway, respectively. The *P*-value was corrected using the method of Benjamini–Hochberg.

### Quantitative RT-PCR analysis

The reverse transcription experiment was conducted using the TransScript® One-step gDNA Removal and cDNA Synthesis SuperMix kit (TransGen Biotech, Shanghai, China). The qRT-PCR was performed using the 2 × SYBR qPCR Master Mix. Take walnut 18S rRNA gene as the internal reference gene, and calculate the relative expression amount of the target gene according to the 2^−ΔΔCt^ (Software IQ5 2.0) [65]. The primers are shown in Table S5 (see online supplementary material).

### VIGS in walnut leaves and analysis of disease symptoms

Fragments specific to *lncRNA109897*, *JrCCR4*, and *TLP1b* were amplified from the cDNA of the ‘4–23’ walnut leaf. The three clones from pTRV2-lncRNA109897, pTRV2-JrCCR4, and pTRV2-TLP1b were transformed with the PYL156 vector. The primers are shown in Table S6 (see online supplementary material). Transfer the three recombinant products to *Agrobacterium tumefaciens* strain GV3101 to obtain recombinant strain. Briefly, 200 μL of the above obtained *A. tumefaciens* strain GV3101 solution containing the recombinant plasmids was combined with 2 mL YEP liquid, which is made up of 50 μg/mL Kan and 100 μg/mL Rif. Then, Add 2 mL of the turbid shaken bacteria to 100 mL of YEP liquid screening medium and then shaken at 28°C for 10–12 h. When the OD_600_ of the *A. tumefaciens* strain GV3101 solution containing the recombinant plasmids reached 1.0–1.5, the bacterial suspension is centrifuged. After resuspension (10 mmol/L MES, 10 mmol/L MgCl2, and 266.7 μM ACE) with heavy suspension, mix it fully according to 1:1. Place the prepared infection solution in the dark for 3 hours, and then carry out infection [66]. The *A. tumefaciens* lines containing the constructs were transformed into walnut ‘4–23’ leaves by vacuum infiltration. After 5 days, the strain ‘m9’ of *C. gloeosporioides*, which was cultured on PDA medium at 22°C for 7–12 days, infected the treated leaves. Fungal colony edges were transferred to the slightly injured leaves with a 5-mm diameter punch. After 5 days of infection, the changes of leaf lesions were recorded.

### Gene transient overexpression in walnut

The CDSs of lncRNA109897, JrCCR4, and TLP1b were linked with pRI-101 vectors to form recombinant plasmids. The primers are shown in Table S6 (see online supplementary material). The three recombinant products were introduced into *A. tumefaciens* (GV3101), and *A. tumefaciens* carrying the 35S::00, 35S::lncRNA109897, 35S::JrCCR4, and 35S::TLP1b constructs was infected into the ‘B-37’ walnut younger leaves. With GV3101 as the medium, the target genes were introduced into the test leaves, and then the disease was analysed. Three biological replicates were set for each treatment. Each biological repeat measured three leaves.

### Stable overexpression in *A. thaliana* leaves and analysis of disease symptoms

The spore suspension with OD value of 0.6 was obtained by referring to the *A. tumefaciens* infection method of walnut transient overexpression. After leaving for 3 h, an inflorescence of *Arabidopsis* was soaked in a bacterial suspension for 1 min. The infection method of the strain ‘m9’ on *Arabidopsis* leaves is similar to that of VIGS.

### Mating method for screening the yeast two-hybrid library

The protein coding sequence of the gene *JrCCR4* is inserted into the MCS sequence of the pGADT7 vector. The primers are shown in Table S6 (see online supplementary material). Yeast Y2H competent cells containing pGADT7 + JrCCR4 vector were co-cultured on SD/− T medium for 5 days. A 2–3 mm Y2H cold containing the bait plasmid was selected and freshly cloned into 60 mL SD/−T liquid medium. After culturing at 30°C with 230–250 rpm shaking culture overnight (16–20 h) until OD_600_ = 0.8, the solution was then centrifuged at 1200 *g* for 4 min, the supernatant was removed, and then the cells were suspended in 4–5 mL SD/−T liquid medium. One milliliter of AD and 4–5 mL of BD bacterial solution were added to a 2-L sterilized conical flask, following which 45 mL of 2 × YPDA liquid medium (containing 50 μg/mL Kan) was added, and then, the tube of the AD strain was rinsed with 1 mL 2 × YPDA twice and transferred into a conical flask. The solution was then cultured at 30–50 rpm at 30°C on a shaker for 20–24 h. Twenty hours later, micky head or clover-shaped binders were observed under a 40× microscope. After centrifuging at 1200 *g* for 8 min, the supernatant was removed to collect the thallus. The cells were suspended with 10 mL 0.9% NaCl (containing 50 μg/mL Kan+) to measure the total volume. The remaining mixture was coated on a SD/-H/−L/−T/X-α-Gal plate with a diameter of 150 mm and then placed at 30°C in a constant temperature incubator for 3–5 days. The blue clones on the primary sieve plate were transferred to SD/-H/−L/−T/X-α-Gal plates with higher screening strength for further screening. Positive clones on QDO/X were tested by Sanger sequencing and one-to-one validation.

### Yeast two-hybrid assay

The recombinant plasmid pGADT7 + TLP1b was constructed by linking the CDS of *TLP1b* obtained from the Y2H sieve library with the vector pGADT7. The primers are shown in Table S6 (see online supplementary material). After re-suspension and re-digestion with 0.9% NaCl, the yeast Y2H competent cells containing pGADT7 + JrCCR4 vector were coated in SD/−L/−T and SD/-H/−A/−T/−L medium for 5 days. Subsequently, the plaques grown in SD/−T/−L/-H/−A medium were re-suspended and dissolved with 0.9% NaCl and then coated in SD/-H/−A/−T/−L/X-α-Gal medium for 5 days to grow new plaques, so as to verify the interaction between the two proteins mentioned above.

### Pull-down assay

Add HIS tag and GST tag to two target genes *TLP1b* and *JrCCR4* respectively. The primers are shown in Table S6 (see online supplementary material). The tagged target gene *TLP1b* and *JrCCR4* is transferred into E.coli BL21Condon plus (DE3) to induce the corresponding protein. The corresponding proteins were purified with specific HIS- and GST-tagged (ComWin Biotech) protein purification kits. In the next step, the two proteins containing the His-Tag and GST-Tag were mixed at a ratio of 1:1 and purified with Glutathione-Sepharose Resin (ComWin Biotech). The purified proteins and mixed proteins were further verified by Western blotting.

### Subcellular localization of JrCCR4

A GFP tag that emits green fluorescence is inserted into the gene *JrCCR4*. A sterile syringe was used to introduce the GV3101-GFP-JrCCR4 into young tobacco leaves with good growth through GV3101-Psoup-P19, and the green fluorescence signal was observed using a confocal laser microscope LSM880 (Carl Zeiss, Oberkochen, Germany). The primers are shown in Table S6 (see online supplementary material).

### Dual-luciferase assays

The *lncRNA109897* sequences and *JrCCR4* promoter sequences were inserted into the pGreenII-62-SK vector and pGreenII-0800-LUC vectors, respectively, to form recombinant plasmids. The primers are listed in Table S6 (see online supplementary material). *A. tumefaciens* containing recombinant genes and blank vectors were mixed in pairs at 1:1, respectively, to form four mixed bacterial solutions. Then, they were injected into four positions of the same 28-day-old tobacco leaf. Living cell imaging apparatus was used to detect the luminescent signal on the back of tobacco. Dual-luciferase assays of JrCCR4-proTLP1b and TLP1b-proLncRNA109897 are the same as above.

### Protein degradation of plants *in vitro*

Total proteins were extracted from *A. thaliana* leaves using a Plant Protein Extraction Kit (ComWin Biotech, Beijing, China). Prokaryotic-induced proteins (TLP1b-HIS) were mixed with plant proteins (WT/JrCCR4-GFP) at a ratio of 1:3 and then placed in a metal bath at 22°C. Next, samples were taken at 0 h, 1 h, 2 h, 3 h, and 4 h. The protein levels of TLP1b-HIS were checked by Western blotting using the anti-HIS.

## Acknowledgments

This work is supported by the National Natural Science Foundation of China (32001340), the Improved Variety Program of Shandong Province of China (2020LZGC090102), and the Natural Science Foundation of Shandong Province (ZR2020QC169). We thank the laboratory of Chen Xuesen of College of Horticulture Sciences and Engineering, Shandong Agricultural University for providing plasmid and yeast strains.

## Author contributions

H.F. and K.Q.Y. devised and supervised the project. R.Z., Y.D., C.W., X.L., and S.X. performed the experiments. J.L. and Q.L. analysed the data. R.Z., Y.D. and H.F. wrote the original draft manuscript, R.Z. and Y.D. contributed equally to this work. X.M., W.L., S.Z., N.W., and K.Q.Y. reviewed and edited the manuscript. All authors reviewed and approved the manuscript.

## Data availability

The data that supports the findings of this study are available in this article and in the supplementary material of this article.

The sequence of lncRNA109897 has been deposited in the National Center for Biotechnology Information Sequence Read Archive (https://www.ncbi.nlm.nih.gov/sra/) with project number OP627665.

## Conflict of interest statement

The authors declare that there have no conflicts of interest associated with this work.

## Supplementary data

Supplementary data is available at *Horticulture Research* online.

## Supplementary Material

Web_Material_uhad086
