# Supplementary material for: LncRNA109897-JrCCR4-JrTLP1b forms a positive feedback loop to regulate walnut resistance against anthracnose caused by Colletotrichum gloeosporioides
Source: Hortic Res. 2023 May 3;10(6):uhad086. doi: 10.1093/hr/uhad086 (PMC10541558; doi:10.1093/hr/uhad086)
Supplement: Web_Material_uhad086 [file web_material_uhad086.zip › Supplemental Figure.pdf]

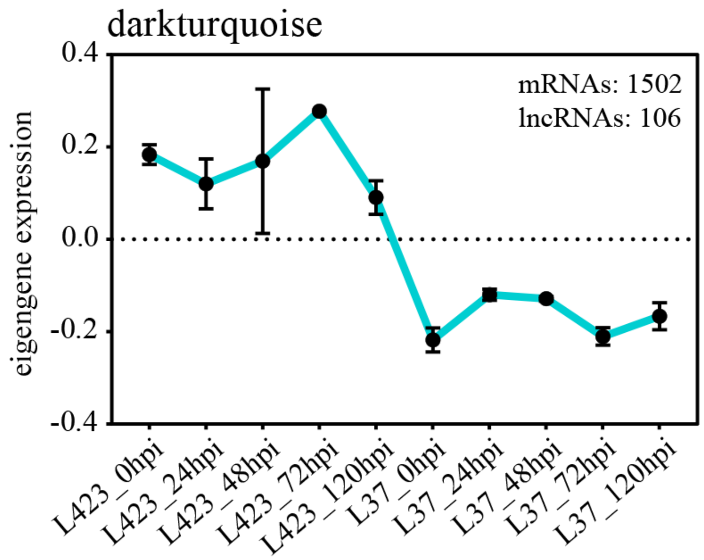

Supplemental Figure S1. The eigengene expression in MEdarkturquoise module.

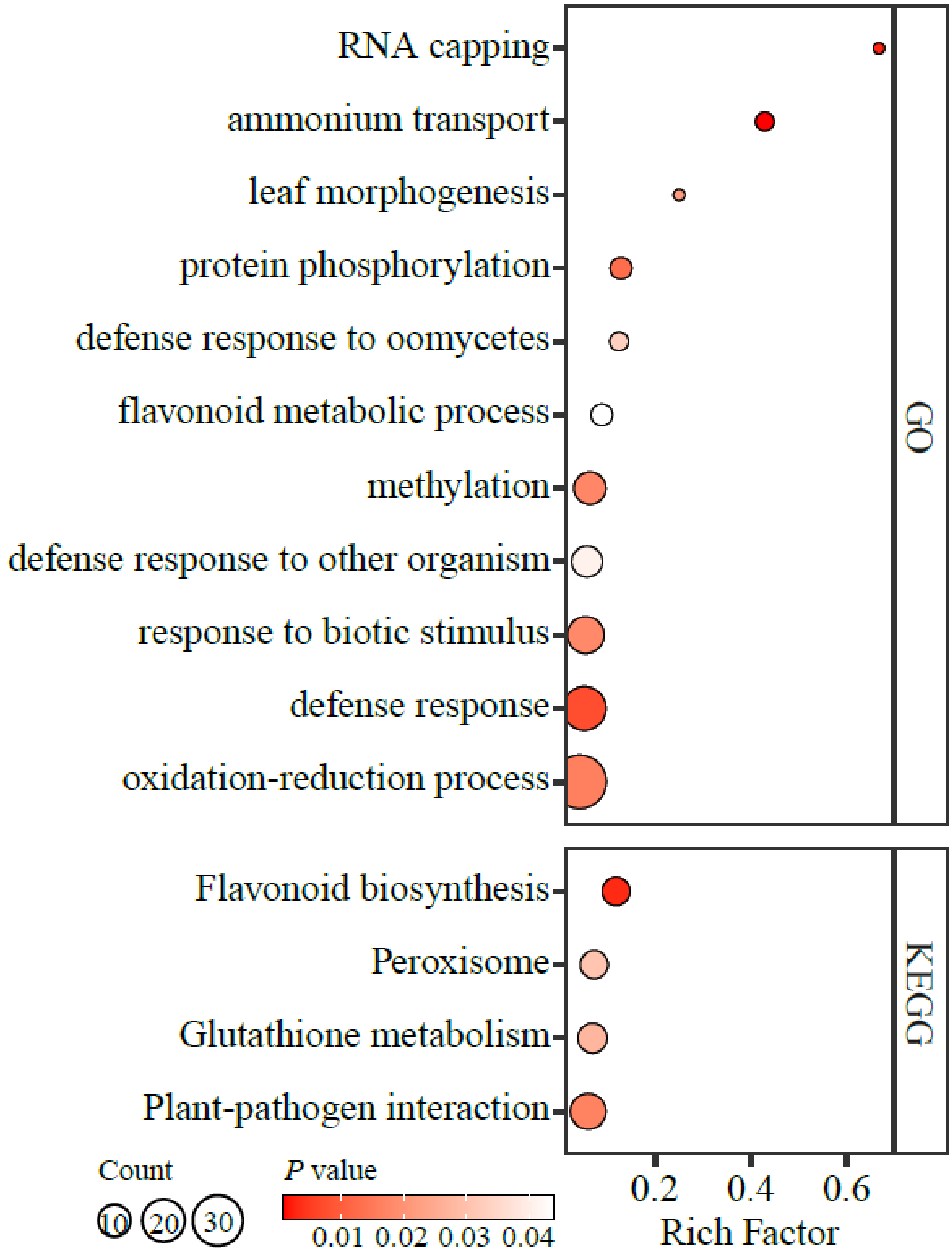

Supplemental Figure S2. GO/KEGG analysis in MEdarkturquoise module.

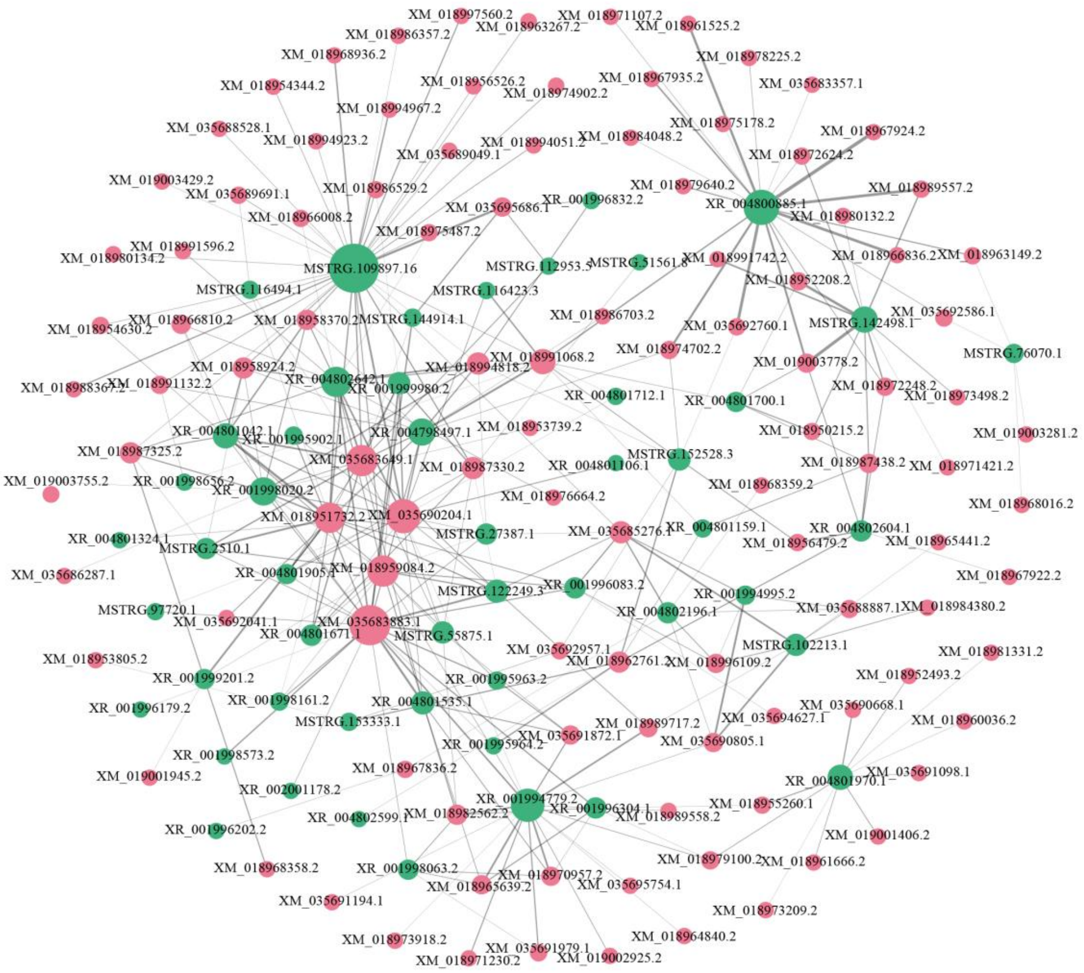

Supplemental Figure S3. Network of lncRNA-mRNA in MEdarkturquoise module.

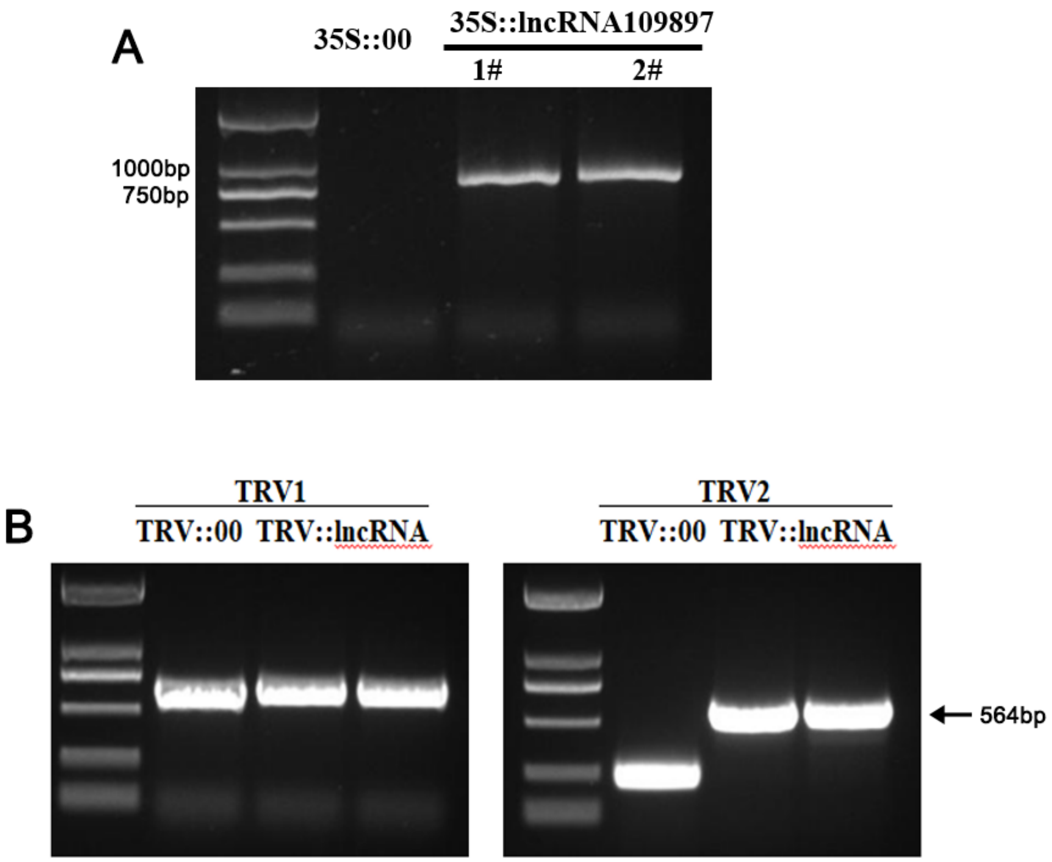

Supplemental Figure S4. (A). Presence of the transgene in 35S::lncRNA109897 confirmed by PCR amplification. (B). PCR identification of RNA1 and RNA2 of TRV::lncRNA109897 in walnut leaves.

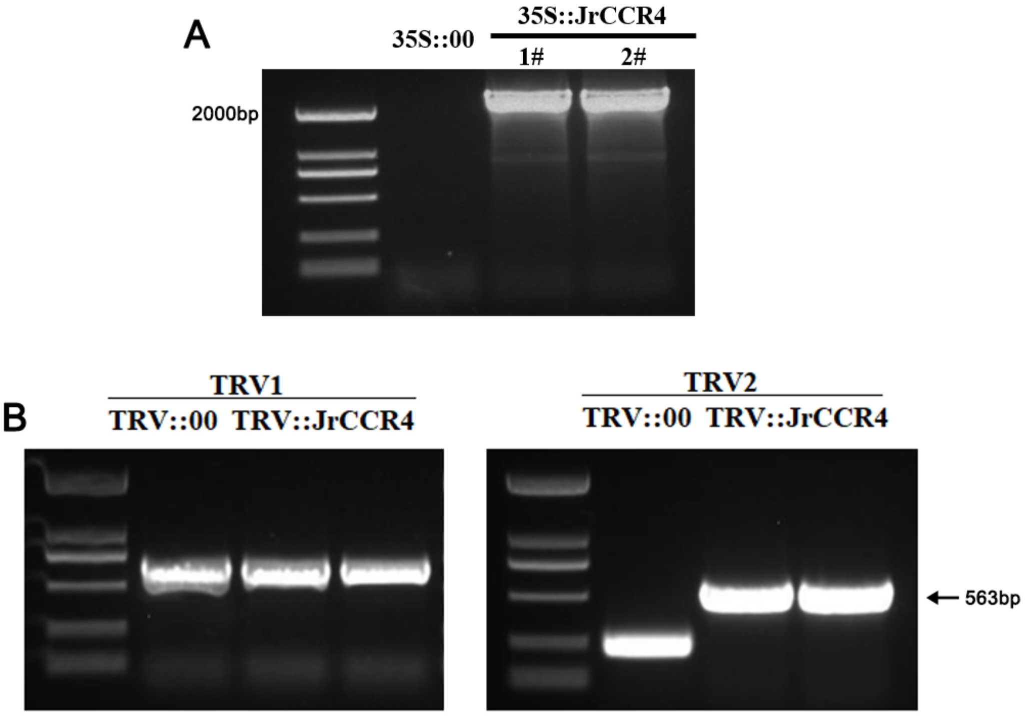

Supplemental Figure S5. (A). Presence of the transgene in 35S::JrCCR4 confirmed by PCR amplification. (B). PCR identification of RNA1 and RNA2 of TRV::JrCCR4 in walnut leaves.

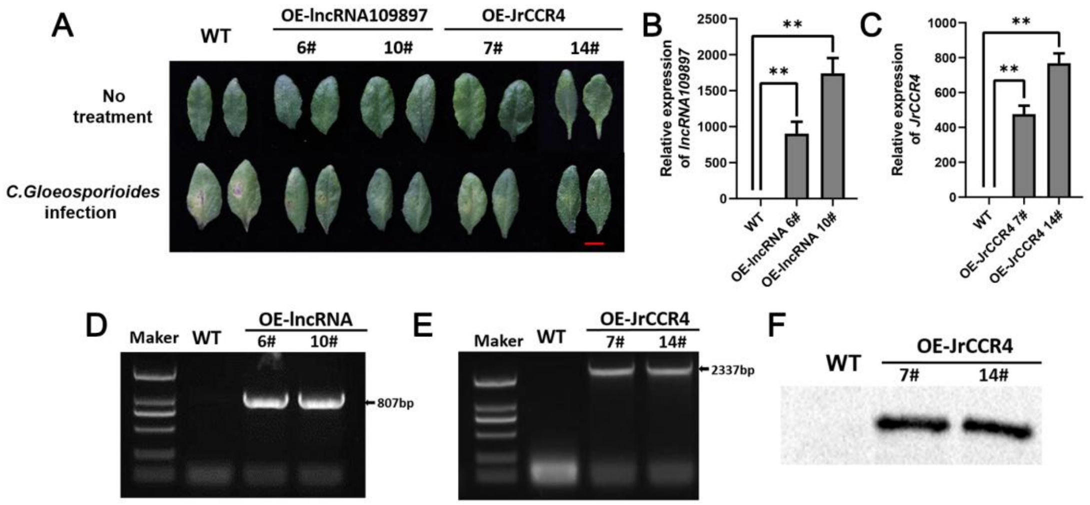

Supplemental Figure S6. Heterologous overexpressed lncRNA109897 and JrCCR4 *A.thaliana* enhances disease resistance. (A). Leaf phenotype upon infection by *C. gloeosporioides* in overexpressed lncRNA109897 and JRCCR4 *Arabidopsis thaliana*. Bars = 1cm. (B). Relative expression of lncRNA109897 in OE-lncRNA109897 *Arabidopsis thaliana*. (C). Relative expression of JrCCR4 in OE-JrCCR4 *Arabidopsis thaliana*. (D) and (E). Presence of the transgene in OE-lncRNA109897 and OE-JrCCR4 *Arabidopsis thaliana* confirmed by PCR amplification, respectively. (F). Presence of the transgene in OE-JrCCR4 *Arabidopsis thaliana* confirmed by Western blotting with GFP antibody. The AT3G18780 used as housekeeping gene, $*P < 0.05$, $**P < 0.01$.

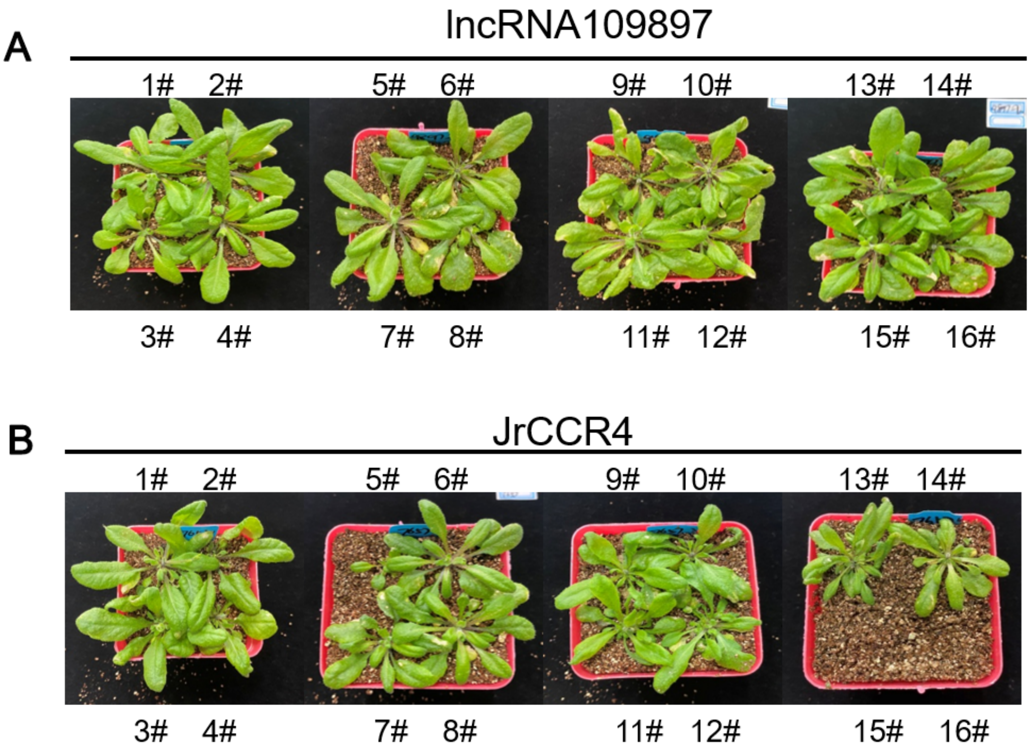

Supplemental Figure S7. (A). *Arabidopsis thaliana* infected by *A.tumefaciens* containing overexpressed lncRNA109897. (B). *Arabidopsis thaliana* infected by *A.tumefaciens* containing overexpressed JrCCR4.

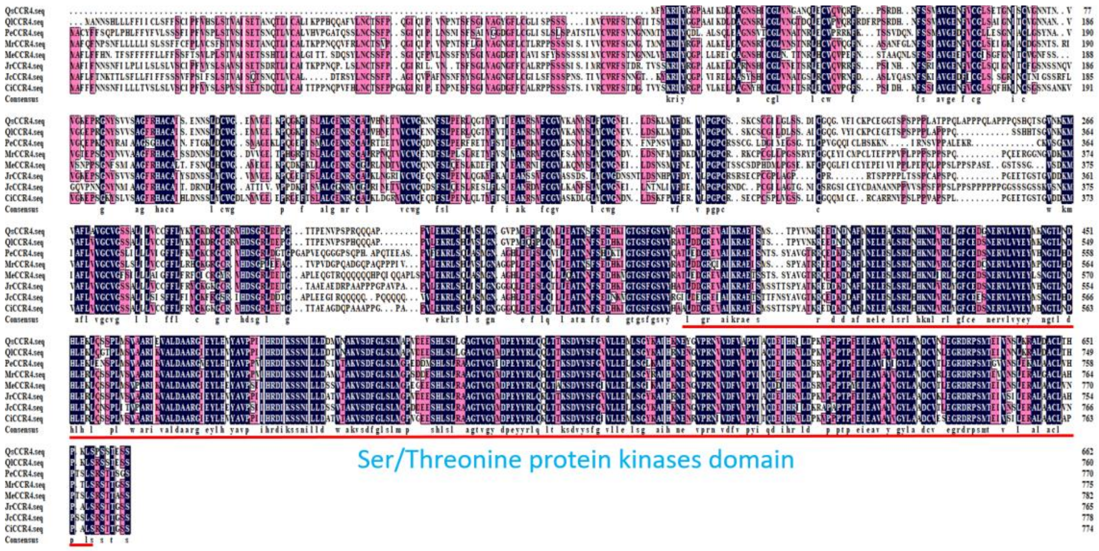

Supplemental Figure S8. Amino acid sequence alignment of eight JrCCR4 proteins including the STKs domain.

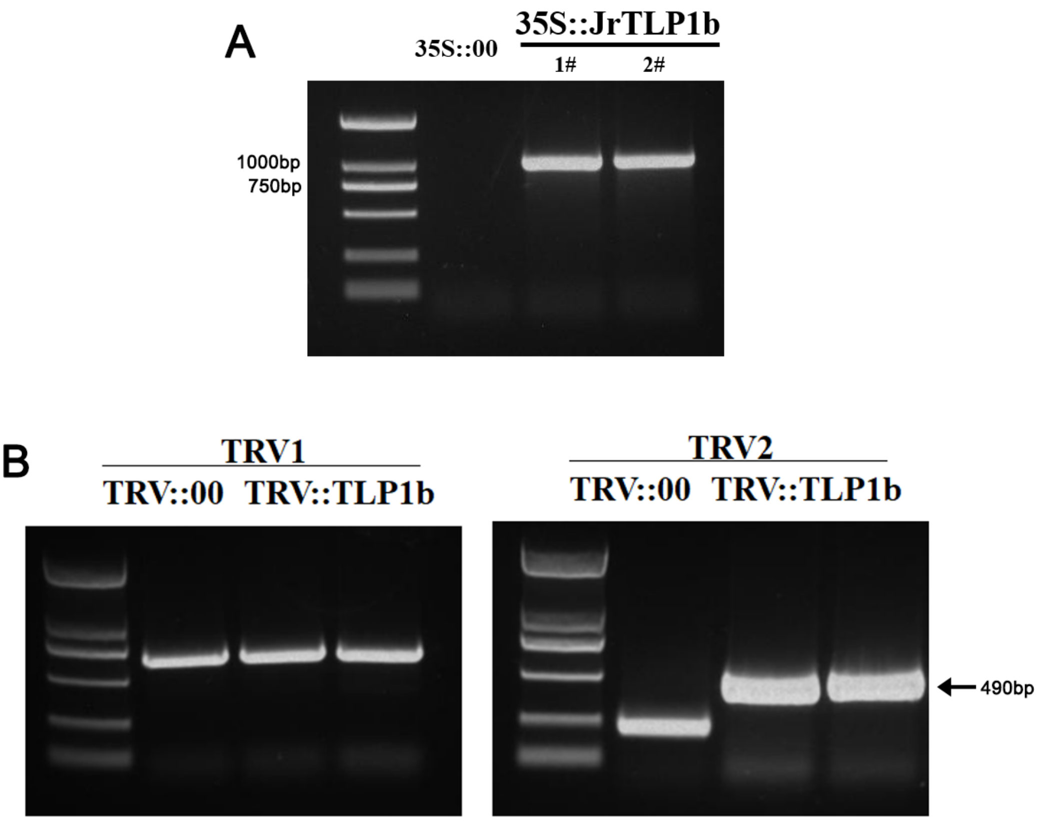

Supplemental Figure S9. (A). Presence of the transgene in 35S::JrTLP1b confirmed by PCR amplification. (B). PCR identification of RNA1 and RNA2 of TRV::JrTLP1b in walnut leaves.

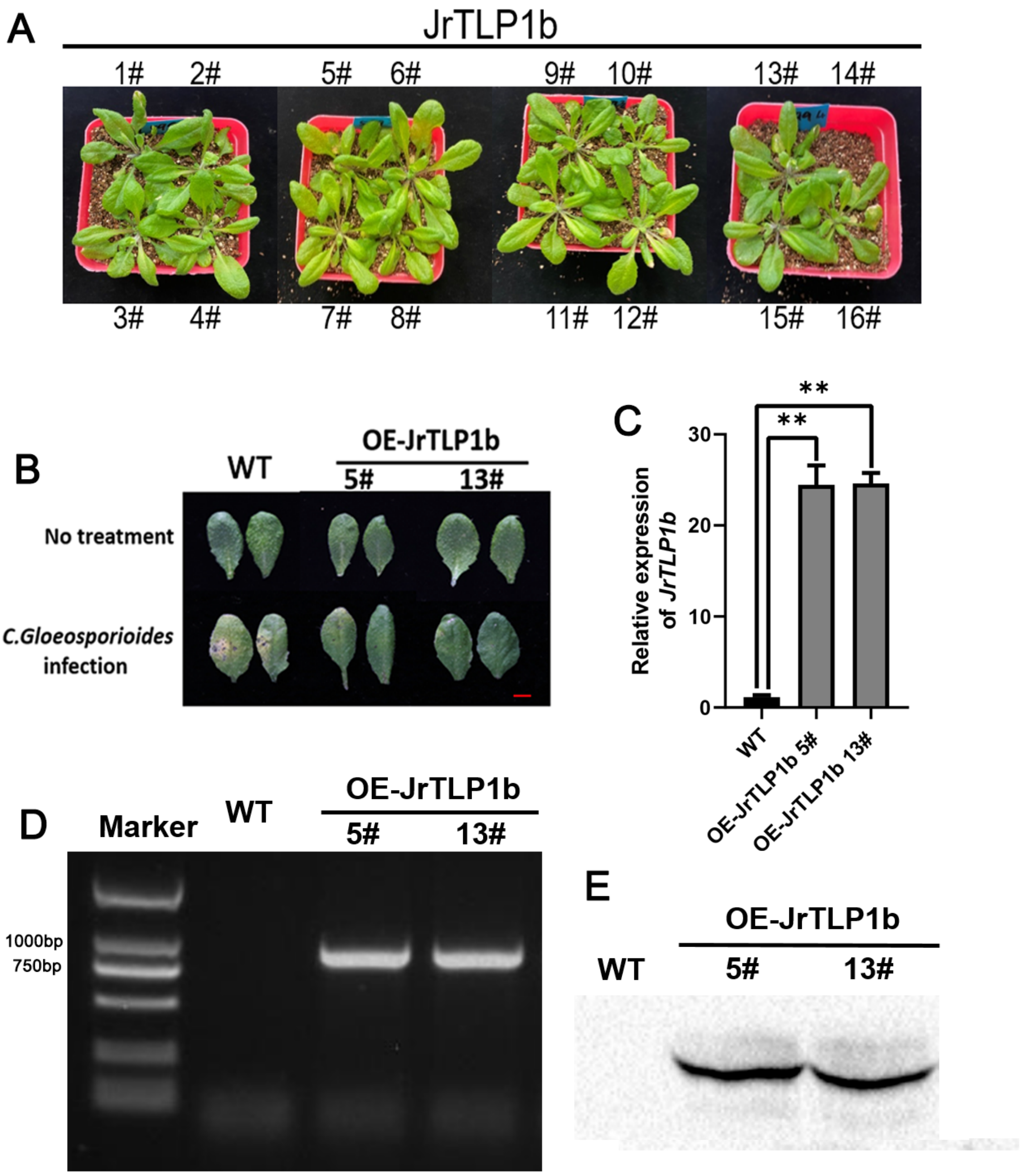

Supplemental Figure S10. (A). *Arabidopsis thaliana* infected by *A.tumefaciens* containing overexpressed JrTLP1b. (B). Leaf phenotype upon infection by *C. gloeosporioides* in overexpressed *JrTLP1b Arabidopsis thaliana*. Bars = 1cm. (C). The relative expression of *JrTLP1b* genes. (D). Presence of the transgene in OE-JrTLP1b *Arabidopsis thaliana* confirmed by PCR amplification. (E). Presence of the transgene in OE-JrTLP1b *Arabidopsis thaliana* confirmed by Western blotting with GFP antibody.
