# Supplementary material for: LncRNA109897-JrCCR4-JrTLP1b forms a positive feedback loop to regulate walnut resistance against anthracnose caused by Colletotrichum gloeosporioides
Source: Hortic Res. 2023 May 3;10(6):uhad086. doi: 10.1093/hr/uhad086 (PMC10541558; doi:10.1093/hr/uhad086)
Supplement: Web_Material_uhad086 [file web_material_uhad086.zip › Supplemental Table.pdf]

Table S1. Identification of differentially expressed lncRNAs										
LncRNA	logFC(0hpi)	P value(0hpi)	logFC(24hpi)	P value(24hpi)	logFC(48hpi)	P value(48hpi)	logFC(72hpih)	P value(72hpi)	logFC(120hpi)	P value(120hpi)
MSTRG.100220.2	-0.09945129	0.134133448	0.744772838	0.376016787	-0.11903858	0.08775519	0.004587528	0.930923397	-1.011908223	0.03916212
MSTRG.100779.1	-0.137603726	0.042114993	-0.19129633	0.208347407	-	-	0.085841584	0.427418	1.062063662	0.015225501
MSTRG.100871.1	1.98278039	0.026463192	-0.00815119	0.996109528	3.652766609	0.217276273	3.797341795	0.007739277	1.593801566	0.468812854
MSTRG.102091.1	-0.539935169	0.001139379	-1.07083367	0.179635238	-0.34649494	0.663016577	-2.52002655	0.014945396	0.390273626	0.599461097
MSTRG.102138.1	0.233221182	0.161790267	0.932994429	0.156932914	2.063931159	0.128269013	0.597339501	0.06969473	-1.209086753	0.045060281
MSTRG.102213.1	2.166075553	8.94592E-05	2.466512226	0.00394491	3.694004141	0.229617978	2.814215718	0.003701372	4.352898155	0.000165591
MSTRG.104630.1	-1.423713879	0.003524658	0.226036707	0.496753493	-0.053086	0.602588772	-	-	-1.579535438	0.118374249
MSTRG.104704.1	1.488879363	0.001492346	-0.04118623	0.954499249	-0.76621821	0.697133444	0.410164746	0.000582652	0.387302839	0.043826916
MSTRG.104758.2	-0.169163159	0.179310758	1.245228191	0.037058384	-0.11904523	0.846758304	0.57647439	0.010964065	-2.290908854	0.017475216
MSTRG.104916.7	1.035032477	0.01785127	0.281541919	0.660094989	1.057875502	0.494821095	-0.75853381	0.005817794	-1.319580572	0.106865342
MSTRG.105423.1	-0.002925959	0.959652585	-0.26039666	0.261422393	0.147899458	0.45012819	0.023812244	0.091352728	-1.189705498	0.012530144
MSTRG.106722.1	0.124975066	0.344546161	-0.85708927	0.086265949	-0.70290631	0.039435426	0.23554022	0.053999706	1.627043578	0.00115022
MSTRG.108408.1	0.123819646	0.229765246	0.008632557	0.915205076	-0.05847881	0.52924194	0.007664868	0.807747809	1.029611456	0.013735615
MSTRG.10866.1	0.097315828	0.675305009	0.450568633	0.413378833	-1.29718065	0.043346082	-0.17698125	0.309462455	-0.741363902	0.111022994
MSTRG.109398.1	-	-	2.013744851	0.00035119	-	-	-	-	-1.336202201	0.463274466
MSTRG.109627.1	0.098274435	0.312964181	0.254441528	0.049688161	0.10121648	0.000117329	0.064182113	0.336438975	-1.081678005	0.016601866
MSTRG.110728.1	-0.387974737	0.004968327	-0.16529904	0.208465342	0.253173124	0.111514649	-0.06969894	0.174275602	-1.12683652	0.000662038
MSTRG.111024.2	-0.364926255	0.017586754	-0.5003505	0.071571706	-	-	-	-	-1.02199591	0.000268973
MSTRG.111553.2	0.072971102	0.047807957	1.167943058	7.72726E-06	0.263317766	0.125493256	0.133214547	0.006855951	0.195798109	0.623253982
MSTRG.111599.5	-0.034853511	0.469964316	-1.11742685	0.033575382	0.173101545	0.079048308	0.153060698	0.001956135	0.138797151	0.769178269
MSTRG.112032.1	0.065770315	0.497889553	-1.04210816	0.018540189	0.498513274	0.211272444	0.059685557	0.347464289	1.130882716	0.001964471
MSTRG.112953.1	-0.057312956	0.218171109	0.307175707	0.043375048	0.604185705	0.212182088	0.263359488	0.188013693	1.013081055	0.037486921
MSTRG.112953.5	0.851195014	0.238201307	0.23925032	0.008354954	0.401674616	0.489933208	0.708220596	0.053885691	1.25920744	0.00372419
MSTRG.113479.1	-0.389308926	0.060045491	0.246033937	0.384354913	1.278831528	0.001539339	0.131382802	0.38922808	-1.121654916	0.006017327
MSTRG.114013.2	1.141492326	0.251647745	1.090678473	0.000418575	0.066765832	0.196453442	0.028167367	0.001654715	0.00498983	0.622532362
MSTRG.114169.1	0.175931494	0.326723489	1.086247574	0.029189665	0.054420989	0.851117385	-0.04354231	0.643515549	-0.840050854	0.012310958
MSTRG.11420.2	-	-	-2.67978448	0.016666618	-2.48018355	0.004809387	0.648850907	0.134669294	0.979421216	0.129578112
MSTRG.11420.3	-3.886051138	0.000948772	-5.14446096	0.025324865	-3.94294512	1.44711E-06	-4.96212208	0.000561266	-0.722094946	0.751521413
MSTRG.115446.1	0.299993375	0.068026676	-0.73285198	0.373550069	1.795269248	0.03682011	-0.25672306	0.603572877	-0.06351993	0.946877849
MSTRG.115465.1	-0.517611185	0.000527079	-0.88465969	0.122330689	-0.28306892	0.062318015	-0.03686117	0.908994129	-1.163464712	0.034837478
MSTRG.116423.3	1.382427489	0.003591836	0.533997074	0.039774075	1.091117902	0.000339739	0.954807405	0.005118447	-0.657399004	0.549931581
MSTRG.116494.1	-0.633751275	0.000331476	-0.56831086	0.027902177	-0.18147527	0.645016927	-0.84663848	3.92409E-05	-1.040088492	3.26135E-05
MSTRG.116544.1	-0.080026636	0.455935543	1.056072211	0.00229947	0.094939218	0.476972026	-0.13278972	0.047215195	0.251865033	0.276938097
MSTRG.117908.1	0.063391466	0.625205809	1.068473203	0.007713508	0.37364514	0.167312208	0.040116332	0.539224497	-0.042850644	0.606824866
MSTRG.11836.1	-0.083211044	0.200210301	-0.65247155	0.013402937	0.291954976	0.075404594	-0.06150867	0.214454935	1.125934556	0.012280588
MSTRG.118412.2	0.894658496	0.210729414	-1.05849867	0.240456976	-0.67642003	0.608291094	1.312124525	0.005108888	1.405506654	0.090380165
MSTRG.118412.3	0.933677673	0.287434936	2.539098743	0.022931827	2.029207214	0.014200208	0.648961353	0.054004699	-0.633172529	0.438849667
MSTRG.11846.1	4.254642591	2.738E-06	3.409955694	0.000125067	1.01330937	0.44577192	0.88862059	0.002707615	3.717373992	0.226482056
MSTRG.118627.1	0.158698942	0.083248057	-1.09276537	0.042115689	-0.05377279	0.73934212	-0.02788108	0.290239934	0.166172809	0.05889462
MSTRG.118963.1	0.082905705	0.397877063	-0.01458182	0.922745136	-	-	-0.07194594	0.26200382	-1.191296386	0.028169071
MSTRG.119382.4	-0.001540209	0.571967754	-0.03148183	0.04293404	-	-	-	-	-1.169813127	0.000902542
MSTRG.119507.1	-0.153570684	0.037275786	0.483532086	0.22305237	-	-	-0.448581	0.10265631	-1.064019293	0.006136321
MSTRG.119959.1	-0.119880221	0.079562397	-0.10458144	0.258271082	-0.58209618	0.024745132	0.048467932	0.200940037	-1.266592694	0.008300103
MSTRG.12027.2	-0.140646185	0.013627344	-0.11038113	0.020074353	-	-	-0.08126006	0.149464817	-2.100824427	0.019244857
MSTRG.12031.1	-0.149940292	0.003096321	-0.1193749	0.049566117	0.027941618	0.556180386	-0.10243043	0.100814293	-1.087315069	0.00228761
MSTRG.12034.1	-0.227536064	4.48068E-05	-0.07046825	0.455457	-	-	-0.03328835	0.249850031	-1.82090457	0.008768379
MSTRG.120449.2	-0.019613549	0.733306115	1.118568806	0.026931498	-0.08778962	0.360929555	-0.05392024	0.100868125	0.147770035	0.302007651
MSTRG.120681.2	-1.984990668	0.412807597	2.695507149	0.286603091	1.664771696	0.695146472	-1.43674032	0.23460323	-3.531514776	0.004757317
MSTRG.121753.1	-0.099543425	0.115009667	0.257010137	0.049535219	0.078842355	0.698337727	-0.0225226	0.566988467	-1.143884221	0.014554963

MSTRG.121887.2	0.703324219	0.029805387	1.882736707	0.000345352	-0.40987993	0.584363586	0.023235007	0.960976807	-0.001268387	0.994634537
MSTRG.122249.3	1.114746595	0.001131357	2.363816225	0.001124355	2.284928635	0.109173105	2.025346184	0.000247631	1.552579588	0.00270486
MSTRG.122776.1	0.050492423	0.414417806	-0.31656197	0.122864589	-0.33678629	0.031053176	-0.07222114	0.166563425	1.310544905	0.000609645
MSTRG.122935.1	0.67460528	0.06313242	-0.05529293	0.86253056	1.28289128	0.231017731	1.003065613	0.004073778	0.773226762	0.087935673
MSTRG.124386.2	-0.050264206	0.574023691	-0.37158561	0.20403314	-0.14303065	0.554256212	0.140686266	0.057771312	-1.256212133	0.026883176
MSTRG.12474.1	0.195193372	0.014545576	0.732974373	0.052624706	0.289083915	0.213901716	-0.04795203	0.110153622	-1.16904324	0.008044426
MSTRG.125163.1	0.649738126	0.052727973	1.208047747	0.006715721	0.896278717	0.071510268	0.304733072	0.016551924	0.299236013	0.333885249
MSTRG.125329.1	-0.410974334	0.024810616	-0.47397989	0.170664742	-0.27042508	0.401376019	-0.40101761	0.001989782	-1.03364535	0.024513982
MSTRG.125358.1	0.562333919	0.032089345	-0.30923317	0.726073765	-3.61096347	0.096143504	-1.07822323	0.003297324	-0.63582184	0.240479704
MSTRG.125772.1	0.050234034	0.595874733	-0.17066219	0.371084114	-0.07132651	0.747112221	-0.12467334	0.049587326	-1.027563024	0.020697103
MSTRG.129775.4	-0.480825596	0.056085761	1.495791741	0.103792303	3.289018282	0.01884604	-	-	0.455883475	0.417197613
MSTRG.129890.1	-0.05172495	0.575745674	-0.31182506	0.12616751	0.238081916	0.07573298	-0.03982592	0.719084451	1.191167441	0.014678853
MSTRG.13087.1	-0.356180773	0.681096715	-1.78321764	0.000855989	4.630437858	0.053479091	1.308136191	0.219368453	0.033723731	0.979587417
MSTRG.131507.1	0.202817188	0.352754241	-0.27925488	0.180586386	0.155941979	0.281877619	0.275264294	0.122594138	1.319213655	0.008073566
MSTRG.132298.2	0.094841312	0.618441695	-0.28915859	0.75024962	1.494831191	0.022372564	0.146886119	0.14103595	-0.137039924	0.652107217
MSTRG.132302.1	-0.406169877	0.010060637	-0.81219313	0.0288505	-0.94390929	0.092859024	-0.15758665	0.701848937	-1.621681146	0.009751143
MSTRG.132403.1	0.309796813	0.158057919	0.519631217	0.155790738	1.215835151	0.419471251	1.070639224	0.0001654	0.468057757	0.416393236
MSTRG.132425.2	-1.046955718	0.003981323	-1.21099753	0.140009931	-2.5673222	0.261569383	-1.89621486	0.001171413	-1.249410505	0.157658614
MSTRG.132865.1	0.437359037	0.004047867	0.67725072	0.067732186	1.079590957	0.069429884	0.278675043	0.047327292	1.200637554	0.018009839
MSTRG.13379.1	-0.166082073	0.736109196	-3.11786856	0.000352835	1.56091124	0.60117609	1.964089103	1.53157E-05	0.33478021	0.854803628
MSTRG.134173.1	-0.086706154	0.335544425	1.225321994	0.003707708	-	-	-0.02283981	0.789475087	-0.147833797	0.606388925
MSTRG.135106.1	-3.614513575	0.002557043	2.09084388	0.002089515	-0.91582307	0.744242713	-2.81776274	0.020313062	-1.202000387	0.052361128
MSTRG.135346.1	0.054071126	0.373534467	0.581222563	0.108140316	-0.54180445	0.024906836	0.126362068	0.037659081	1.034010033	0.002343005
MSTRG.135659.1	0.048342166	0.434332929	-0.67982211	0.167450209	-3.51281008	0.000648387	-0.70930295	0.399232162	0.501464334	0.187444184
MSTRG.135726.1	0.328555996	0.04391315	-1.55546832	0.015104881	1.985183281	0.019487563	0.097117275	0.609641742	0.286238439	0.66098668
MSTRG.136219.1	1.45143063	0.011342228	0.765841027	0.015787763	-0.34516706	0.497123979	-0.16202964	0.067933928	1.15093895	0.011287125
MSTRG.136481.1	-0.05529691	0.890513902	0.605071341	0.017561472	3.312745256	0.182923933	1.498322031	0.000434292	-0.641213486	0.716156205
MSTRG.136954.1	0.089007359	0.065097224	-0.10557507	0.372362028	0.142261765	0.430865642	0.023920015	0.314998058	1.151950783	0.029068339
MSTRG.136961.1	0.87172122	0.006974084	0.264388013	0.073364835	-0.31507472	0.427332285	0.280338555	0.296717901	1.054447013	0.040404616
MSTRG.137084.1	-0.053269589	0.634114644	-0.94924309	0.041919547	-0.41538141	0.102407926	0.017169975	0.712866081	1.06048697	0.022037838
MSTRG.139355.2	-0.242402055	0.397744163	-1.5310173	0.012584827	0.913457886	0.653482989	0.855979766	0.030781725	-0.977365502	0.070162241
MSTRG.140367.1	0.330643382	0.120061501	1.480831705	0.002141523	0.945179582	0.028089277	0.33071185	0.186388094	-0.361382826	0.594674691
MSTRG.140426.1	-0.037939294	0.733046179	-1.29313795	0.001652872	-	-	0.057331002	0.218653479	0.084439336	0.866340846
MSTRG.140438.1	0.464241239	0.018547798	0.496285262	0.047982633	1.418142135	0.048451839	0.032727405	0.80854232	-0.61990649	0.113100576
MSTRG.140831.1	0.127259555	0.280793696	-0.98891844	0.010120547	-0.12998952	0.602456693	0.04611003	0.60065453	1.439652378	0.00048625
MSTRG.141247.1	0.032735183	0.617765706	-0.36529892	0.039273744	0.010379435	0.971773438	-1.79674769	0.031906388	0.273717617	0.760806639
MSTRG.141648.5	-0.923364214	0.000778475	-2.67233782	0.127071601	-0.49762814	0.533259873	-2.42402445	0.000720305	0.399548261	0.654417209
MSTRG.14168.1	-0.228423083	0.091065722	1.048188518	0.003730424	0.293214797	0.148984908	0.234205083	0.053791094	-0.487545503	0.063367007
MSTRG.142498.1	1.291448246	0.000505046	0.020871422	0.860029802	3.66292639	0.314722801	0.566714255	0.284747742	0.449089836	0.651894627
MSTRG.142569.1	0.169177026	0.025086315	-1.41023432	0.042027948	0.921194676	0.284826491	-0.37614123	0.430122486	-0.976760042	0.167338945
MSTRG.142849.1	0.05368775	0.071068875	0.704851868	0.010826674	-1.85190871	0.014784852	-0.1755222	0.131025182	0.278202069	0.058266491
MSTRG.142918.1	-0.08879487	0.11522699	1.176959438	0.028141136	0.404925076	0.112707766	-0.11046866	0.184519526	-0.080744876	0.452826598
MSTRG.143448.2	-0.013118401	0.572184958	-0.52406171	0.125414039	-1.5706161	0.131897815	-1.15254426	0.016365938	0.315495154	0.68115266
MSTRG.144025.1	0.160796336	0.303547013	0.174776923	0.070443986	-0.10979198	0.480360288	0.011426891	0.483636137	1.044628371	0.009060786
MSTRG.144045.1	0.138955588	0.12383108	-0.20141121	0.031654058	0.060298589	0.246867543	0.003170203	0.93310275	1.112136073	0.022340759
MSTRG.144536.3	0.824289155	0.349792275	1.331645284	0.000868593	1.851954402	0.212457747	0.961252184	0.042960566	0.366749278	0.53647563
MSTRG.144536.8	1.252616479	0.133452548	1.576248421	2.32429E-05	0.984248805	0.234542679	0.556653821	0.002729779	0.256897052	0.647251601
MSTRG.144890.1	0.064595682	0.703299342	-0.8576563	0.023877337	0.027067464	0.865151643	-0.10288557	0.387956827	1.024457891	0.024291752
MSTRG.144914.1	1.531793424	0.002874098	2.317162568	0.000141773	2.138228454	0.00332485	1.236287299	9.46118E-05	1.635573501	5.69253E-05
MSTRG.14651.1	0.053250096	0.519417569	-0.03582566	0.839836696	0.500788521	0.181545817	0.055011939	0.039515502	1.192702405	0.041326794
MSTRG.146953.3	0.616778591	0.241938803	1.201245141	0.000484181	1.912796078	0.267769045	0.490563644	0.101895122	1.152159045	0.123185689

MSTRG.147334.1	0.077033319	0.521593454	0.129256106	0.307005232	-0.21833267	0.134448502	-0.06976951	0.089765741	-1.131541987	0.014588263
MSTRG.147512.1	0.032196407	0.559842174	-0.14161174	0.548781353	0.242234701	0.064555766	-0.07894254	0.002114027	-1.088492621	0.042898977
MSTRG.148119.1	-0.073521268	0.101326872	-0.91669	0.001846889	-0.03890822	0.701790149	-0.15760848	0.041417422	1.026841645	0.022690627
MSTRG.148734.1	0.119416938	0.280296893	1.104746792	0.000766987	-	-	0.062811342	3.128E-05	-0.561241878	0.062208141
MSTRG.14927.5	-	-	-1.07136513	0.069952087	-1.7431749	0.034812019	-0.28588821	0.322452549	0.265476103	0.826470855
MSTRG.149637.1	-	-	-0.74671087	0.065536538	-0.59520698	0.216096719	-0.22080818	0.072505035	1.426298017	0.026910363
MSTRG.150300.1	0.014061276	0.830690816	0.218638802	0.139994559	-0.09232864	0.829490592	-0.05528839	0.018294844	-1.076723626	0.034483406
MSTRG.151770.1	0.230489165	0.289059763	-1.06352025	0.014929689	2.552598879	0.007350256	0.206362554	0.398740962	-0.340653803	0.303167045
MSTRG.151803.1	-0.008765105	0.933705367	0.079669247	0.091028331	0.040128746	0.665051426	-0.0937765	0.041699228	-1.531479922	0.034481198
MSTRG.152528.3	1.191806425	0.012493512	1.24346601	0.000445787	1.667797854	0.023705997	1.141889066	0.000799882	0.304545592	0.386798443
MSTRG.152528.4	0.157287153	0.709719407	0.277461251	0.594616674	1.203164579	0.043154852	1.136108928	0.000551755	0.229089148	0.545088869
MSTRG.152587.1	-1.12754703	0.002220212	-0.55484593	0.039748403	-0.72413604	0.155523835	-0.91360596	0.084708363	-1.226313891	0.058056569
MSTRG.152692.1	-0.244817818	0.06145005	3.904892438	0.002144319	-2.95862524	0.362470789	-1.34604446	0.091312463	0.676752529	0.514380498
MSTRG.153333.1	1.140921405	0.000369448	-0.21539469	0.424519656	3.804754789	0.027377131	1.369806964	0.000207049	0.115430629	0.313007743
MSTRG.154286.1	0.756284648	0.003246292	0.392159201	0.083732229	-0.01864495	0.982523806	1.011176134	0.000165089	0.849319261	0.042900622
MSTRG.155793.1	-0.009286779	0.609608392	0.651958846	0.217014856	-0.22128776	0.19358617	-0.00707991	0.798057339	-1.13878469	0.011974247
MSTRG.156420.1	0.483603558	0.102720841	1.613602581	0.000334806	-0.07679335	0.845458924	0.227013038	0.294120438	-0.042141771	0.910917137
MSTRG.156938.11	0.230275136	0.359587146	0.787834628	0.08584997	0.235966029	0.749719327	1.33745816	0.004996774	-1.369824337	0.106866575
MSTRG.157049.1	-1.421828872	0.294121719	1.264904139	0.003651531	-3.40613544	0.277495581	3.86593747	0.00053996	-3.955752444	0.276655704
MSTRG.157050.1	0.013042598	0.769628352	-0.63520957	0.004608705	-1.03148191	0.019953165	-0.22411038	0.086788894	0.593795761	0.177418697
MSTRG.157498.1	-0.214609093	0.158087846	-1.03407318	0.00170456	1.076286289	0.014836067	0.641141327	0.054404663	0.89976299	0.309872605
MSTRG.159471.1	0.212642273	0.091729969	-0.57384883	0.004245583	1.960241536	0.043352073	0.043924239	0.650092701	-0.863221515	0.293046988
MSTRG.159553.1	-0.53761929	9.26182E-05	-0.92629615	0.294289629	-1.06503973	0.000486481	-1.10860824	0.016435822	-0.286847227	0.567501275
MSTRG.160024.1	0.088320394	0.278488924	-0.30990929	0.21271297	-0.02173994	0.86663446	0.040105325	0.312630468	-1.635165645	0.015593899
MSTRG.160092.1	-1.094455109	0.007515433	-0.32622867	0.11847059	-1.01450472	0.007743838	-0.22301255	0.020385154	-1.609017082	0.005562585
MSTRG.160193.1	0.033846464	0.597734126	-0.03907687	0.754272496	-0.16996744	0.11570292	-0.17608597	0.109881792	-1.150731174	0.000562361
MSTRG.160637.1	1.478570662	0.070975699	0.404743619	0.612587047	-1.04946298	0.029063533	0.08919404	0.718759972	0.032268819	0.922253634
MSTRG.161579.1	1.497621115	0.032755262	-0.38039133	0.870224836	0.958621117	0.781966196	0.869279567	0.454712921	0.099576224	0.925491361
MSTRG.165141.1	1.253123312	5.67284E-05	-0.06680438	0.599556037	0.521532603	0.251251531	0.479428666	0.019083706	1.642296294	0.093513437
MSTRG.165253.1	0.052090532	0.610929574	0.16493047	0.085849861	-0.14207666	0.174210526	0.174593294	0.065001529	1.137137338	0.006681496
MSTRG.165614.1	0.032219464	0.85210944	1.117863611	0.000509943	-0.38430862	0.360418964	0.148052472	0.041252588	0.266381186	0.143770688
MSTRG.165708.2	-0.268588684	0.12489927	-0.22744815	0.284780628	-1.12441797	0.082519413	-1.03930681	2.46756E-05	1.097498515	0.164859607
MSTRG.166194.1	-0.096332047	0.203848568	-0.84450178	0.040427351	0.45192727	0.243877979	0.442557581	0.010231739	-1.022285466	0.006348714
MSTRG.16673.1	0.074043794	0.57878433	-0.13611735	0.784784569	0.094750287	0.389117049	0.14738152	0.186156159	1.45113321	0.018979046
MSTRG.166955.1	-0.341285217	0.009538161	0.037989809	0.528094096	0.22256603	0.093771411	0.070106491	0.327578482	-1.159039812	0.001830542
MSTRG.167497.1	-0.345173852	0.005226032	0.336611601	0.016355509	-0.11663451	0.330777214	-0.01037101	0.264068361	-1.186032592	0.048581845
MSTRG.167514.1	-0.158780771	0.000265723	-0.05992962	0.847731258	-0.40126491	0.103811716	0.104499573	0.248355063	-1.127574126	0.011200201
MSTRG.168063.1	0.333172573	0.019913485	0.395489431	0.015574082	1.027806424	0.034139443	0.220040589	0.107891316	-0.84894506	0.034163287
MSTRG.16888.1	0.15634457	0.065940324	1.340024969	0.010494781	0.723664632	0.181987765	0.071610913	0.811777944	-0.608030058	0.009575006
MSTRG.168917.1	0.056157779	0.181856561	-1.05623453	0.034129427	0.064670674	0.000121307	-0.01869412	0.504560038	-0.969263026	0.12484386
MSTRG.169137.1	-2.330936739	0.003772085	-0.0083358	0.576812976	0.008361777	0.889842021	-0.01300021	0.16707767	-4.377201286	0.094344229
MSTRG.169142.1	-0.506836659	0.005249716	-0.9698196	0.006157293	-0.31493955	0.424147065	-0.53724281	0.002659471	-1.25126014	3.38686E-05
MSTRG.169297.1	-0.03989374	0.579612001	-0.76597918	0.020701647	0.525686556	0.007011593	-0.06055319	0.003395576	1.535080423	0.010156554
MSTRG.170254.1	0.252619703	0.113355234	0.126711744	0.215315663	0.631715192	0.138281839	0.407085547	0.005234824	1.245887667	0.005936103
MSTRG.170713.1	-0.299140318	0.015109276	-0.66655981	0.088337284	-1.13054431	0.025939312	-0.32442032	0.031348477	-0.108354044	0.625610631
MSTRG.171001.1	1.669090878	0.017123239	0.97891096	0.032202901	0.277408784	0.790619608	0.335710629	0.044546019	0.589978437	0.094632182
MSTRG.171516.4	-0.615986303	0.068534867	1.177888272	0.028902575	0.80476951	0.384889944	-0.03147825	0.928124613	0.182316067	0.540217916
MSTRG.171689.1	-	-	-1.68357554	0.037911921	-2.59519023	0.026881319	-	-	0.000977319	0.694773732
MSTRG.172078.1	0.060807155	0.437190596	1.098394339	0.000782675	-0.02087278	0.940455792	0.123026208	0.023173518	-0.418199988	0.001486138
MSTRG.17252.4	-0.264234582	0.709501908	0.768159525	0.111350902	0.108571898	0.897210992	1.254167216	0.038632469	0.128534349	0.618429876
MSTRG.172847.1	0.251362171	0.386662672	1.213934524	0.008928478	0.825003937	0.228885796	0.586698875	0.024996331	-0.872265463	0.208934819

MSTRG.173240.1	0.082542527	0.405361952	0.203963953	0.280070178	-0.24181602	0.246713682	0.129230617	0.117183237	-1.238557992	0.001287203
MSTRG.173283.2	0.37056221	0.085563184	1.254074435	0.006180921	1.422409185	0.304167785	1.103291459	0.005251252	0.61230105	0.204777157
MSTRG.173283.4	-0.109762381	0.485947509	1.356452293	0.043229866	0.963699467	0.3822685	0.888314466	0.001727205	0.294450503	0.523555975
MSTRG.173797.2	0.910019351	0.044459716	2.21690404	0.0095332	2.252327213	0.321271903	0.96211274	0.095910889	0.034324313	0.976263482
MSTRG.174719.1	0.056217954	0.498635636	0.251863575	0.237784983	-0.14598129	0.555407591	0.056475657	0.288968876	1.200678027	0.002797071
MSTRG.174897.1	0.850720821	0.011797844	-0.08620466	0.794930307	-1.79645788	0.536625322	1.292487506	4.17225E-05	-0.418043899	0.096965163
MSTRG.175157.1	-0.063899872	0.073845923	-1.07483094	0.006014929	0.131732418	0.714312032	0.164139681	0.021029528	0.582692037	0.078408053
MSTRG.177973.1	0.007771951	0.971052579	0.706375397	0.037094617	-0.14782443	0.623030412	-0.23933127	0.301624323	-1.460960009	0.000559406
MSTRG.18848.1	0.068323184	0.550544393	2.92086287	0.000261638	-1.49692308	0.205981788	-0.58386728	0.002068788	0.539325431	0.094390989
MSTRG.20854.1	0.032505794	0.188961719	0.351508933	0.290907303	0.119020828	0.578773074	0.035960505	0.179072442	1.238084748	0.046217884
MSTRG.21024.2	0.029758399	0.764250995	-1.07977889	0.211120343	0.1638748	0.395151692	0.068342538	0.145328487	-1.495691013	0.004313775
MSTRG.21076.1	0.206750596	0.056840919	0.761296057	0.001695372	0.939511974	0.263622605	1.25961582	0.000646969	-0.40813051	0.402410332
MSTRG.21128.1	-0.269579504	0.000141122	-0.05269498	0.013237974	-	-	-0.02136908	0.342486503	-1.218283366	0.001925522
MSTRG.21556.1	0.14149246	0.053892328	1.047431684	0.012550768	0.088322831	0.001829467	0.111042891	0.000593487	0.24128152	0.098582324
MSTRG.21586.1	0.222648328	0.327398584	0.544953501	0.002865102	-	-	0.021879314	0.633081728	-1.30849569	0.022554167
MSTRG.22142.2	-0.022611579	0.910858176	-1.02720046	0.615180914	0.207297189	0.838317687	0.347410043	0.182355804	3.195135657	0.02954076
MSTRG.22166.1	0.31752276	0.039987997	0.55115578	0.252222439	2.202181154	0.005002808	0.542461984	0.115911167	0.11306119	0.778686008
MSTRG.22226.1	-1.530984309	0.000881886	-0.78946673	0.032055589	0.253486504	0.687707627	-1.27069191	0.007755868	-2.062027649	0.002172021
MSTRG.22570.1	-0.018658237	0.459077124	-1.14536509	0.017570823	0.20247025	0.380413782	0.034253985	0.583319894	-0.367508574	0.170347829
MSTRG.22685.1	0.060098628	0.448743743	0.061979109	0.722656861	-0.16047261	0.382311183	-0.25985817	0.05960775	-1.484121801	0.001996493
MSTRG.24321.16	0.06518963	0.41170535	0.038527256	0.896620222	-0.35588453	0.392170015	-0.0613368	0.743302413	1.422416378	0.040055095
MSTRG.24322.3	-0.300790542	0.200023746	-1.17542725	0.005683359	-0.43511956	0.071256644	-0.44646746	0.055779573	0.242838054	0.684568748
MSTRG.2509.2	1.980218638	0.293534682	0.034995552	0.9426463	-7.23269277	0.141122859	2.010890656	0.215420608	5.928970685	0.04500218
MSTRG.2510.1	1.13106654	0.000154215	0.52549534	0.078531614	0.326473021	0.265956055	1.40614427	0.000274699	1.708066197	5.23574E-06
MSTRG.25168.1	0.10514596	0.405839702	-0.04157328	0.807916119	0.634736787	0.194966065	0.10209749	0.169403853	1.106944094	1.20293E-05
MSTRG.2520.1	-0.587035704	0.000785234	-1.43783704	0.025913939	-0.78519142	0.00367788	-0.85378965	0.166841637	0.312672664	0.596932537
MSTRG.25940.1	0.00311385	0.957888439	-0.21534564	0.44710112	-0.21027531	0.109252917	-0.10909014	0.235124342	1.081929743	0.014049267
MSTRG.26671.1	0.094793094	0.482377991	0.522703693	0.033374176	0.340219061	0.021356764	-0.10361396	0.334729252	-1.135700154	0.000569165
MSTRG.27034.1	-0.095717591	0.234249702	0.50529262	0.044037475	-0.1211198	0.679380287	0.067775255	0.103213217	-1.014981511	0.008403393
MSTRG.27387.1	1.055121764	0.001819085	1.054978388	0.002414448	1.922618754	0.128877072	1.108033217	0.003037169	0.678155106	0.004912482
MSTRG.27430.1	-0.181867746	0.214748376	-0.93751861	0.100271201	-1.02100707	0.005691759	-1.16308966	0.013662091	0.49076661	0.587705649
MSTRG.27430.2	-0.861791761	0.010879535	-1.99942583	0.000383375	-1.24179885	0.00942084	-2.18343448	8.62291E-06	0.685645873	0.637552044
MSTRG.28486.1	-0.098826403	0.293841616	-0.03769427	0.801275721	0.069144298	0.528603836	0.029625123	0.218794803	-1.00262141	0.024694314
MSTRG.29440.1	-0.036532678	0.727710023	-0.0340654	0.594590213	-0.10849857	0.054015111	-0.18545743	0.040786525	-1.139359568	0.000281584
MSTRG.30096.1	-0.060641027	0.414129001	-0.05489434	0.838062323	0.191710065	0.190360507	-0.07091823	0.167793844	1.182409505	0.039636349
MSTRG.30660.1	-0.143780768	0.524341298	-0.50138174	0.261312693	0.116689686	0.355041904	0.079757657	0.532605084	1.004885588	0.025340997
MSTRG.3134.1	0.286130232	0.218882168	0.801241397	0.13531776	1.244842932	0.008025546	0.121925286	0.630286086	-0.334127328	0.365864662
MSTRG.3161.3	0.145969741	0.71484052	-0.13583701	0.425914257	2.350515895	0.075609543	1.102085925	0.007132172	-1.701404608	0.267643542
MSTRG.31771.1	0.137124848	0.136147108	0.316137771	0.009139944	-0.06213775	0.734574772	0.15445638	0.01861114	1.014600328	0.005258904
MSTRG.32003.1	-0.198437046	0.14849259	0.447113242	0.091553953	-0.28178431	0.062312108	-0.03107068	0.173544997	-1.39585993	0.00015147
MSTRG.32294.1	0.00357468	0.85532141	1.095419582	0.012977781	0.135701234	0.320640416	-0.06328644	0.514765774	1.278838107	0.001580651
MSTRG.34095.1	-0.068533884	0.427902308	0.009637516	0.929413756	-0.57593444	0.007454803	0.015802807	0.515339609	-1.00246676	0.019687577
MSTRG.34278.2	0.065811212	0.589571129	-	-	-0.22249262	0.057187874	-0.09815458	0.102723054	-1.320598304	0.01122198
MSTRG.34360.1	-0.199818514	0.571625738	-0.41453482	0.010105431	-0.08112167	0.213812296	-0.07266488	0.083388702	-1.796746146	0.00306482
MSTRG.34441.1	-0.074561536	0.473476212	0.663711063	0.003973274	0.220502171	0.02689404	-0.14499737	0.167455707	1.179998958	0.001180974
MSTRG.34462.8	0.046631988	0.542383062	0.271004127	0.047015926	-0.00268848	0.87753953	-0.18045373	0.735183362	-1.832141462	0.009291489
MSTRG.34464.1	-0.11552943	0.315918352	-0.1753183	0.299047864	-2.08367216	0.000152096	-0.97118064	0.001194119	-0.24492691	0.299682796
MSTRG.3507.1	-0.338322643	0.066750004	0.590565063	0.00345365	0.23470101	0.110102395	-0.11043556	0.010787471	-1.000440777	0.007090052
MSTRG.3508.1	-0.170867105	0.122322027	0.019825108	0.880154584	-0.10138409	0.290766027	-0.03401766	0.0309017	-1.334089947	0.033901759
MSTRG.35970.1	0.050589018	0.504691394	-0.55874947	0.004888238	-0.03733974	0.85057852	0.184898103	0.017452574	1.499072643	0.018378798
MSTRG.36126.1	0.87786432	0.097496378	-1.27204079	0.091819309	1.164098661	0.697711838	1.111071912	0.029468665	-2.497110343	0.010734853

MSTRG.37049.1	0.02448715	0.86990765	0.883671533	0.016105795	0.011716618	0.962325153	0.075038491	0.358049972	-1.386597597	0.04711345
MSTRG.37463.1	-1.094547057	0.096924486	-0.24318899	0.498542458	0.371879526	0.618853461	-1.33665202	0.045063725	-0.098337291	0.942663657
MSTRG.38318.1	0.735036583	0.047094756	-0.10118414	0.636159615	-1.10083801	0.146461146	-1.10560679	0.01009235	-0.334966051	0.311461323
MSTRG.38815.2	0.140812615	0.396612111	0.657471049	0.11399176	1.704392096	0.04914496	0.897301658	0.369454603	-0.555484038	0.751512287
MSTRG.109897.16	0.636171853	0.00246965	1.403312801	0.023369732	1.066489187	0.023718376	0.856416594	0.004356333	0.707636856	0.231840044
MSTRG.40823.1	-0.032851414	0.57557007	-0.70215672	0.007627265	0.102071607	0.750392542	0.069999411	0.635616679	1.116884523	0.020604199
MSTRG.4109.2	-0.0052925	0.95820806	-0.24089294	0.169437357	-0.28169441	0.25355951	0.157536495	0.112261719	-1.161678145	0.020419271
MSTRG.41494.1	-0.494975326	0.339322423	-1.41041321	0.000165394	-1.38269152	0.169503699	-0.39321915	0.010548153	-0.866247112	0.001265954
MSTRG.41494.2	-0.481929052	0.512000122	0.607581589	0.005720263	1.310494469	0.027513979	-	-	1.397117907	0.021128322
MSTRG.41603.2	-2.069628713	0.009357232	-1.50791914	0.013949882	-0.92513405	0.126166575	-1.95751069	0.003593094	-2.4305164	0.013225048
MSTRG.41897.1	0.018598154	0.698061983	0.132204756	0.084198392	0.09689114	0.129821397	-0.10471379	0.000885479	-1.0558264	0.013640177
MSTRG.42618.1	-0.021190858	0.829679809	0.931559331	0.04944746	-	-	-0.2487556	0.031967797	-1.119060616	0.000403949
MSTRG.43488.1	0.043784521	0.595277403	0.019810232	0.780886042	-	-	0.005264036	0.912922847	1.059099596	0.000962897
MSTRG.44144.1	-0.073320106	0.207395931	0.056566992	0.435340982	-	-	-0.15131051	0.002549876	-1.035534572	0.0001198
MSTRG.44148.1	-0.071198867	0.035484973	-	-	-0.32833203	0.0562643	-0.34412684	0.034563335	-1.668667005	0.001194459
MSTRG.44175.1	0.145369372	0.212020049	-0.6720348	0.0262275	-0.3142474	0.365432432	-0.15497326	0.116208003	1.633511196	0.001128599
MSTRG.45430.1	-0.277305241	0.005859838	-0.3833321	0.016692843	-0.41034181	0.579210789	-1.07790606	0.001257445	-0.31222135	0.193553309
MSTRG.46271.2	0.051653394	0.486116702	1.021495518	0.001554782	-	-	-0.05487923	0.203503211	0.014139815	0.858121031
MSTRG.47082.1	0.04155729	0.337796443	0.052627033	0.839567289	-0.40186623	0.065982265	-0.20487876	0.018062904	-1.080680455	0.005486168
MSTRG.47338.2	-0.30629933	0.013760027	0.186795362	0.436646158	0.267326281	0.222116466	0.131484336	0.047621646	-1.109828213	0.00325032
MSTRG.47412.1	0.059299138	0.253918488	0.162184067	0.734040819	1.201307011	0.048290764	0.505695598	0.097512175	-0.400108356	0.50848713
MSTRG.47657.1	-0.069074811	0.203615051	-0.01248189	0.915536628	-0.21926266	0.005985294	-0.05593442	0.207328544	-1.396099027	0.00508681
MSTRG.48337.3	-0.291671173	0.223575337	-1.24538007	0.017790309	-	-	-	-	-0.975252222	0.006101505
MSTRG.50904.1	0.074232473	0.027249231	-1.31424251	0.003800034	-0.09761057	0.830936401	0.363139316	0.012564431	0.52310016	0.034638245
MSTRG.51561.8	-0.800963032	0.014400357	-0.91468187	0.030411685	-1.37981037	0.039219153	-0.64052598	0.01036905	-0.634184607	0.001125196
MSTRG.52129.1	0.337769621	0.423861041	-0.17645493	0.172632926	-3.68080527	0.006796629	-1.51020396	0.002078439	0.060653675	0.103918667
MSTRG.5441.1	0.014184552	0.880010493	0.018019003	0.960820341	0.200815308	0.008966544	-0.03544511	0.560750218	1.051745732	0.013904367
MSTRG.55875.1	1.356945009	0.002560268	1.174196384	0.002429825	1.753165687	0.039016683	1.570202112	1.52806E-05	1.355211444	0.003762136
MSTRG.56429.1	0.054931706	0.476355221	1.050927184	0.008717469	-0.11124158	0.554042593	-0.02885262	0.6448077	0.459471895	0.344732722
MSTRG.56476.1	0.225765742	0.054978394	-0.17722519	0.48685395	-0.61530695	0.084307606	-0.06316067	0.661989301	1.079409212	0.009499595
MSTRG.59243.1	0.174492999	0.141299847	0.403043029	0.116860198	0.372951424	0.109446362	0.083238061	0.309911438	-1.300023511	0.002466606
MSTRG.59305.1	0.12893598	0.290836373	-0.13771673	0.40658062	-0.25078327	0.567662549	-0.02455985	0.739062781	-1.176133969	0.002097301
MSTRG.60061.1	0.413549284	0.122858591	1.506303191	0.000424363	0.783856911	0.001846364	0.442156254	0.009598823	0.311884905	0.094988048
MSTRG.61575.1	-0.328820229	0.009773377	1.164274886	0.001505388	-0.30738	0.080750516	-0.00108728	0.982507238	-1.230076476	0.045076686
MSTRG.61812.1	0.32230277	0.309537271	1.306342583	0.023820812	0.143691224	0.92874277	0.032233088	0.927764573	-0.152960739	0.562859221
MSTRG.64623.1	0.530641345	0.007481354	1.280430447	0.007263969	2.721783529	0.004919633	1.386470592	0.031034026	-0.980222214	0.075349766
MSTRG.64871.1	0.118231814	0.156232637	-1.38931404	0.019493978	0.092809178	0.204160849	0.067297159	0.091061187	-0.046520094	0.706479026
MSTRG.6496.1	1.063031268	0.007645139	0.380042612	0.380687975	-0.04256108	0.943857501	0.143790435	0.578318766	0.905820528	0.201862075
MSTRG.65438.1	0.044673659	0.632566055	0.118164357	0.287803574	-1.80118505	0.000505609	-0.35083227	0.448857299	0.254680953	0.191576057
MSTRG.67339.1	-0.131493174	0.053242146	-1.14851297	0.033264649	-0.10010052	0.882365834	-1.73492515	0.000990376	0.645474895	0.484581242
MSTRG.67732.1	0.09902116	0.07707796	0.051518241	0.540570737	0.067170938	0.608764328	-0.15086241	0.075363095	-1.002613507	0.03210042
MSTRG.67827.1	0.084901267	0.505604215	0.570232883	0.023094008	-0.02505213	0.852858679	-0.05564761	0.218509262	-1.048526148	0.004711728
MSTRG.67831.1	0.098679286	0.196092417	0.517213224	0.001579124	0.106844814	0.645572651	0.026519609	0.443285755	-1.097256319	0.014904928
MSTRG.68709.1	-0.000218211	0.99766866	-0.73687908	0.042370407	1.056326164	0.003915979	0.021078974	0.930786574	-0.494723337	0.295803199
MSTRG.69290.1	-0.404288441	0.053546721	-0.49778987	0.115401969	1.147262648	0.0395064	-0.06712426	0.743070642	-0.70775273	0.055768308
MSTRG.71506.1	0.729575948	0.209159254	0.887433757	0.04419648	-0.67868968	0.297568406	0.64776272	0.001453557	1.072993121	0.008131199
MSTRG.71734.1	-1.124509457	0.000137671	-0.07756803	0.296401949	-0.17921747	0.081546913	0.016751704	0.621821574	-1.666574624	0.137439453
MSTRG.72339.1	-0.531111225	0.148299399	-0.06454925	0.714532681	-0.46119352	0.047454673	-0.6194613	0.070573304	-1.332957331	0.005356542
MSTRG.72701.1	1.746290505	0.002629934	-0.46084974	0.131759871	0.543575116	0.213924866	-0.47465534	0.10768671	1.932058435	0.014646558
MSTRG.72988.1	0.494569531	0.101355581	-0.07018361	0.933111284	0.796108675	0.467852645	1.040446189	0.000136229	0.914467243	0.144141896
MSTRG.73145.1	-1.336213152	0.00059247	-0.99240977	0.060708365	0.424700773	0.207817527	0.912574748	0.000487815	-0.123337911	0.686847081

MSTRG.73230.1	0.146937824	0.377575883	1.259185522	0.020937406	0.00616989	0.955405803	-0.00189831	0.985811748	-0.60031501	0.138699375
MSTRG.7323.1	0.206810082	0.044203217	0.2646518	0.133240477	-0.09244602	0.602520897	-0.25792952	0.109680585	-1.526327555	0.035899688
MSTRG.73597.1	-0.769927948	0.000411891	-0.21802624	0.312623706	-0.00088517	0.995465924	-0.20732161	0.027680838	-1.107726662	5.99923E-05
MSTRG.7445.1	0.260767697	0.003230787	0.684965571	0.025000795	1.571246949	0.009372389	0.650152838	0.017484494	-0.754476193	0.406491807
MSTRG.75332.1	0.381692682	0.088844853	1.21277801	0.006478052	-1.52249757	0.137898853	-0.53554565	0.183239058	-0.20427134	0.213565293
MSTRG.76070.1	3.41216435	3.00009E-05	3.152143405	0.000379466	0.406901819	0.761907984	2.171363758	0.00031602	1.841896283	0.1552237
MSTRG.76083.2	0.80600388	0.473004927	-1.20321172	0.606260875	-0.27937896	0.919270802	2.259576197	0.00260482	0.691835635	0.028895986
MSTRG.76158.1	-1.59695263	0.001751622	0.385470154	0.055209368	-0.37021547	0.487542855	-0.13704169	0.306823241	-0.390978701	0.012494806
MSTRG.76260.2	1.58253987	0.062464668	0.666930838	0.02280398	-1.33918096	0.03274999	-	-	-	-
MSTRG.76841.1	0.007247261	0.909405393	-0.22689303	0.332754563	0.214199265	0.21148931	-0.03089957	0.717899697	1.153653561	0.019652079
MSTRG.77408.66	1.232864751	0.031073292	0.560103499	0.004801043	1.143247493	0.098856547	0.717855247	0.000461497	1.178587943	0.023671234
MSTRG.78722.1	0.169592446	0.348059951	-1.05603288	0.011311024	0.269431045	0.172101607	-0.0583563	0.116625912	0.298944224	0.31706141
MSTRG.78767.1	0.057010242	0.563511992	0.004517151	0.965423803	-0.00229755	0.974552017	0.025939229	0.819450098	1.089901142	0.017871517
MSTRG.78823.1	-0.214387372	0.007740943	0.010687833	0.852251052	-0.10440772	0.236091146	0.052224719	0.402313343	-1.019330869	0.003920394
MSTRG.78833.1	-0.006934249	0.93088112	-0.12508785	0.127498932	-0.20404411	0.045965938	-0.16843114	0.000587756	-1.147072124	0.009674162
MSTRG.78949.1	0.012588639	0.824869306	0.207230797	0.022387578	0.192292067	0.130406815	-0.03690359	0.34329309	-1.061605759	0.002946397
MSTRG.80048.6	-0.264598225	0.173895503	-0.44734574	0.766669284	0.064560132	0.369205657	-	-	-1.414607413	6.16892E-06
MSTRG.80051.1	0.159362293	0.420518694	-1.27840677	0.033546702	0.097569158	0.695468288	-0.03804524	0.482472851	-	-
MSTRG.80090.1	2.615600725	0.019449051	-4.68418076	0.077707516	5.672202343	0.102059547	0.653779937	0.54390587	1.964449182	0.022859251
MSTRG.80090.2	0.201486402	0.509127672	5.22952784	0.037160484	-5.19380017	0.063602822	0.432946899	0.615799047	1.080495533	0.156586305
MSTRG.80437.1	-0.031042942	0.822933418	0.106138758	0.230895297	-0.18488003	0.170172592	0.201195697	0.0141107	-1.046241022	0.036097525
MSTRG.80798.1	0.110206518	0.115600186	0.177470942	0.03043079	-1.44207061	0.01468911	-0.37034171	0.064886383	-0.169125953	0.62014523
MSTRG.81445.1	-0.197531147	0.056216318	0.252373752	0.290686156	0.565448894	0.13847067	-0.23916907	0.013565489	-1.493470307	0.002369815
MSTRG.820.6	0.449850748	0.000811918	1.671926863	0.017444593	1.289754541	0.078882865	0.697167502	0.00380255	-1.942400945	0.139352631
MSTRG.82486.2	-1.572887883	0.005831332	-	-	-	-	-	-	-3.433413775	0.08739003
MSTRG.82517.1	-0.784902335	0.000225071	-1.26315316	0.011387089	-0.78351928	0.022170061	-0.38813717	0.072643517	-0.627950211	0.096249198
MSTRG.85040.3	1.098726753	0.03250901	-0.75120674	0.413386699	0.976098889	0.258338636	-	-	0.423985013	0.392260425
MSTRG.85226.6	-	-	1.28519865	0.0100858	-	-	0.26845924	0.210901405	0.325672759	0.6005453
MSTRG.85473.1	0.403749584	0.151836489	0.86390877	0.055556538	1.421019371	0.024306029	0.600445394	0.07540202	-0.791992894	0.002832671
MSTRG.85791.1	0.085010738	0.157487428	0.364770298	0.036103325	-0.07927977	0.48196153	0.134049115	0.006506943	1.020342794	0.033161531
MSTRG.87036.1	-3.048829696	0.003878308	-0.71427117	0.373061905	-0.39112689	0.836598845	-2.91211819	0.01616206	0.273251177	0.399682261
MSTRG.87036.3	-2.054735212	0.013764573	-1.63860999	0.582298824	4.854381721	0.394616206	-4.31179709	0.105568533	-0.446306805	0.485636678
MSTRG.87209.1	-0.263708233	0.034631185	-0.07457743	0.723677866	-1.51611326	0.002573683	-0.93236089	5.54988E-05	0.300439457	0.240049985
MSTRG.88522.1	0.079644676	0.228068571	-0.61788441	0.006956306	-0.02060994	0.918228006	0.019118423	0.652860273	1.154662894	0.018732801
MSTRG.89166.1	0.714486751	0.026000901	1.671528435	0.002668533	1.440822905	0.037191305	0.310880091	0.019640691	0.617749848	0.169722167
MSTRG.89632.2	-0.020960999	0.734407074	0.67447262	0.355618807	0.006322324	0.297945902	-0.08478311	0.347363674	-1.236280985	0.000538575
MSTRG.89670.1	-	-	-1.35676511	0.092671004	-2.09036078	0.022966385	-	-	-0.06681485	0.100870184
MSTRG.90641.1	0.036664795	0.656580241	0.010876853	0.725001229	-0.15110676	0.885404426	0.473313671	0.015901406	-1.014068988	0.004319361
MSTRG.90734.1	2.373266306	0.010139927	-0.3015727	0.03048036	0.934234225	0.055694815	-1.14867826	0.057951196	2.391222301	0.181879862
MSTRG.90734.2	-1.231137811	0.007762381	-1.69494657	0.126074343	-3.95735561	0.007008667	-	-	-1.82980993	0.086929147
MSTRG.91256.2	2.075236551	0.000174313	0.118091446	0.470931171	-0.11420356	0.920637465	-1.34003834	0.207468825	1.880272256	0.226767533
MSTRG.91539.1	-0.840086185	0.000112077	-1.99228566	8.45233E-05	-1.02602874	0.082553592	-0.00605854	0.744772146	-0.096637544	0.300153629
MSTRG.91921.1	0.31361545	0.020197923	0.317892645	0.048495704	1.083587068	0.009682249	0.08231497	0.631378503	1.122671034	0.047292976
MSTRG.92737.1	0.067789953	0.811350384	1.085793442	0.016207854	0.210548264	0.298151491	0.090318773	0.258343079	-0.074457512	0.629696078
MSTRG.93970.1	-0.065857136	0.450621775	-0.09904043	0.442889172	-0.02858516	0.640204811	-0.05047224	0.149188317	1.118715773	0.026582818
MSTRG.9417.1	0.067850549	0.229760645	-0.14171593	0.278575605	-0.03436858	0.674971247	0.115704054	0.00475304	1.067962815	0.004129472
MSTRG.94202.1	0.085012506	0.505537458	-0.07397634	0.736714136	0.344658934	0.014067523	-0.01585326	0.633013463	1.463563712	0.004967873
MSTRG.94539.7	-0.565071097	0.130635771	-0.84498091	0.100604413	1.05355862	0.115840665	-0.43824608	0.051999308	-1.213462108	0.032698706
MSTRG.94716.1	-0.205635043	0.183358905	0.725665127	0.018647339	0.707601396	0.016361424	0.17275704	0.03420338	1.218012877	0.010034277
MSTRG.95644.9	0.703069914	0.020007325	1.050630033	0.003014973	1.647428674	0.26696524	0.732771167	0.00113564	-0.677483385	0.278757388
MSTRG.95784.1	-0.425064381	0.003939983	0.576881552	0.000290852	0.167940529	0.227587704	-	-	1.060166331	0.001982291

MSTRG.95933.1	0.032490673	0.126559958	0.345983077	0.033302981	0.081914588	0.499102263	0.109765006	0.102805433	1.301575706	0.002068439
MSTRG.96255.1	0.008286376	0.928556765	-1.09619694	0.013468958	-0.11381166	0.552344448	0.033564199	0.748031315	0.036449427	0.863357395
MSTRG.96292.1	0.09894246	0.393337599	-0.23904085	0.149997543	0.175904529	0.223535696	-0.00114079	0.987094144	1.127722684	0.013340857
MSTRG.96942.1	0.171378491	0.310494298	1.006037718	0.08157417	0.159600276	0.099213505	-0.06804173	0.633383073	-1.321823251	0.000910369
MSTRG.9746.1	0.204129904	0.088838477	-1.05372622	0.011975084	2.314788713	0.005369285	0.529037412	0.004766494	0.546695671	0.164954172
MSTRG.97720.1	1.030667825	0.002443203	1.404162385	0.00045819	1.197569835	0.021583972	1.213118937	8.22228E-06	0.592326012	0.164701374
MSTRG.98044.1	-	-	0.216121641	0.85316916	-	-	-	-	-1.712037785	0.000954333
MSTRG.9873.1	0.275758866	0.310312571	-0.16430274	0.202407023	-	-	-0.13864379	0.296038483	1.327135173	0.012511131
MSTRG.99055.1	0.170984774	0.303350698	-0.83235459	0.008019361	0.062057998	0.796368442	0.112936857	0.062166167	1.029360208	0.00294757
MSTRG.99248.1	0.102578638	0.36281185	0.480874261	0.040961801	-	-	0.030153109	0.489074395	-1.111306037	0.017031684
XR_001994163.2	-0.163108615	0.831689631	1.388124594	0.01867489	0.790082386	0.883159834	3.775326874	6.41995E-05	-1.297027606	0.39466979
XR_001994164.2	1.819028457	0.05889099	1.499961244	0.015635816	-0.37530745	0.366877313	3.750062574	0.000824922	-0.072941878	0.949499379
XR_001994252.2	0.622698691	0.537988682	-0.161514	0.651557016	-1.58004324	0.006016089	-0.74516888	0.051849572	-0.366561453	0.267451945
XR_001994273.2	0.904055224	0.018351843	0.862938873	0.190383682	1.00739851	0.024338263	0.573932057	0.086561708	1.008028432	0.433490952
XR_001994277.2	-0.219928468	0.403970988	-1.81893096	0.003019665	1.913407732	0.061490973	0.673380924	0.293030507	0.985086001	0.132374957
XR_001994401.2	0.128833937	0.375168405	-1.69632281	0.029369999	-1.87539707	0.042497649	0.023886942	0.794611901	-0.61904565	0.101789025
XR_001994434.2	1.151788047	0.340866096	-1.43989078	0.232620667	-2.12875339	0.023275499	-0.45453624	0.289544396	1.714956839	0.544694523
XR_001994577.2	-0.035563603	0.835081824	-1.04425054	0.1415892	1.380418409	0.287370856	1.190666181	0.001543056	-0.976567222	0.242959583
XR_001994609.2	0.688683653	0.1334015	-0.64838597	0.008934058	0.22155987	0.617525452	1.404825631	0.043823303	-1.29332588	0.256931802
XR_001994662.2	0.437762616	0.016681163	-0.4829676	0.221561292	2.51414396	0.024314643	1.727381609	0.003604374	-0.435228709	0.532784667
XR_001994663.2	-0.329728318	0.357382692	-1.01170423	0.005765005	3.770354322	0.125066779	2.417628382	0.035635176	-1.205229697	0.173943154
XR_001994717.2	1.614839312	0.004271792	1.07456028	0.108631224	0.515231156	0.50253568	1.056720653	0.004316606	2.441619681	0.001606621
XR_001994738.2	1.134560188	0.117747864	-0.41031796	0.226566097	-1.76191674	0.670563374	3.282665625	0.000973318	3.354621384	0.000112667
XR_001994761.2	0.112433491	0.642226703	-0.44642869	0.227873389	0.440446411	0.179709315	1.292597143	0.008936346	-0.553330127	0.586617044
XR_001994779.2	2.195755827	0.000419457	2.74649726	1.44791E-06	3.256602957	0.00712467	3.088484511	4.81851E-05	2.336016717	0.037144463
XR_001994838.2	-1.021433849	0.001243268	-0.82731484	0.067614179	-0.70582681	0.07375835	-0.05787936	0.387371355	-0.999231222	0.024727176
XR_001994839.2	0.588185288	0.541308646	-1.04440267	0.000803258	3.98033963	0.091544039	0.366167902	0.049384303	0.098434826	0.937261681
XR_001994840.2	0.853745047	0.181816942	-0.53011289	0.01190266	4.143837652	0.161883216	1.269297268	0.00022238	0.469378073	0.679221378
XR_001994842.2	-0.427325363	0.33184113	-0.32212406	0.321330995	1.398550901	0.039262972	0.007802482	0.96595982	-0.999734883	0.097144901
XR_001994877.2	0.718972776	0.027589358	1.393316811	0.046114461	1.073498775	0.029928699	0.670375459	0.058845892	0.096575401	0.922757761
XR_001994911.2	2.553015811	0.19460438	1.09944757	0.029091384	2.43602607	0.001716085	0.364117586	0.088428222	1.386689681	0.260353926
XR_001994919.2	-0.099780428	0.802317988	-1.25992976	0.168779871	-1.23613225	0.031516211	0.434867373	0.122110431	-0.508760069	0.218946525
XR_001994932.2	0.331618129	0.125370894	0.594582691	0.019980998	0.974515715	0.092829165	1.725505974	0.00269516	-0.659304392	0.327168661
XR_001994945.2	-0.557643331	0.020793813	1.732469572	0.01083401	1.375280718	0.08862941	0.666073979	0.018909158	-0.584395512	0.21428565
XR_001994949.2	0.789142645	0.039750598	-1.55515789	0.015948831	1.491071408	0.16382616	1.118886334	0.033384411	2.376009735	0.001325878
XR_001994967.2	-6.818480017	0.01460689	-4.79409865	0.001186231	-2.70234102	0.500392501	2.66303803	0.001686703	-6.344628721	0.203890834
XR_001994968.2	-0.439864223	0.677205712	2.558133153	0.001706355	3.272016464	0.27354933	4.102120824	1.74525E-06	-1.970627013	0.36914751
XR_001994969.2	-3.71121841	0.010799276	0.560100402	0.266060531	-1.41801181	0.717717565	3.186808555	1.60763E-05	-0.824780699	0.708332553
XR_001994982.2	0.464621848	0.031856135	-1.72870565	0.000351985	2.274340278	0.294566156	1.081787513	0.01606996	-0.316681931	0.30044756
XR_001994995.2	0.50255901	0.210552607	1.298307146	0.001884035	1.782013352	0.309834258	1.079169691	0.003453488	0.69196552	0.104651875
XR_001995019.2	0.153616495	0.581764271	3.013943238	0.024478303	-	-	-	-	-	-
XR_001995084.2	-	-	-3.33522243	0.041809378	-3.12796699	0.005013692	-	-	-	-
XR_001995126.2	0.405921715	0.186376445	-0.06255488	0.879019269	1.630387336	0.036548037	0.271176979	0.364599525	0.344214631	0.734477271
XR_001995169.2	0.057920011	0.586331698	-0.5978233	0.24572504	1.540319287	0.005752382	-0.2330246	0.031751059	-0.207730265	0.264021042
XR_001995212.2	2.331119826	7.38173E-05	2.343215589	0.001179306	-0.09558316	0.926201844	1.827868311	0.000220642	-0.729823468	0.043027067
XR_001995256.2	-4.40389927	0.005088771	0.266796993	0.472627065	-1.18834695	0.730111736	-1.67500867	0.001464607	-4.440755806	0.110058886
XR_001995267.2	-2.979790233	0.00262494	-0.04760982	0.439798766	-	-	-0.06248926	0.249646232	-4.840136877	0.102254837
XR_001995287.2	0.079616547	0.121063063	-0.48521247	0.179244777	0.373020301	0.767341377	0.551319704	0.028871455	1.151008116	0.041568866
XR_001995352.2	-3.782650079	0.003301327	-1.78281754	0.000467484	2.095774103	0.001164227	-1.33180734	0.000574502	-3.261309521	0.074002683
XR_001995437.2	0.484265448	0.06682708	1.329163395	0.001394059	0.073640179	0.873036455	0.470042326	0.003552528	-0.089628279	0.731923483
XR_001995455.2	0.638442234	0.042728445	0.810157804	0.25461588	1.925544583	0.043821543	0.851522244	0.097600013	0.429429686	0.111540495

XR_001995474.2	-0.070298372	0.571471895	-1.38503844	0.312726334	-0.20189123	0.647472347	-1.16350824	0.039106389	-0.928573504	0.04930664
XR_001995517.2	0.203651658	0.37179002	-1.12155929	0.023837384	0.450585076	0.161083233	-0.01724606	0.901813356	-1.066713999	0.00648693
XR_001995625.2	-0.051473195	0.728504961	-1.80158699	0.036303195	-1.63329818	0.000957201	0.093565441	0.618763679	-1.99133783	0.056820188
XR_001995642.2	0.941273725	0.090626809	0.893687602	0.465889475	1.289578603	0.229625143	0.079711198	0.796807828	2.029108981	0.007961065
XR_001995643.2	1.450259438	0.006987119	-1.62101701	0.0053943	-0.40470272	0.322827623	1.438582867	0.120765251	2.633263288	0.055272568
XR_001995690.2	-1.687382336	0.003733398	-0.13283029	0.681268625	-	-	-0.31634756	0.101487913	-0.634227373	0.176610173
XR_001995701.2	-1.339768303	0.002998438	-0.40657404	0.291806821	-0.60338633	0.215028056	0.528221499	0.023695124	0.018533855	0.971215037
XR_001995702.2	-0.534485925	0.082341182	-1.86177242	0.012211518	0.290792969	0.810300156	-0.15166245	0.515175869	0.638908687	0.234158343
XR_001995754.2	0.533410785	0.007920696	0.423017114	0.490733988	0.490704846	0.229535161	0.627009967	0.00013358	1.269584246	0.004169591
XR_001995756.2	0.493193783	0.001373689	-0.00972394	0.992225743	-0.74693155	0.394491159	0.503523494	0.136959947	1.656437151	0.02179075
XR_001995759.2	0.974856943	0.00203508	0.428786363	0.237847001	1.600537599	0.274443652	1.378056737	0.002340751	1.243980485	0.026561916
XR_001995764.2	0.711607341	0.035944319	0.522789652	0.446208605	4.076367073	0.174152628	0.117879427	0.878414279	-1.976844484	0.04954352
XR_001995877.2	-0.153481621	0.270194648	0.739370231	0.243305861	0.95524163	0.537123989	1.051304821	0.018405845	1.348264796	0.142222138
XR_001995902.1	0.934745251	0.098276487	1.090882406	0.001627135	0.05031545	0.922544743	1.03524185	0.015672105	0.718350245	0.017119008
XR_001995916.2	-0.502130534	0.127510926	-1.08214791	0.004299518	2.889301597	0.176662344	2.474333968	0.065947473	0.524682595	0.314251555
XR_001995963.2	1.335482784	0.000187615	1.382042265	0.000670537	2.700717546	0.137603273	1.92427714	3.54147E-05	1.027850865	0.121158866
XR_001995964.2	1.260257314	0.01863004	0.970101977	0.000971312	2.275672947	0.146537177	1.504451074	0.023160177	0.761372091	0.028728068
XR_001996001.2	0.719393394	0.11447587	-0.61728946	0.031948205	1.77547307	0.165010717	0.344453199	0.490789973	2.806911804	0.010123575
XR_001996012.2	0.247662314	0.083953392	-1.25558861	0.000960535	2.34199804	0.275194402	1.196346555	0.101358688	-0.52671474	0.036137809
XR_001996034.2	0.172937678	0.37658635	-0.80528928	0.114681039	1.14473664	0.027370234	0.997662242	0.047089188	-0.273805332	0.23448042
XR_001996035.2	0.288921403	0.00712573	-1.62162708	0.005306553	1.369948868	0.16984633	0.686236395	0.003243422	0.095065885	0.761053352
XR_001996047.2	0.987091615	0.005196347	-1.48918927	0.002617745	0.573016965	0.460903868	0.942612607	0.002769034	0.140984376	0.349383087
XR_001996057.2	1.323673427	0.199923442	1.056570692	0.015507632	-0.09186738	0.874581419	0.133694284	0.386427847	-0.787285933	0.170767591
XR_001996083.2	0.898044161	0.026528331	1.009375258	0.000400073	2.408221619	0.167838336	1.094060709	0.000965507	1.383421629	1.09144E-05
XR_001996162.2	-0.689537948	0.111281776	-0.80392002	0.045650849	-0.52803104	0.093282352	-0.2835204	0.116077955	-1.104732468	0.036241415
XR_001996164.2	-1.270858374	0.061897735	0.104777993	0.892400099	-0.49645782	0.679999861	0.370321058	0.515458939	-2.370334964	0.008614284
XR_001996179.2	0.485281001	0.084684199	0.613780316	0.194914381	1.354727258	0.021362695	0.660828935	0.001989256	-0.375722035	0.194214515
XR_001996197.2	0.037149435	0.863429874	-8.9671784	0.011808482	-1.15960563	0.035661283	-0.33766283	0.001636394	-0.258618965	0.274251801
XR_001996202.2	0.637384385	0.048086478	-0.53764363	0.582838998	4.063907502	0.135264656	1.756854642	0.006963667	-0.340640806	0.489229928
XR_001996243.2	-0.833126741	3.41515E-06	-1.46701589	0.156942445	0.676527826	0.366110191	-0.77063165	0.07662786	-1.958599996	0.017634193
XR_001996304.1	1.229159712	0.007652257	1.789849809	0.087322374	1.792061337	0.031824828	1.497877506	0.000986341	0.705391343	0.08566237
XR_001996311.2	-0.684662922	2.99113E-05	-1.33647527	0.083974252	0.160974417	0.797281726	-0.90037305	0.121710847	-1.194697148	0.00048749
XR_001996312.2	-1.792537117	0.000241091	-1.68576552	0.174387663	0.807670369	0.49042426	-1.16308007	0.070654781	-0.415366818	0.06512555
XR_001996321.2	-0.822722512	0.004969807	-1.33232027	0.042233636	0.354688335	0.813055315	-1.75431485	0.001011183	-0.950231512	0.035311993
XR_001996351.2	0.73692719	0.016452152	-0.44551381	0.134310499	2.562267384	0.046510651	1.035989981	0.004267333	1.024227568	0.121828432
XR_001996362.2	-1.675432075	7.39852E-05	-2.49159148	0.020950433	-0.48793298	0.687033335	-2.98873919	0.000123551	0.248633793	0.839318864
XR_001996377.2	0.456898701	0.230278474	2.794444904	0.020998425	-2.23730022	0.219573097	-2.02621249	0.093932826	1.3628693	0.671829356
XR_001996394.2	-0.9591129	0.326909636	-2.92648586	2.85526E-05	0.647172518	0.506988229	-1.16791951	0.507606443	-3.724527733	0.015108664
XR_001996514.2	-0.330328491	0.373326404	0.467084379	0.009013897	0.334176264	0.598159266	1.369411622	0.004406934	-0.69528444	0.2537301
XR_001996522.2	0.006825393	0.967808825	0.397682339	0.694839238	4.126948581	0.081134473	0.727801973	0.008773989	-1.099918809	0.018291704
XR_001996557.2	-0.652843395	0.001524663	-0.75169639	0.24094483	1.417085694	0.023123822	1.355618724	0.115406849	-1.38465634	0.065182252
XR_001996626.2	-0.593754856	0.223977073	0.543313345	0.601721902	1.295621004	0.139937708	-1.39792615	0.044883153	-1.803106516	0.212042389
XR_001996664.2	-0.354331266	0.131493671	0.137985762	0.414207322	-1.56003687	0.00436335	-0.19644804	0.390900474	-0.110473825	0.779892111
XR_001996689.2	0.493917128	0.009974926	-0.4289746	0.349073657	0.801132593	0.117128655	1.921009525	0.026296701	-0.287843477	0.338650996
XR_001996792.1	0.010579292	0.901365847	-0.23270184	0.597033836	0.146738074	0.902543419	-0.21357208	0.113636301	-1.817440433	0.014128242
XR_001996793.2	-0.668634602	0.025217263	-0.86366908	0.041605776	1.924366173	0.221662891	1.532619204	0.000459948	-1.402749247	0.24582169
XR_001996832.2	1.374266499	0.002953726	-0.77685052	0.03430433	-	-	1.569896738	5.79977E-06	1.504316302	0.000320977
XR_001996839.2	3.490474627	0.009043869	3.661657934	0.028510346	5.026529514	0.003451102	3.035274334	0.057265162	-5.217283013	0.2326373
XR_001996859.2	0.534571505	0.219231553	1.746875029	0.000685878	0.69378106	0.31935318	0.257723239	0.157058298	-0.418635458	0.694470538
XR_001996875.2	-4.010560995	0.00599745	-	-	-	-	-	-	-5.675275184	0.102496141
XR_001996894.2	-0.277508294	0.152514527	1.261933907	0.082704562	2.500167857	0.016331663	1.137175997	0.002166515	-0.898864799	0.029982824

XR_001996920.2	0.648514817	0.543295101	-2.32127459	0.42938398	3.966909235	0.287116266	1.814145352	0.075961943	-2.704017982	0.001335462
XR_001996927.2	0.011421822	0.942378051	0.372442736	0.81743164	4.921161351	0.040595178	0.579815468	0.131370124	0.837969596	0.000922365
XR_001996945.2	-1.100122531	7.61147E-05	-0.44443235	0.477394234	-0.05872215	0.814687219	-0.63127985	0.017387524	-0.908131828	0.006951647
XR_001996962.2	0.45509191	0.01031375	-0.59864374	0.043972827	-0.1710875	0.614946472	0.539366228	0.053437954	-1.204826321	0.007098685
XR_001996977.2	-1.797623563	0.000360428	-3.03467995	0.031223019	-0.96017473	0.578236579	-2.58466041	0.001876604	1.046056564	0.662711255
XR_001997009.2	0.045253179	0.850442851	-1.73703877	0.02743472	0.087321246	0.908881492	-0.05109421	0.626216084	0.041472343	0.948495208
XR_001997032.2	0.975417982	0.042037801	-1.60413316	0.008669452	-3.21697407	0.02023646	-0.17328371	0.663658055	0.317233137	0.010990603
XR_001997201.2	-0.684955648	0.092911867	-0.21990744	0.676019	-1.51444532	0.102940805	-0.42983982	0.079301966	-1.372243972	0.019031251
XR_001997231.2	0.241168429	0.114943936	-0.43914662	0.338252656	1.114295096	0.081244779	1.014378539	0.002985489	0.278781354	0.05972773
XR_001997250.2	0.731133568	0.17924801	-1.45965499	0.003430654	0.111468776	0.846582507	-0.0366863	0.778096391	-0.518962093	0.21097615
XR_001997297.2	-0.025612273	0.899304297	0.035584039	0.931097941	0.267984502	0.583741733	0.097033124	0.601686637	-1.304161092	0.002620002
XR_001997331.2	0.158882236	0.185535707	0.491300864	0.029853903	-1.74527	0.005259299	0.114489514	0.643369264	-0.032011958	0.632849487
XR_001997428.2	-0.188382761	0.656925027	0.138028831	0.691605818	0.32091751	0.824150635	-0.07618453	0.200702032	-1.530188529	0.028688489
XR_001997476.2	-0.05326211	0.770537396	1.362114705	0.001540504	0.670463482	0.364872147	-0.20380056	0.779362836	1.056802901	0.507943556
XR_001997545.2	-	-	-0.27120875	0.622769703	-0.4828923	0.799168877	1.067463786	0.001473559	-1.78395143	0.008357636
XR_001997676.2	-3.230494974	3.2769E-06	-3.30999882	1.03278E-05	-2.40360183	0.020062291	-3.77210649	2.9337E-05	0.642733557	0.792797926
XR_001997677.2	0.478068263	0.422490678	1.371202721	0.127428541	0.268026982	0.826426457	1.705255403	0.008467512	-1.379477726	0.003344868
XR_001997689.2	0.670091131	0.010575966	1.04937543	0.030642078	0.868122307	0.01402529	0.899852968	0.001333826	-0.148547751	0.735464681
XR_001997746.2	-0.263005789	0.186486813	0.668928476	0.491328384	-1.53560759	0.326021336	-0.77052231	0.023023085	1.327684984	0.045743779
XR_001997771.2	-0.616544626	0.278747211	-2.31608497	0.016342453	-0.69948181	0.085653087	-0.36112942	0.062836973	-1.353055818	0.008854958
XR_001997935.2	0.958502394	0.152236945	-0.62792861	0.489588826	2.334695004	0.286544831	2.616441472	6.9937E-05	0.142847882	0.822637398
XR_001998005.2	-0.522306897	0.157386538	-1.26153361	0.005581452	-1.12791391	0.009929873	-0.09138585	0.736766626	-1.100234565	0.008367784
XR_001998020.2	1.201608015	0.010563931	1.834512693	0.008146768	1.153992994	0.20359404	2.66543188	4.97458E-05	-0.17271655	0.699550841
XR_001998022.2	0.261238515	0.002259091	1.323392627	0.022364867	1.505325394	0.209213457	0.387894124	0.000332454	-1.045918558	0.011778375
XR_001998063.2	1.902393264	0.001621238	1.579047601	0.000866174	0.218513339	0.768412316	2.380669457	1.63872E-05	0.398878486	0.167888655
XR_001998161.2	0.835728856	0.054230396	0.756731472	0.064631473	1.92471819	0.0003822	1.132395064	0.002010196	0.551894678	0.050991014
XR_001998193.2	0.045291995	0.282618906	0.000707897	0.998646956	0.156565901	0.833354111	1.274978953	0.005014553	-0.99215365	0.458936896
XR_001998246.2	-0.987971298	0.029473235	-1.09550262	0.030441654	-0.70906293	0.532200616	-1.28425025	0.115484308	-0.61368556	0.06127934
XR_001998253.2	-1.017061281	3.2372E-05	-	-	0.70177619	0.143260069	0.340050333	0.002102955	-0.417347591	0.107249287
XR_001998322.2	0.523299601	0.598833615	-2.0463153	0.000251831	0.99891389	0.328119684	0.488765591	0.417255725	2.807511679	0.02441184
XR_001998388.2	-0.744290031	0.098579121	0.651030086	0.056891326	-4.55321688	0.100952355	-2.03377151	0.00656313	-0.69011859	0.250124145
XR_001998539.2	0.982318255	0.292871826	-0.40578159	0.453393764	-1.60019334	0.433364171	0.851978729	0.026103224	1.711212222	0.006064119
XR_001998553.1	-0.879308079	0.004807738	-0.15013205	0.58714585	1.270030293	0.60072986	-0.54205162	0.034551445	-2.082597341	0.022717578
XR_001998573.2	1.145050259	0.136134535	2.923721366	0.054961627	3.231376598	0.01144054	3.318181191	0.000384587	-0.588410535	0.723316321
XR_001998574.1	-	-	-	-	-	-	0.83641187	0.000884311	-1.396295203	0.029982669
XR_001998586.2	0.502126797	0.331191027	3.245284277	5.73267E-05	-3.83814543	0.100255732	-0.10099353	0.535022941	1.039232685	0.015263157
XR_001998588.1	0.409478159	0.373343228	0.89193013	0.028838373	-2.99499629	0.135888611	-0.32104194	0.161003764	1.136838929	0.015394303
XR_001998591.2	-0.850762786	0.156555445	3.272567244	0.017946049	1.766591131	0.283202736	1.07821463	0.00250258	-0.713306555	0.526799387
XR_001998593.2	-0.392819682	0.549397311	1.091314139	0.025527794	1.266479997	0.443496826	0.95614647	0.011377813	0.028025496	0.962808103
XR_001998631.2	-	-	-4.53760246	0.006864927	-8.58048211	0.0119412	-	-	-	-
XR_001998632.2	-	-	-3.35426853	0.000564834	-7.2646675	0.022858517	-	-	-	-
XR_001998635.2	-3.248654629	0.012555313	0.079663416	0.512904859	1.905687103	0.516055611	0.378060755	0.192231349	-0.997817043	0.075479267
XR_001998636.2	-2.038924887	4.16721E-05	-4.77896812	1.95731E-06	-2.35379669	0.007702471	-1.95466494	0.000482253	0.615026905	0.795985159
XR_001998656.2	3.305261199	0.000648996	2.289691316	0.002775462	-0.3321404	0.525533992	2.562194042	0.011321113	1.691551746	0.000398668
XR_001998713.2	-2.807377719	0.000269819	-0.94990518	0.000152426	-1.09289552	0.429773006	-0.78605159	0.110063663	-2.177445844	0.010651322
XR_001998763.2	-1.103362419	0.002087383	-0.04414335	0.278164187	1.11029696	0.142337007	-0.82461786	0.019388093	-2.56388329	0.028862315
XR_001998880.2	-4.093682732	9.54755E-06	-3.84694396	0.000558235	-2.62974762	0.008295926	-3.20770486	0.000941305	-4.823644876	0.003294751
XR_001998909.2	0.796298178	0.006138005	0.559289579	0.582874451	4.639824547	0.189437066	2.628876505	0.01019678	0.948093265	0.304803963
XR_001998948.2	0.130710978	0.627539181	-1.95552029	0.042144558	1.394953901	0.286399119	-0.02160545	0.954229204	-0.367978488	0.499163985
XR_001999023.2	-2.450931575	8.50763E-06	-3.01674432	0.000218878	-3.56248628	0.12871091	-3.99553745	3.95142E-05	0.434539024	0.397873296
XR_001999024.2	-2.985534975	0.001768275	-2.76567973	0.001330333	-3.93846965	0.120121083	-1.93248791	0.00564767	-1.1647299	0.36611112

XR_001999026.2	1.475737581	0.031762239	-0.6727449	0.023275948	1.723510015	0.053843232	2.091496387	0.006661476	-0.308668402	0.36552849
XR_001999031.2	-0.379231043	0.146164342	0.602594157	0.307929146	-4.06141727	0.161965932	0.913572347	0.029112737	1.510801634	0.016839664
XR_001999046.1	-0.396932854	0.047663985	0.226713622	0.56314039	-1.37733167	0.020995093	-0.03771687	0.878914912	-0.258093023	0.312672145
XR_001999091.2	0.761361711	0.059074864	-1.07739079	0.176861331	2.660105524	0.193188557	1.607547511	0.000282735	-0.78170245	0.138498422
XR_001999180.2	1.090456511	0.015550483	0.68426975	0.02737624	0.571722977	0.575797278	1.252142636	0.00212279	-0.169060281	0.062068821
XR_001999184.2	-0.440177345	0.017699088	-0.99624885	0.165461495	1.679468295	0.049659482	0.402785184	0.418645561	-1.726718199	0.225109461
XR_001999201.2	2.605408428	0.019665055	4.395450059	4.69716E-07	7.191728946	0.113546585	4.642133593	8.48583E-07	3.926065702	0.001340358
XR_001999202.2	0.844287375	0.376877677	-0.0092301	0.429975537	-3.31647446	0.600483618	3.782153549	0.000223137	-1.173091525	0.082525804
XR_001999264.2	-0.581897486	0.122967299	-1.36048329	0.045881399	0.722805938	0.324292988	-0.00726186	0.964099193	-0.327828209	0.354320132
XR_001999291.2	-1.680217847	0.002125858	-4.04505282	0.004343043	-0.2825301	0.26560551	-0.19800202	0.814457387	-1.222740995	0.24546851
XR_001999335.2	-0.264979392	0.045611957	-0.62366712	0.000414279	1.216701908	0.0086338	0.707443902	0.068658371	-0.464289201	0.312080754
XR_001999338.2	0.019371526	0.953270041	0.614011482	0.484624641	-1.90981347	0.016267527	-0.2013885	0.440041645	-0.072344661	0.876096822
XR_001999397.2	1.262753898	0.002630011	-0.91913664	0.432268171	4.875597415	0.17379126	2.166810839	0.026240851	2.520416558	0.049804794
XR_001999403.2	-0.027654091	0.947423827	0.780954394	0.331777918	1.684949365	0.032673254	0.241115098	0.504281936	-0.356557391	0.797172109
XR_001999476.2	-0.537473763	0.026740478	-1.39349539	0.00105352	0.341092124	0.200984038	-0.07473367	0.094234616	-0.825293143	0.003351245
XR_001999527.2	-0.339837492	0.035808418	-0.95067535	0.005842739	0.808531499	0.089257776	0.030778781	0.910287902	-1.332199376	0.031775343
XR_001999549.2	1.331550755	0.004982968	0.233681728	0.605697511	3.211789042	0.009170257	1.416363993	0.358266121	-0.988448685	0.188120986
XR_001999565.2	-1.971754099	0.069801643	-0.04008313	0.907031395	0.113759111	0.838372357	-0.14346717	0.565136788	-2.473568937	0.047333173
XR_001999566.2	0.016874068	0.868575831	0.734183106	0.255691775	0.148082033	0.343553865	1.232282015	0.005640518	0.729045494	0.018917511
XR_001999578.2	1.661752828	0.003053783	0.793226712	0.409386106	-0.20129551	0.870897641	1.988769086	0.000291603	1.151585392	0.049502415
XR_001999698.2	0.253865596	0.080720963	-0.2780752	0.610566318	1.807424498	0.175975243	0.547588119	0.03644514	-1.097939805	0.021048076
XR_001999707.2	0.349529022	0.056037837	-0.53519326	0.533010593	1.074541357	0.000883133	0.481255575	0.035882046	-0.138811298	0.672839734
XR_001999772.2	-0.472096665	0.001180819	-0.32312079	0.238847314	-0.42761208	0.373952856	-2.49815846	0.040175757	0.335646025	0.639535625
XR_001999832.2	-2.024469501	0.0019654	-3.34053774	0.000123874	-1.53606861	0.727791946	2.64610795	0.170526988	-2.132428353	0.300122562
XR_001999867.2	0.259661044	0.062452458	1.401408397	0.009667035	0.357719259	0.541785243	1.480818719	0.017958602	-0.605792439	0.170219044
XR_001999896.2	-1.021101559	0.126810314	2.401873112	0.053735834	2.546108815	0.56147177	1.988440779	0.006658583	-2.003449253	0.05707517
XR_001999938.2	-0.091901539	0.042425298	0.134889332	0.261976663	1.195939923	0.018122026	-0.48823538	0.069437874	-0.623955413	0.004989858
XR_001999980.2	-1.392385022	9.56525E-08	-1.18320002	0.071459943	-1.50944074	0.024179622	-1.09464186	0.008246025	-1.361926845	0.002114662
XR_002000537.2	-0.313838703	0.015002048	-1.20329033	0.036799724	-0.13316518	0.641539709	-0.06490496	0.432651401	-0.20378552	0.027775651
XR_002000562.2	-2.243645793	0.00020793	-0.02387729	0.872973203	-0.77056565	0.026315487	-1.71397714	0.003430094	-1.555879081	0.005841105
XR_002000563.2	-1.206158473	0.000244047	0.343436942	0.003619338	-0.51481855	0.55905063	-0.80978462	0.004305294	-0.328371041	0.181097312
XR_002000652.2	1.668917172	0.000623153	1.516936176	0.021371229	1.775664027	0.132971877	0.529261917	0.428323314	-0.66603925	0.225931672
XR_002000661.2	0.502147902	0.338572282	0.506582685	0.616646418	0.881344154	0.285738956	1.538852242	0.002781625	-0.846611177	0.134554321
XR_002000714.2	-0.323177636	0.039171576	-1.14747906	0.021958472	1.547539114	0.020304892	0.7385942	0.047335535	-0.552815368	0.090390769
XR_002000768.2	0.073640465	0.149777332	-0.23820739	0.595956115	-1.62467834	0.033310655	0.143476636	0.582574242	-0.104812424	0.822592545
XR_002000772.2	-1.517333082	0.000110885	-1.50306655	0.028469602	-1.10601744	0.005386954	-1.93019506	1.04037E-05	-1.245465855	0.046119546
XR_002000793.2	-0.670506979	0.166028921	-0.20371302	0.627549614	-	-	0.252164041	0.358296671	-1.23348156	0.014262049
XR_002000805.2	-0.318456547	0.640415717	2.175480316	0.002491742	6.810146322	0.407799227	1.643312844	0.079545918	1.747974564	0.030411821
XR_002000813.2	-0.267566996	0.079913032	1.148773795	0.00145228	-0.69404419	0.334361532	0.944915974	0.002491453	-1.115438281	0.378987673
XR_002000880.2	0.257897874	0.361029058	0.186286961	0.461528094	0.87217731	0.173874828	0.803907301	0.000214458	-1.232423761	0.012628074
XR_002000923.2	-	-	1.081610243	0.386405654	4.673571323	0.058950903	2.245810171	0.035373208	-0.853724197	0.286101016
XR_002000928.1	-0.183268259	0.038849457	0.03637078	0.861003753	1.06398252	0.007413523	0.286432329	0.028759148	0.326369664	0.071176063
XR_002000937.2	-0.178393136	0.192886065	-2.30875413	0.011091554	-0.70392094	0.40328345	-0.2561471	0.110808407	-1.417763053	0.083132346
XR_002000950.2	-4.920372298	0.006930484	-1.64410099	0.004904156	-3.27339575	0.075770781	1.280524192	0.000486902	0.444706065	0.45945524
XR_002000951.2	-4.637700523	0.000751929	-4.28830893	0.001071851	-3.29814472	0.021018811	-2.07898915	0.081785731	-0.076092651	0.946624658
XR_002000952.2	-2.390460061	0.004293007	-1.76543093	0.001290845	-0.35943688	0.428996451	-0.13555677	0.33086453	-0.148750855	0.890450231
XR_002000967.2	0.19267891	0.060287643	-0.28686993	0.252705081	1.232513233	0.090368235	0.872764374	0.004228432	-1.040615734	0.008609967
XR_002000995.2	0.011729494	0.364118934	-1.28344803	0.00228149	-0.79720681	0.005157642	-0.36398871	0.088411849	0.108929698	0.802458956
XR_002001046.2	-1.988839087	0.000355396	-4.37552781	0.00030537	0.448054511	0.774219415	-0.00838912	0.963137072	1.006866369	0.077315909
XR_002001049.2	-0.682205645	0.23459419	-2.15806435	1.76412E-05	3.369703588	0.390407619	2.100194467	0.00947535	0.032076642	0.962229849
XR_002001072.2	0.080380831	0.201165106	0.196199422	0.54714874	1.355885092	0.024251508	0.316974964	0.185766638	-0.403682926	0.294892266

XR_002001178.2	0.637609143	0.225483217	1.588126494	0.000121635	2.269416906	0.092517254	1.640722657	6.06616E-05	0.160072496	0.813880553
XR_002001196.2	3.652093961	3.23639E-05	0.794773466	0.175731781	-1.58692429	0.296755073	3.629684451	2.59545E-05	3.061196885	0.000249914
XR_004797573.1	-0.933933838	0.445161573	-1.04173714	0.63491612	-1.5307436	0.019999868	-1.04890131	0.189371782	3.308338095	0.15181847
XR_004797620.1	0.019740131	0.874810518	-0.7839066	0.273367988	1.985634097	0.029012175	0.286132987	0.110488808	-0.053537612	0.884482711
XR_004797728.1	1.371690675	0.04103054	0.718985	0.00979238	0.241867141	0.781673866	1.859035078	1.11841E-05	1.486095112	0.002506842
XR_004797737.1	0.007989264	0.941751202	-0.06256987	0.845186166	1.056326805	0.004803409	-0.02297037	0.854770005	-0.21522129	0.25503035
XR_004797755.1	-0.315883478	0.677275969	-0.53121511	0.609746035	0.156032051	0.866080111	-1.62997335	0.010156837	0.472061274	0.543780481
XR_004797765.1	0.19593264	0.072898573	-0.87264006	0.243354202	-1.09260146	0.006879649	-0.33953241	0.010013285	0.024443228	0.958469867
XR_004797770.1	-0.558150569	0.232142339	1.6996853	0.039706073	1.287383851	0.017641669	-0.19415696	0.053906722	-1.123801355	0.009477867
XR_004797775.1	-0.370337389	0.08719717	-0.61719841	0.138534727	-0.08913553	0.727188948	-0.47088004	0.166417823	-1.130496877	0.015352892
XR_004797789.1	-0.749732704	0.283154242	1.343299468	0.00300158	1.743058265	0.082176858	0.035502644	0.903884581	0.586817775	0.335431246
XR_004797794.1	0.85466898	0.013875822	-0.8252189	0.476137658	3.023459905	0.243214733	1.59427424	0.049441111	-1.034741819	0.084711124
XR_004797800.1	-1.00754017	0.00016761	-5.06071267	0.0064576	-9.60224549	0.02885795	0.743184738	0.584574486	-1.853660828	0.621771796
XR_004797810.1	-3.498188809	0.029141465	-3.36501073	0.000357162	0.230848675	0.923555398	1.581616324	0.288647112	-3.644938215	0.003391685
XR_004797931.1	-0.800661874	0.02367256	-1.35483322	0.032422937	2.835311201	0.387326068	1.545175889	0.055335345	-2.76426777	0.076084427
XR_004797936.1	-0.241983718	0.205419032	-0.00657115	0.98840142	3.160242652	0.01068644	1.408093734	0.000780541	-0.719618002	0.066737761
XR_004797937.1	-1.184564446	0.002834633	-1.27041432	0.02493972	-0.39214986	0.321915981	-0.45063168	0.037593776	-0.882962886	0.017929149
XR_004797954.1	1.579307037	0.203407504	2.682303297	0.001490543	-1.32793704	0.106354098	0.738828985	0.032836503	1.676301835	0.006213662
XR_004797956.1	-0.91845737	0.014987756	-1.87907798	0.009915353	-	-	-	-	-2.363002078	0.085031918
XR_004797967.1	0.102125935	0.789270477	0.135840218	0.429644924	-1.36522745	0.031958982	-0.17780921	0.172778434	-0.291671245	0.694405056
XR_004797991.1	-0.158966482	0.114545077	-0.09894413	0.853895658	0.472728936	0.329993172	-0.11780444	0.467658543	-1.270672303	0.003494828
XR_004798004.1	-0.407581925	0.57374736	-1.38980823	0.000937518	0.810364223	0.238079089	1.053563352	0.043469916	-1.665293737	0.033638359
XR_004798006.1	2.283440553	0.000568196	2.302329121	0.290027912	-0.95063173	0.712997749	0.626333992	0.600669712	-0.302190525	0.895155317
XR_004798044.1	-0.096577855	0.706867552	-1.60938778	0.017304587	1.545889134	0.365737046	1.871603455	0.067166245	-0.0186585	0.946835852
XR_004798068.1	0.689250866	0.0090932	-0.66093083	0.043299912	-0.71876578	0.75180775	1.594831827	0.021246623	-0.329336569	0.38609953
XR_004798070.1	-	-	-0.97117153	0.177887159	-1.15881468	0.030363018	-	-	0.008942795	0.095819942
XR_004798071.1	0.134677535	0.486113185	1.104227141	0.004485182	0.029011808	0.901908803	0.471418458	0.002260995	-0.606246566	0.041175911
XR_004798073.1	-1.183346171	0.031981147	0.096818746	0.83460826	0.385303107	0.388789211	-0.05491855	0.818020396	-1.760559044	0.023382027
XR_004798263.1	1.557859092	0.000745421	2.066974746	0.005179086	4.090522338	0.107684532	1.273955037	0.017476899	-0.607830927	0.559899595
XR_004798313.1	-0.345240821	0.332107233	-1.83921884	0.007070847	0.415726523	0.834204713	0.259880927	0.149017004	-	-
XR_004798329.1	-0.475368686	0.025212331	1.775178407	0.017188332	-	-	-	-	-	-
XR_004798341.1	-0.726140178	0.047832822	-0.7828583	0.139353988	1.560161969	0.010297526	-0.27986971	0.121227526	-0.736736662	0.075558045
XR_004798360.1	-1.090532192	0.138522642	0.79104545	0.004522588	3.767533275	0.006454263	-0.57332155	1.45858E-07	0.518048014	0.459392821
XR_004798425.1	0.011066428	0.586092176	-0.3094927	0.016854826	0.013105229	0.881504904	-	-	1.198210586	0.00598621
XR_004798427.1	-0.894379802	0.04158346	-0.95637791	0.051423292	0.269772423	0.670780831	-1.27043568	0.00150565	-1.591684773	0.003109955
XR_004798428.1	0.648695066	0.009903313	1.490374005	0.000133583	1.018247556	0.114939313	0.337996009	0.094840498	1.028349658	0.089440926
XR_004798447.1	-0.511708969	0.102835703	-1.6720503	0.010342601	0.47181796	0.576856326	0.58799097	0.023548999	0.419789694	0.406010334
XR_004798464.1	-0.472071904	0.041840403	-0.68153201	0.05914147	1.237847694	0.021360465	0.481655554	0.016180364	-0.426247543	0.043108629
XR_004798470.1	-	-	-0.12910592	0.808124856	-	-	0.280529047	0.023392346	-1.074295017	0.019653572
XR_004798488.1	0.783107438	0.017429704	0.022387201	0.967001537	-0.3446645	0.742746297	1.264164926	0.000583545	1.024785423	0.011745725
XR_004798497.1	1.210438853	0.008897454	0.829909008	0.113461483	1.064478246	0.019785348	1.074526568	0.000604375	1.009787354	0.000515729
XR_004798500.1	-1.046771521	0.001367526	-1.95298549	0.000702676	-0.88186986	0.327516081	-2.47653222	0.001516131	1.062579431	0.322024912
XR_004798525.1	-0.29793358	0.001467234	-	-	-1.81432559	0.089647665	-1.29206643	0.00330913	0.571891619	0.59940495
XR_004798526.1	1.605003982	0.003735793	-0.94213133	0.003210769	2.63931922	0.31681896	2.916161397	0.068139488	-2.185638957	0.471047392
XR_004798527.1	0.643915622	0.176761535	-0.86590612	0.005356982	3.642191928	0.233781427	1.907868366	0.006729079	-1.844254206	0.29478058
XR_004798534.1	-2.127116041	0.012843695	1.361337605	0.271467034	-1.68928001	0.504740303	-0.30450694	0.351523993	-3.278005914	0.09340871
XR_004798542.1	0.41331914	0.114811739	-0.06761291	0.948339177	-2.09793062	0.008080878	-0.19335412	0.472391953	0.221135263	0.81264569
XR_004798548.1	-	-	-0.61368435	0.023870912	-3.63378781	0.045913044	-	-	-	-
XR_004798574.1	-1.142696669	0.116589229	1.513906301	0.023831679	-0.70818678	0.22111276	-0.08983975	0.647603175	-0.794473939	0.004506435
XR_004798595.1	-1.223016307	0.01424137	-0.86705462	0.036249918	0.069509104	0.860276367	-0.49784227	0.025661814	-2.042782841	0.00894821
XR_004800813.1	0.391597312	0.091637113	1.168496572	0.004389248	0.503650214	0.277177821	0.629618009	0.079066177	0.550818684	0.420691033

XR_004800858.1	0.76105023	0.136873532	-2.68963281	0.104974426	1.224722845	0.022976744	0.015352327	0.940137519	-2.022357649	0.184433656
XR_004800862.1	-0.61930608	0.015577387	-0.90569689	0.159818643	0.784000473	0.12978219	-0.43617228	0.021028249	-1.62284094	0.023005986
XR_004800885.1	1.873602493	0.006409355	1.00249073	0.047185592	3.064381834	0.095265973	1.403750393	0.006645616	-0.097301373	0.810343272
XR_004801037.1	0.217363388	0.480055862	-1.33973815	0.050269078	2.966345519	0.036199639	0.904423413	0.025675844	0.825553316	0.142305618
XR_004801039.1	2.243758504	2.54124E-05	1.826506719	0.001341298	-0.10649799	0.872513852	1.548886638	0.003770875	1.862439901	0.182640571
XR_004801042.1	1.180558366	0.004850708	0.766094023	0.199736167	0.947019642	0.056179108	1.168781476	0.007052415	1.216191876	0.006461395
XR_004801045.1	0.237055491	0.518662035	-0.13076154	0.5106281	1.131212241	0.014151048	1.095833567	0.013754883	-1.199307424	0.223459603
XR_004801048.1	2.215536765	0.001745888	-2.52239306	0.01856998	0.214497257	0.33919924	0.927686519	0.003148733	-0.168440681	0.703969031
XR_004801052.1	1.152250828	0.053659889	-0.22718664	0.733325734	-1.22759948	0.553612065	1.469256565	0.00153125	-1.461108496	0.087555494
XR_004801054.1	1.286633362	0.009509652	0.071252644	0.16202397	-	-	1.634844842	3.55702E-05	-1.561426918	0.209938653
XR_004801062.1	0.352895218	0.063735715	-0.61610817	0.437802673	-1.59268683	0.722940912	3.400613829	2.78229E-06	2.910599881	0.001361385
XR_004801094.1	-0.496245614	0.000959388	1.44284179	0.000489262	1.644006563	0.312519863	0.992181829	0.001927575	-1.310914872	0.183704922
XR_004801106.1	1.083344229	0.041405928	1.296666607	0.003191802	0.622627541	0.26871722	0.437948935	0.004746019	0.628527803	0.072460691
XR_004801119.1	1.635742199	0.001673777	2.152579258	0.039583126	2.042271315	0.212006617	1.226567395	0.047001941	-1.214763618	0.084213368
XR_004801128.1	-0.781549366	0.137700773	-1.18584062	0.011385363	3.502196715	0.027735867	0.496746447	0.390613664	-0.572568991	0.411994979
XR_004801156.1	-1.301787225	0.012733083	-0.60343384	0.10819318	-0.05191789	0.849565399	-0.58984457	0.002250344	-0.594554428	0.004763888
XR_004801159.1	0.499786411	0.018276834	0.785327	0.006238438	2.199582584	0.045557876	1.109360166	0.012378036	-0.084318552	0.852085217
XR_004801170.1	-0.434655414	0.08359462	-0.95094755	0.007782252	0.233040931	0.844764028	0.097149462	0.837412464	-1.492648373	0.004979981
XR_004801179.1	0.270508233	0.328965698	0.45247417	0.211509122	0.94351305	0.209078041	0.681029554	2.44176E-05	-1.08984457	0.00621123
XR_004801190.1	-0.23830783	0.065216937	-0.58268776	0.201738076	1.171214086	0.039669213	0.17319682	0.04587586	0.03787446	0.534555483
XR_004801195.1	-1.674304474	4.96347E-06	0.023516335	0.829807992	0.192236126	0.584009238	0.1030143	0.208745764	-2.392945491	0.165796613
XR_004801273.1	-1.189424918	0.005481097	-0.1645263	0.547529081	0.403285643	0.325216624	0.100421713	0.024617287	-1.340818692	0.008649101
XR_004801285.1	0.403584041	0.084412333	1.111694367	0.052365395	0.376350816	0.401156445	0.441036966	0.00429491	-1.046762366	0.006645309
XR_004801297.1	0.224636884	0.329393887	1.099029641	0.004986935	-	-	0.15714713	0.186564029	-0.050183589	0.830643611
XR_004801324.1	-3.757293934	1.75712E-05	-3.95487833	0.004776757	-2.56053296	0.009705741	-3.55625336	0.000128706	1.257806439	0.585986533
XR_004801337.1	-1.030854868	0.005225049	-0.51209771	0.001159947	0.282768908	0.197914211	-0.3667175	0.005019607	-1.215350541	0.010979773
XR_004801348.1	0.012344653	0.896914394	0.50670732	0.150047908	3.313704473	0.018429683	0.380980685	0.001433838	-0.448966081	0.085824967
XR_004801412.1	1.299514753	9.40465E-05	-0.62715416	0.028047212	1.812560461	0.028892534	0.341664848	0.09871987	2.329031878	0.088896183
XR_004801425.1	-0.084378158	0.571318529	-0.67850263	0.118200976	-1.03958608	0.011885937	-0.92667117	0.002073363	0.201968014	0.6246813
XR_004801500.1	1.18922338	0.007873765	0.224269776	0.586207587	1.746460327	0.030320724	1.040004286	0.005411539	-0.757088127	0.092675597
XR_004801510.1	0.86317701	0.003161447	-0.58605391	0.078937348	-0.17487459	0.335597456	1.276572017	0.001782612	0.091349884	0.620402172
XR_004801521.1	-2.814592637	0.000269734	-1.34043599	0.361467577	-1.1539551	0.036077532	-2.30749151	0.001039571	-4.285968734	0.020721588
XR_004801524.1	-2.005880882	0.007356672	-	-	0.832001482	0.34784787	-1.19667261	0.002220038	-2.666847643	0.231572434
XR_004801535.1	0.911148662	0.000657628	1.145391192	0.002459745	0.970936499	0.041842662	1.456796299	3.44436E-05	1.244872959	0.00067698
XR_004801589.1	0.229578007	0.231525849	1.103991018	0.018525497	0.089841164	0.506470903	0.100161581	0.055458357	0.059747312	0.788757664
XR_004801644.1	-0.241389061	0.735530125	1.647433561	0.004673469	1.302614088	0.252950517	0.049892171	0.663679746	-1.595908371	0.017049747
XR_004801668.1	-0.083172349	0.195071045	-0.66512613	0.002598534	-0.43521414	0.297004242	-1.13417667	0.002213533	0.410154441	0.786356586
XR_004801671.1	-1.43175193	0.009919594	-0.32056763	0.532377596	-1.39066453	0.393678831	-1.57951344	0.016902608	-1.666148092	0.034053951
XR_004801690.1	-0.856830119	0.047802896	-1.31807087	0.003289887	-0.45314452	0.490881128	-1.02538777	5.83447E-05	-0.62846751	0.227747429
XR_004801698.1	1.203996615	0.008505966	1.896669434	0.000110192	1.868120352	0.010599692	0.755660937	2.87703E-05	-0.036870297	0.917148416
XR_004801700.1	1.002197986	0.003594883	1.68102166	0.000125381	2.077698169	0.109570922	0.646182889	2.61756E-05	-0.046025595	0.874693609
XR_004801701.1	0.724873925	0.001698308	1.542732334	0.001219772	-0.73842331	0.561553153	0.495325729	0.000584975	-0.003681357	0.988343624
XR_004801702.1	0.920168217	0.001689256	1.692125768	0.00018656	-0.8224385	0.559437194	0.583542489	0.000245713	-0.044177382	0.787639834
XR_004801712.1	1.662239328	0.002671263	2.257065192	0.010656785	2.023683956	0.02793157	0.850227105	0.014536331	0.361896084	0.098475157
XR_004801727.1	-0.135632156	0.077605294	-0.2454034	0.214351167	1.80529836	0.011699382	0.083755505	0.64604154	0.061837825	0.88447463
XR_004801737.1	-0.61736661	0.002123513	-1.69682757	0.003218914	-2.60988266	0.048449389	-0.05167574	0.249668629	-0.36054007	0.429939284
XR_004801774.1	2.982946171	0.035005341	1.04884603	0.178687479	-5.44696604	0.096994154	-2.5578514	0.157540686	0.797721958	0.76613735
XR_004801872.1	-1.036936772	0.008257273	-1.51968431	0.002681952	-0.67245126	0.289527784	-0.26032155	0.65878364	-2.046747036	0.066406082
XR_004801874.1	-0.786423721	0.000372028	-1.06539044	0.024422914	-0.19261865	0.7200761	0.162624429	0.480486759	-1.360888969	0.025453243
XR_004801887.1	0.289487837	0.353764572	1.127283294	0.001431103	1.361836132	0.014713185	0.707613448	0.021638457	1.195251162	0.089471293
XR_004801901.1	-0.125255835	0.70081896	-1.46784114	0.00389783	0.84830877	0.206631469	-0.49448809	0.263422007	0.277334545	0.43547511

XR_004801903.1	-0.301980141	0.058792635	-1.20103303	0.001227622	0.741484257	0.10603289	-0.19417765	0.219973012	-0.000420814	0.999207613
XR_004801905.1	-0.4597555	0.008604586	-1.21647028	0.030575041	0.093151194	0.856564095	-0.76384691	0.023972187	-0.98444492	0.038110817
XR_004801910.1	-	-	-	-	4.351981473	0.035709833	-	-	-	-
XR_004801964.1	1.351954111	0.104380385	3.91051167	0.014856063	4.180896798	0.15022474	2.882176556	1.25424E-07	-0.112117492	0.883280991
XR_004801970.1	1.137530882	0.000150509	2.175941681	2.8088E-06	2.00729686	0.076072353	1.647267629	5.52657E-05	0.941071833	0.169976153
XR_004801980.1	0.258893599	0.234223152	1.056761387	0.034150834	0.843225023	0.362734396	-0.01327813	0.939869882	-0.329170152	0.598595054
XR_004802018.1	0.561570435	0.003946853	1.5034313	0.132140951	1.370631301	0.05196736	1.312409709	0.004858878	0.13273761	0.734881683
XR_004802020.1	-	-	-0.52562273	0.806374865	-	-	-2.13678081	0.0031647	-0.150456832	0.913082845
XR_004802024.1	-0.576129621	0.003291193	-1.05644044	0.037701679	0.49193514	0.503928576	0.605440368	0.002079418	-0.717045005	0.089752266
XR_004802027.1	1.234284634	0.005529023	-0.7635341	0.374758114	2.44775402	0.289958746	1.896408504	0.004597862	0.008469483	0.99083636
XR_004802041.1	-0.310199306	0.106949079	-0.1461832	0.82936036	-	-	0.185121432	0.349993561	-1.164305991	0.033617502
XR_004802179.1	-0.55657257	0.006094528	-0.77903012	0.004388079	-1.14104007	0.035818571	-0.67652144	0.002010293	0.04350566	0.880418449
XR_004802185.1	0.79947399	0.005729814	0.195628973	0.667699566	0.579589749	0.326688576	1.202142506	0.012407658	-0.35731503	0.608794664
XR_004802196.1	0.413285138	0.314488511	1.147204264	0.10523538	0.838199917	0.690771664	1.308880493	3.77355E-09	0.891684228	0.004895607
XR_004802266.1	-2.316204702	5.56034E-05	-1.28329396	0.041940943	-1.09899533	0.373111364	-1.30598361	0.015610114	-2.374424091	0.035101839
XR_004802274.1	0.845460236	0.154225545	0.158205381	0.733743501	-1.35137997	0.178015306	1.576514931	0.003945529	-0.415808113	0.18396678
XR_004802373.1	-0.120205401	0.431991881	-1.10718563	0.057111686	1.305367291	0.133615273	1.779368493	0.086023555	1.003667069	0.00079777
XR_004802384.1	-0.154232466	0.273191544	-0.87843079	0.346983256	2.931963696	0.033925376	-0.11626284	0.150147583	-0.799977187	0.279794209
XR_004802389.1	-1.093467861	0.007666498	0.150990139	0.750526324	-0.8662882	0.237107732	-0.48634581	0.146855332	-2.327064841	0.050556027
XR_004802397.1	-0.084900465	0.255746192	-0.40061972	0.125021915	2.129581865	0.013765874	0.798699405	0.01289198	-0.441142348	0.383301069
XR_004802441.1	0.181882185	0.173442985	-0.33041604	0.139534935	1.612980045	0.008077425	0.171465831	0.280345143	-0.802208325	0.074380253
XR_004802444.1	-0.069490827	0.898585737	-0.47807634	0.566415982	-0.1002807	0.906766584	-0.00864794	0.987169077	-1.627952103	0.024129701
XR_004802498.1	-0.253685347	0.091222319	0.364505536	0.244357513	0.934228865	0.292661355	0.329010407	0.015954686	-1.318906763	0.032233594
XR_004802575.1	0.446718777	0.204443866	0.010217773	0.980850328	1.133565105	0.626778555	1.611206813	0.028347318	1.712322792	0.135118541
XR_004802598.1	0.305944028	0.333664094	0.27575122	0.035359198	3.065236808	0.02179207	0.185635832	0.362359595	-0.152813397	0.812014993
XR_004802599.1	0.404574202	0.028830787	0.122134525	0.644068576	1.19185125	0.334999074	1.25524874	0.004284885	-0.475286097	0.260929788
XR_004802602.1	0.705766463	0.067238902	-2.42255109	0.076370949	0.168397158	0.939997929	2.199766059	0.0021477	-0.32643717	0.802508479
XR_004802604.1	1.400716772	0.010249418	-0.74237131	0.006519351	1.909340662	0.378664951	1.71534538	0.009179886	-	-
XR_004802637.1	-1.113984804	0.000288646	-	-	-	-	-	-	-1.935131845	0.112129524
XR_004802642.1	0.831685876	0.010088417	0.746285428	0.085359864	1.025603308	0.004040983	0.829283313	2.48159E-05	0.895888984	0.004648097
XR_004802646.1	-1.085916338	0.000988647	-0.71407208	0.055192393	-0.52409617	0.492630068	0.522634045	0.099646443	-0.449991262	0.326529676
XR_004802647.1	-1.346852077	0.017586535	1.243240195	0.096121718	2.438584881	0.100542934	-0.02653542	0.883811038	-2.306247828	0.000112655
XR_004802648.1	0.593358312	0.138554016	-0.01283893	0.977564308	-1.94687739	0.044583964	0.925378565	0.010085742	0.209743271	0.729254182
XR_004802796.1	-0.532578987	0.0153	-1.49650575	0.003408591	-0.01837856	0.988686814	-0.4603735	0.250973766	-0.96160803	0.138634244
XR_004802808.1	1.402615873	0.280611614	-1.87234972	0.435527996	5.840220161	0.015501144	1.338818973	0.224022581	0.636518697	0.770322678

Table S2. The location and correlation between lncRNAs and cis-target mRNAs

lncRNA	partnerRNA	direction	type	distance	subtype	location	correlation	p value	partnerRNA information					
									chromo-some	start	end	strand	geneID	gene description
MSTRG.44144.1	XM_018962090.2	sense	intergenic	23886	same_strand	upstream	-0.052088207	0.78457531	chr12	9469494	9483059	-	108988749	MLO-like protein 1
MSTRG.29440.1	XM_018995231.2	sense	intergenic	28730	same_strand	upstream	-0.209030668	0.2676196	chr11	4781210	4782992	-	109013219	transcription factor bHLH93-like
MSTRG.90641.1	XM_018971298.2	sense	intergenic	1092	same_strand	upstream	-0.291095775	0.11860274	chr16	27478632	27479299	+	108995684	basic form of pathogenesis-related protein 1-like
MSTRG.90641.1	XM_018971357.2	sense	intergenic	5530	same_strand	downstream	-0.07010236	0.71279531	chr16	27466500	27469614	+	108995739	uncharacterized LOC108995739
MSTRG.90641.1	XM_018971370.2	antisense	intergenic	1347	divergent	upstream	0.174483248	0.35643626	chr16	27469998	27473797	-	108995747	DUF21 domain-containing protein At2g14520-like
XR_001999938.2	XM_018984024.2	antisense	intergenic	6015	convergent	downstream	-0.157249823	0.40660864	chr11	36605007	36605666	-	109005210	probable steroid-binding protein 3
XR_004797755.1	XM_018973930.2	antisense	intergenic	14163	convergent	downstream	-0.044257156	0.81636732	chr12	2238300	2244431	+	108997580	G-box-binding factor 1
XR_004797755.1	XM_018973927.2	antisense	intergenic	14163	convergent	downstream	-0.287293506	0.12371477	chr12	2238294	2244431	+	108997580	G-box-binding factor 1
XR_004797755.1	XM_018973928.2	antisense	intergenic	14163	convergent	downstream	-0.033188314	0.86178415	chr12	2238294	2244431	+	108997580	G-box-binding factor 1
XR_004797755.1	XM_018973929.2	antisense	intergenic	14163	convergent	downstream	-0.391421215	0.03243711	chr12	2238298	2244431	+	108997580	G-box-binding factor 1
XR_001999980.2	XM_018998225.2	sense	intergenic	1396	same_strand	upstream	0.51364113	0.0036943	chr16	21936441	21937402	-	109015772	uncharacterized LOC109015772
XR_001999980.2	XM_018953921.2	sense	intergenic	9661	same_strand	upstream	0.313159564	0.09197945	chr16	21914128	21929137	-	108982520	endoplasmic reticulum metallopeptidase 1-like
XR_001999980.2	XM_018997079.2	antisense	intergenic	9647	divergent	upstream	0.355503137	0.05386201	chr16	21951160	21958362	+	109014566	mitochondrial import receptor subunit TOM20-like
XR_001999980.2	XM_018953914.2	antisense	intergenic	2685	divergent	upstream	0.632119751	0.00017883	chr16	21944198	21949067	+	108982514	65-kDa microtubule-associated protein 8-like
MSTRG.80090.1	XM_018952021.2	sense	intergenic	12692	same_strand	downstream	0.489272368	0.00607064	chr15	16306774	16308141	+	108980956	myb-related protein 308-like
MSTRG.65438.1	XM_018993868.2	antisense	intergenic	1388	convergent	downstream	0.29186854	0.11758321	chr14	1342131	1345747	+	109012307	thioredoxin-like protein CITRX, chloroplastic
XR_004801500.1	XM_018964018.2	antisense	intergenic	4078	divergent	upstream	0.113749103	0.54950381	chr5	2006737	2013478	+	108990134	COP9 signalosome complex subunit 7-like
XR_004801500.1	XM_018963972.2	sense	intergenic	6661	same_strand	upstream	0.15327035	0.41873418	chr5	1985690	1991091	-	108990108	nitrate regulatory gene2 protein
MSTRG.72988.1	XM_018977644.2	antisense	intergenic	2889	convergent	downstream	0.20351797	0.28072717	chr14	22550135	22551903	+	109000681	arogenate dehydratase/prephenate dehydratase 6, chloroplastic-like
XR_004801324.1	XM_018970648.2	antisense	intergenic	8470	convergent	downstream	0.104872871	0.58127084	chr4	1674621	1678207	+	108995150	serine/threonine-protein kinase STY13-like
XR_001998322.2	XM_035688440.1	sense	intergenic	270	same_strand	downstream	0.071080188	0.70896089	chr3	3321304	3330055	+	109004642	protein SEMI-ROLLED LEAF 2
XR_001998322.2	XM_018983271.2	sense	intergenic	270	same_strand	downstream	-0.253216177	0.17697937	chr3	3317761	3330055	+	109004642	protein SEMI-ROLLED LEAF 2
XR_001996047.2	XM_018964841.2	sense	intergenic	1616	same_strand	downstream	0.481227663	0.0070976	chr5	2745632	2749576	-	108990765	vesicle-associated protein 1-3-like
XR_001996047.2	XM_018964815.2	antisense	intergenic	6183	convergent	downstream	-0.273709769	0.14330727	chr5	2730505	2735452	+	108990740	autophagy-related protein 8f-like
MSTRG.149637.1	XM_035692311.1	antisense	intergenic	3058	divergent	upstream	-0.040273383	0.8326541	chr7	13981707	14004727	+	109018657	putative acyl-activating enzyme 19
MSTRG.160193.1	XM_035692113.1	sense	intergenic	4858	same_strand	downstream	-0.244771841	0.19235658	chr7	48636014	48640331	-	109013856	protein FLOWERING LOCUS D-like
MSTRG.160193.1	XM_018981368.2	antisense	intergenic	6465	convergent	downstream	-0.386203069	0.03503036	chr7	48620123	48623909	+	109003284	serine carboxypeptidase-like
XR_001996793.2	XM_018970470.2	sense	intergenic	8826	same_strand	downstream	0.372922055	0.0423904	chr7	35377926	35382439	-	108995010	probable 1-deoxy-D-xylulose-5-phosphate synthase, chloroplastic
XR_004798464.1	XM_018964364.2	antisense	intergenic	3397	convergent	downstream	-0.114079932	0.54833565	chr15	4833505	4834638	+	108990403	ethylene-responsive transcription factor RAP2-1-like
XR_004801052.1	XM_018949737.2	sense	intergenic	8598	same_strand	downstream	-0.204265078	0.27892705	chr3	5134345	5136464	-	108979139	polyphenol oxidase, chloroplastic-like
MSTRG.156938.11	XM_035691966.1	sense	intergenic	2296	same_strand	downstream	0.07686112	0.68643668	chr7	39211705	39216390	-	109007173	probable methyltransferase PMT3
MSTRG.156938.11	XM_018986742.2	sense	intergenic	2237	same_strand	downstream	0.485778339	0.00649997	chr7	39211646	39216720	-	109007173	probable methyltransferase PMT3
XR_004798525.1	XM_018986046.2	sense	intergenic	3080	same_strand	downstream	-0.288883412	0.12155778	chr16	23102829	23122246	+	109006684	phototropin-2-like
XR_004801980.1	XM_018974893.2	sense	intergenic	1334	same_strand	upstream	0.116729917	0.5390206	chr7	46339873	46342225	+	108998357	uncharacterized LOC108998357
XR_004801980.1	XM_018995100.2	sense	intergenic	6573	same_strand	downstream	0.037466913	0.84416906	chr7	46322829	46324223	+	109013122	26.5 kDa heat shock protein, mitochondrial-like
MSTRG.115465.1	XM_018958866.1	antisense	intergenic	450	convergent	downstream	0.091779459	0.6295526	chr3	34401934	34406663	+	108986289	elongation factor P
MSTRG.115465.1	XM_018958865.2	antisense	intergenic	384	divergent	upstream	-0.020271908	0.91532329	chr3	34409380	34415251	+	108986288	uncharacterized LOC108986288
XR_001999180.2	XM_018999773.2	sense	intergenic	7820	same_strand	upstream	0.281955705	0.13116302	chr13	4788319	4792323	+	109017528	B3 domain-containing transcription factor VRN1-like
XR_002000952.2	XM_035689406.1	sense	intergenic	1448	same_strand	downstream	-0.28411874	0.12810616	chr1	2480748	2494078	-	109006352	CSC1-like protein HYP1
MSTRG.85040.3	XM_018953257.2	antisense	intergenic	13694	divergent	upstream	-0.146052063	0.4412335	chr16	11100625	11101569	+	108981995	uncharacterized LOC108981995
MSTRG.38815.2	XM_035682957.1	sense	intergenic	6621	same_strand	downstream	-0.338753037	0.06707747	chr11	32312773	32316580	+	108998919	uncharacterized LOC108998919
MSTRG.38815.2	XM_018975485.2	sense	intergenic	8311	same_strand	upstream	0.276011139	0.13983889	chr11	32333235	32334388	+	108998786	transcription factor IBH1-like 1
MSTRG.38815.2	XM_018975689.2	sense	intergenic	6961	same_strand	downstream	-0.463910693	0.00981604	chr11	32312773	32316240	+	108998919	uncharacterized LOC108998919
XR_001994662.2	XM_018953441.2	antisense	intergenic	470	convergent	downstream	-0.259601872	0.16594083	chr12	8628591	8632087	+	108982144	metal tolerance protein 1-like
XR_001997689.2	XM_035693833.1	antisense	intergenic	4070	convergent	downstream	-0.078840006	0.6787853	chr9	10738604	10739605	+	118349419	protein FAR-RED IMPAIRED RESPONSE 1-like
XR_001999397.2	XM_018993358.2	sense	intergenic	2977	same_strand	upstream	0.293994541	0.11481187	chr3	2619488	2621952	+	109011948	probable L-type lectin-domain containing receptor kinase S.5
XR_001996875.2	XM_018970990.1	antisense	intergenic	13271	divergent	upstream	0.189799207	0.31510429	chr2	15395754	15396119	+	108995413	glycine-rich protein DOT1-like
XR_004801727.1	XM_018984324.2	antisense	intergenic	5726	divergent	upstream	0.175777897	0.35282282	chr6	2396166	2398856	+	109005394	glycolipid transfer protein 3-like
XR_002000928.1	XM_019002878.2	sense	intergenic	7037	same_strand	upstream	-0.333973249	0.0712769	chr15	10228620	10233626	+	109020423	PHD finger protein ALFIN-LIKE 4
XR_004798006.1	XM_018955700.2	sense	intergenic	9814	same_strand	downstream	0.347551079	0.05985327	chr13	2599314	2608969	+	108983908	methylmalonate-semialdehyde dehydrogenase [acylating], mitochondrial-like
XR_004798006.1	XM_035684522.1	sense	intergenic	9814	same_strand	downstream	0.181772874	0.33637776	chr13	2599315	2608969	+	108983908	methylmalonate-semialdehyde dehydrogenase [acylating], mitochondrial-like

XR_004798006.1	XM_035684520.1	sense	intergenic	9814	same_strand	downstream	-0.16845548	0.37354853	chr13	2599315	2608969	+	108983908	methylmalonate-semialdehyde dehydrogenase [acylating], mitochondrial-like
XR_004798006.1	XM_018955686.2	antisense	intergenic	9144	convergent	downstream	0.315007145	0.08997556	chr13	2628913	2630572	-	108983902	anthocyanidin 3-O-glucosyltransferase 7-like
MSTRG.80090.2	XM_018952021.2	sense	intergenic	12769	same_strand	downstream	0.157377057	0.40622426	chr15	16306774	16308141	+	108980956	myb-related protein 308-like
XR_004798073.1	XM_018985154.2	sense	intergenic	7297	same_strand	upstream	0.234874563	0.21153212	chr1	15965093	15967008	-	109006018	uncharacterized LOC109006018
XR_004798073.1	XM_035684830.1	sense	intergenic	7519	same_strand	upstream	0.425886991	0.01894823	chr1	15965080	15966786	-	109006018	uncharacterized LOC109006018
XR_001995256.2	XM_018958253.2	antisense	intergenic	244	convergent	downstream	0.475127153	0.00797135	chr1	285002	287016	-	108985820	tubulin alpha chain-like
XR_001995256.2	XM_018958246.2	sense	intergenic	942	same_strand	downstream	-0.23000414	0.22143202	chr1	279480	281965	+	108985812	40S ribosomal protein S9-2
MSTRG.9417.1	XM_018953071.2	antisense	intergenic	7266	convergent	downstream	-0.220352123	0.24196669	chr1	26527078	26558340	-	108981819	phosphoenolpyruvate carboxylase 2
XR_004800813.1	XM_035687181.1	sense	intergenic	4892	same_strand	upstream	0.235405193	0.21047211	chr2	37186509	37201884	+	108985410	E3 ubiquitin-protein ligase UPL1-like
XR_004801510.1	XM_019004149.2	antisense	intergenic	6074	divergent	upstream	0.134316128	0.47917125	chr5	5314230	5315598	+	109021507	uncharacterized LOC109021507
XR_004801510.1	XM_019004147.2	sense	intergenic	1140	same_strand	upstream	0.30170992	0.1051616	chr5	5299022	5303185	-	109021506	mediator of RNA polymerase II transcription subunit 19a-like
XR_004801510.1	XM_035690387.1	sense	intergenic	1140	same_strand	upstream	0.078202719	0.68124601	chr5	5297475	5303185	-	109021506	mediator of RNA polymerase II transcription subunit 19a-like
XR_004801510.1	XM_035690388.1	sense	intergenic	1140	same_strand	upstream	0.124018843	0.51379259	chr5	5297475	5303185	-	109021506	mediator of RNA polymerase II transcription subunit 19a-like
MSTRG.165708.2	XM_035692996.1	antisense	intergenic	212	divergent	upstream	0.357500189	0.05243437	chr8	11116663	11117613	+	118349242	uncharacterized LOC118349242
MSTRG.34095.1	XM_018973469.2	antisense	intergenic	8096	divergent	upstream	-0.223059164	0.23608403	chr11	19005798	19021253	+	108997273	gamma-tubulin complex component 2
MSTRG.96942.1	XM_018963699.2	antisense	intergenic	4057	convergent	downstream	-0.211210784	0.26254754	chr2	18074947	18079022	+	108989931	transcription factor KUA1-like
MSTRG.96942.1	XM_018963700.2	antisense	intergenic	4057	convergent	downstream	-0.07067011	0.71056811	chr2	18074959	18079022	+	108989931	transcription factor KUA1-like
XR_002000714.2	XM_035692920.1	sense	intergenic	9890	same_strand	upstream	0.278385862	0.13632438	chr8	2315640	2320681	+	108979617	two-on-two hemoglobin-3-like
XR_002000714.2	XM_019001414.2	sense	intergenic	8242	same_strand	downstream	0.095104375	0.61713876	chr8	2286636	2295284	+	109019175	coiled-coil domain-containing protein 18-like
XR_001995084.2	XM_018954833.1	antisense	intergenic	3208	divergent	upstream	0.352491107	0.05607303	chr2	36709073	36719181	-	108983250	G-type lectin S-receptor-like serine/threonine-protein kinase At1g11300
MSTRG.30660.1	XM_018974097.2	sense	intergenic	7373	same_strand	upstream	-0.293893836	0.11494204	chr11	9647774	9660444	-	108997723	calmodulin-lysine N-methyltransferase
MSTRG.102213.1	XM_018994034.2	antisense	intergenic	2727	divergent	upstream	0.515421379	0.00355757	chr2	33677910	33682033	-	109012412	uncharacterized LOC109012412
MSTRG.102213.1	XM_018994189.2	sense	intergenic	5839	same_strand	upstream	-0.069474819	0.71525976	chr2	33692313	33695852	+	118343679	UBP1-associated protein 2C-like
MSTRG.76260.2	XM_018964275.2	sense	intergenic	3108	same_strand	upstream	0.966479516	4.51E-18	chr15	4345961	4348163	-	108990335	probable isoaspartyl peptidase/L-asparaginase 2
MSTRG.76260.2	XM_018964385.2	sense	intergenic	8749	same_strand	downstream	-0.209736334	0.26597094	chr15	4363091	4365044	-	108990418	cyclin-D3-1-like
MSTRG.115446.1	XM_018958866.1	antisense	intergenic	11198	divergent	upstream	0.238244122	0.20486266	chr3	34401934	34406663	+	108986289	elongation factor P
MSTRG.115446.1	XM_018958865.2	antisense	intergenic	18644	divergent	upstream	-0.068322321	0.71979309	chr3	34409380	34415251	+	108986288	uncharacterized LOC108986288
XR_004801128.1	XM_018956713.2	antisense	intergenic	5975	convergent	downstream	-0.160149768	0.3978987	chr3	9456278	9460441	-	108984677	uncharacterized protein DDB_G0285917-like
MSTRG.119959.1	XM_018967186.2	sense	intergenic	1222	same_strand	downstream	-0.459521095	0.01062981	chr4	12253204	12255387	-	108992589	60S ribosomal protein L4
XR_001997009.2	XM_018972386.2	antisense	intergenic	402	convergent	downstream	0.189852969	0.31496468	chr7	10114821	10118551	+	108996466	protein trichome birefringence-like 25
MSTRG.148734.1	XM_018962794.1	sense	intergenic	9301	same_strand	upstream	0.28167968	0.13155691	chr7	11299806	11301761	+	108989249	dof zinc finger protein DOF2.5-like
MSTRG.112953.5	XM_035688217.1	antisense	intergenic	8144	convergent	downstream	0.549487059	0.00166023	chr3	27759990	27764140	-	118347920	putative E3 ubiquitin-protein ligase RING1a
MSTRG.112953.5	XM_019003309.2	sense	intergenic	6132	same_strand	upstream	-0.315408971	0.08954417	chr3	27757978	27759285	+	109020786	auxin-repressed 12.5 kDa protein-like
MSTRG.112953.5	XM_035688211.1	sense	intergenic	3861	same_strand	downstream	0.026338364	0.89011793	chr3	27734432	27738995	+	109018462	phosphatidylinositol N-acetylglucosaminyltransferase subunit C-like
XR_001994839.2	XM_018954888.2	sense	intergenic	2623	same_strand	downstream	-0.018827839	0.92133601	chr3	7832092	7835760	-	109011543	putative receptor-like protein kinase At4g00960
XR_001999565.2	XM_018995231.2	antisense	intergenic	36319	divergent	upstream	-0.375265526	0.04100892	chr11	4781210	4782992	-	109013219	transcription factor bHLH93-like
XR_001999698.2	XM_035687456.1	antisense	intergenic	1836	divergent	upstream	0.430632507	0.01752243	chr2	35081152	35084091	+	118343687	G-type lectin S-receptor-like serine/threonine-protein kinase At4g27290
XR_001999698.2	XM_018996532.2	sense	intergenic	9708	same_strand	downstream	0.211016207	0.26299766	chr2	35089024	35092878	-	109014170	G-type lectin S-receptor-like serine/threonine-protein kinase At4g27290
MSTRG.120681.2	XM_018960160.2	sense	intergenic	9796	same_strand	upstream	-0.203443626	0.2809067	chr4	13879628	13880467	-	108987268	uncharacterized LOC108987268
XR_001998246.2	XM_019002058.2	sense	intergenic	17835	same_strand	upstream	0.725974383	5.61E-06	chr10	14694502	14697504	-	109019709	E3 ubiquitin-protein ligase RMA1H1-like
MSTRG.64623.1	XM_035683917.1	sense	intergenic	2866	same_strand	upstream	-0.574534105	0.00089877	chr13	39222172	39224174	-	109010844	UDP-glycosyltransferase 88A1-like
XR_004801412.1	XM_018959205.2	sense	intergenic	51206	same_strand	downstream	-0.010553447	0.95586054	chr4	18503467	18506970	+	118343654	uncharacterized LOC118343654
XR_001994919.2	XM_018955444.2	sense	intergenic	6603	same_strand	downstream	-0.116529845	0.53972125	chr11	26442227	26444611	-	108983711	two-pore potassium channel 5
XR_001995963.2	XM_018964347.2	sense	intergenic	4553	same_strand	upstream	-0.675805591	4.16E-05	chr15	4445342	4446848	+	118343681	uncharacterized LOC118343681
XR_001995963.2	XM_018964277.2	sense	intergenic	9807	same_strand	downstream	-0.407380553	0.02545458	chr15	4427280	4429293	+	108990337	uncharacterized LOC108990337
MSTRG.99248.1	XM_018962021.2	antisense	intergenic	18731	divergent	upstream	-0.188841875	0.31759676	chr2	25164410	25167131	-	108988695	transcription factor MYC2
XR_004798360.1	XM_035687726.1	antisense	intergenic	6574	convergent	downstream	0.382134607	0.03716616	chr2	28031683	28033830	-	108988305	protein FAR-RED IMPAIRED RESPONSE 1-like
MSTRG.142569.1	XM_018986272.2	antisense	intergenic	32339	convergent	downstream	0.609208148	0.00035287	chr6	30895201	30904155	-	109006860	uncharacterized LOC109006860
MSTRG.105423.1	XM_018988119.2	antisense	intergenic	3169	divergent	upstream	-0.144431248	0.44637395	chr3	4795968	4807720	-	109008132	4-alpha-glucanotransferase DPE2
XR_001999031.2	XM_018989657.2	antisense	intergenic	1578	convergent	downstream	-0.157221489	0.40669427	chr7	41716839	41722768	-	109009221	ankyrin repeat protein SKIP35-like
XR_001999031.2	XM_035691938.1	sense	intergenic	5055	same_strand	downstream	0.260738657	0.16402828	chr7	41704697	41708386	+	109009381	uncharacterized LOC109009381
MSTRG.34441.1	XM_019000868.2	sense	intergenic	8628	same_strand	upstream	-0.252779686	0.17775233	chr11	20785560	20789053	-	109018702	probable protein phosphatase 2C 51
MSTRG.34441.1	XM_019000882.2	sense	intergenic	8524	same_strand	upstream	-0.140561388	0.4587766	chr11	20785560	20789157	-	109018702	probable protein phosphatase 2C 51
XR_004798071.1	XM_019000235.2	antisense	intergenic	6905	convergent	downstream	-0.266702901	0.15425074	chr13	38126587	38130300	-	109018043	GDSL esterase/lipase At4g10955-like
XR_004798071.1	XM_019000232.2	antisense	intergenic	6905	convergent	downstream	0.006599639	0.97238911	chr13	38126587	38130283	-	109018043	GDSL esterase/lipase At4g10955-like
MSTRG.165253.1	XM_035693539.1	antisense	genic	0	nested	intronic	-0.105168292	0.58020064	chr8	9882922	9895812	+	108993698	DUF724 domain-containing protein 2-like

MSTRG.165253.1	XM_018968686.2	antisense	genic	0	nested	intronic	-0.08517597	0.65450015	chr8	9882922	9895812	+	108993698	DUF724 domain-containing protein 2-like
XR_004801273.1	XM_018987415.2	sense	intergenic	1464	same_strand	upstream	-0.066396706	0.72738821	chr4	3047822	3050712	+	109007653	uncharacterized LOC109007653
XR_004801273.1	XM_035689570.1	sense	intergenic	3010	same_strand	downstream	-0.312342993	0.09287583	chr4	3030561	3037094	+	109007652	transcription factor LHW-like
XR_001999026.2	XM_018989738.2	antisense	intergenic	133	divergent	upstream	0.367902082	0.04547487	chr7	41668455	41677110	-	109009299	4-hydroxy-tetrahydrodipicolinate reductase 2, chloroplastic-like
XR_004801297.1	XM_018974313.2	sense	intergenic	568	same_strand	downstream	0.343581142	0.06303355	chr4	11563344	11568486	-	108997932	QWRF motif-containing protein 3-like
XR_004801297.1	XM_035689747.1	sense	intergenic	2616	same_strand	downstream	0.464858726	0.00964736	chr4	11565392	11568486	-	108997932	QWRF motif-containing protein 3-like
XR_004801297.1	XM_018974314.2	sense	intergenic	568	same_strand	downstream	0.512739752	0.00376523	chr4	11563344	11568486	-	108997932	QWRF motif-containing protein 3-like
MSTRG.159553.1	XM_019002600.2	sense	intergenic	3651	same_strand	downstream	0.316409571	0.08847679	chr7	46445836	46446785	+	109020177	cucumber peeling cupredoxin-like
XR_001998586.2	XM_018985270.2	sense	intergenic	8175	same_strand	upstream	0.137405422	0.46902481	chr12	27418727	27423913	+	109006104	nitrate regulatory gene2 protein-like
XR_001998586.2	XM_018985287.2	antisense	intergenic	7891	divergent	upstream	0.826945451	1.78E-08	chr12	27398200	27401549	-	109006113	G-type lectin S-receptor-like serine/threonine-protein kinase CES101
MSTRG.142918.1	XM_019000247.2	antisense	intergenic	14934	convergent	downstream	0.038654227	0.83929352	chr6	32044891	32045980	-	109018056	probable calcium-binding protein CML27
MSTRG.142918.1	XM_018991560.2	antisense	intergenic	15951	divergent	upstream	-0.276766059	0.13871456	chr6	32008471	32013131	-	109010664	putative wall-associated receptor kinase-like 16
MSTRG.2520.1	XM_018961358.2	antisense	intergenic	9313	divergent	upstream	0.316852201	0.08800772	chr1	7620045	7628560	+	108988197	ABC transporter B family member 27-like
MSTRG.80051.1	XM_018958266.2	antisense	intergenic	35463	divergent	upstream	-0.198685806	0.2925497	chr15	15963332	16009293	+	108985831	WD repeat-containing protein 26 homolog
MSTRG.80051.1	XM_035686027.1	antisense	intergenic	35496	divergent	upstream	-0.1036443	0.58573086	chr15	15963365	16009293	+	108985831	WD repeat-containing protein 26 homolog
MSTRG.80051.1	XM_018958263.2	antisense	intergenic	35496	divergent	upstream	-0.142386685	0.45290403	chr15	15963365	16009293	+	108985831	WD repeat-containing protein 26 homolog
XR_001997297.2	XM_018995785.2	sense	intergenic	8958	same_strand	downstream	0.259328655	0.16640285	chr10	2978460	2980229	-	109013640	30S ribosomal protein S21, chloroplastic-like
XR_001997476.2	XM_035688718.1	sense	intergenic	41934	same_strand	downstream	-0.088580926	0.64158874	chr3	20832248	20833072	-	118348001	uncharacterized LOC118348001
XR_004802196.1	XM_018974346.2	antisense	intergenic	1134	convergent	downstream	-0.214228868	0.25563016	chr8	11881071	11887322	+	108997958	potassium transporter 5-like
MSTRG.144914.1	XM_018953896.2	antisense	intergenic	4026	divergent	upstream	-0.558696029	0.00133229	chr6	38752675	38759850	+	108982496	chaperone protein ClpD, chloroplastic
MSTRG.144914.1	XM_018953905.2	antisense	intergenic	4025	divergent	upstream	-0.231280038	0.21880876	chr6	38752674	38759850	+	108982496	chaperone protein ClpD, chloroplastic
XR_004797737.1	XM_019005187.2	sense	intergenic	5178	same_strand	upstream	0.085361651	0.65379349	chr12	25971469	25974098	+	109022314	trimethyltridecatetraene synthase-like
MSTRG.76841.1	XM_035685993.1	antisense	intergenic	4473	convergent	downstream	-0.099309689	0.60158512	chr15	6015703	6018510	-	108992321	patatin-like protein 2
MSTRG.136961.1	XM_018951993.2	sense	intergenic	21545	same_strand	upstream	0.031130545	0.87027977	chr6	11260952	11265906	+	108980934	wall-associated receptor kinase-like 14
MSTRG.9873.1	XM_018987170.2	antisense	intergenic	11507	convergent	downstream	-0.242319459	0.19699116	chr1	30674186	30690270	+	109007490	peroxisome biogenesis protein 6
MSTRG.102091.1	XM_035687868.1	sense	intergenic	2052	same_strand	downstream	0.252442539	0.17835099	chr2	33431071	33435134	-	109012090	syntaxin-61-like
MSTRG.102091.1	XM_018993529.2	antisense	intergenic	4591	convergent	downstream	0.27233589	0.14540733	chr2	33417354	33422971	+	109012073	protein GRAVITROPIC IN THE LIGHT 1
MSTRG.102091.1	XM_018993546.2	antisense	intergenic	4591	convergent	downstream	-0.194349221	0.30342474	chr2	33417497	33422971	+	109012073	protein GRAVITROPIC IN THE LIGHT 1
MSTRG.102091.1	XM_018993555.2	antisense	intergenic	4591	convergent	downstream	-0.037894497	0.84241259	chr2	33417395	33422971	+	109012073	protein GRAVITROPIC IN THE LIGHT 1
XR_004801874.1	XM_018996109.2	sense	intergenic	621	same_strand	downstream	-0.4307533	0.01748733	chr7	1023081	1024482	+	109013875	probable esterase D14L
XR_001996920.2	XM_018971847.2	sense	intergenic	411	same_strand	downstream	-0.00421386	0.98236851	chr2	13771689	13778853	-	108996056	cold-responsive protein kinase 1
MSTRG.76158.1	XM_018990618.2	antisense	intergenic	4483	convergent	downstream	0.219893327	0.24297327	chr15	4004212	4006011	+	109009950	blue copper protein-like
MSTRG.102138.1	XM_018993680.2	sense	intergenic	8221	same_strand	upstream	-0.117583016	0.53603788	chr2	33462844	33463588	+	109012164	uncharacterized LOC109012164
MSTRG.102138.1	XM_018993629.2	sense	intergenic	6412	same_strand	downstream	-0.067385949	0.72348317	chr2	33444549	33447558	+	109012125	ras-related protein RABA1b-like
XR_004802266.1	XM_018980688.2	antisense	intergenic	1095	convergent	downstream	0.21118327	0.26261116	chr8	1118528	1119375	+	109002793	14 kDa proline-rich protein DC2.15-like
XR_004802266.1	XM_018957336.2	sense	intergenic	7074	same_strand	downstream	0.150449324	0.42745028	chr8	1128136	1131923	-	108985149	E3 ubiquitin-protein ligase KEG-like
XR_004802266.1	XM_018957335.2	sense	intergenic	7074	same_strand	downstream	0.222876671	0.23647757	chr8	1128136	1131957	-	108985149	E3 ubiquitin-protein ligase KEG-like
XR_004802266.1	XM_018957334.2	sense	intergenic	7074	same_strand	downstream	0.144225018	0.44703032	chr8	1128136	1132318	-	108985149	E3 ubiquitin-protein ligase KEG-like
MSTRG.11836.1	XM_018988950.2	sense	intergenic	9774	same_strand	upstream	-0.117415355	0.53662346	chr1	35164369	35165884	-	109008735	transcription factor MYB1-like
XR_004801159.1	XM_018970292.2	sense	intergenic	2491	same_strand	downstream	0.672788867	4.64E-05	chr3	32437960	32439555	-	108994894	lactosylceramide 4-alpha-galactosyltransferase-like
XR_004801159.1	XM_018952074.2	sense	intergenic	3861	same_strand	upstream	-0.045545218	0.81111702	chr3	32428359	32429547	-	108981006	lactosylceramide 4-alpha-galactosyltransferase-like
XR_001999707.2	XM_018996746.2	sense	intergenic	1667	same_strand	downstream	0.371937441	0.04298182	chr1	20174101	20178870	+	109014318	uncharacterized LOC109014318
MSTRG.36126.1	XM_018969223.2	antisense	intergenic	7489	divergent	upstream	-0.282016061	0.131077	chr11	24923758	24932303	-	108994129	sucrose synthase 2
XR_004797728.1	XM_035683296.1	sense	intergenic	8685	same_strand	upstream	-0.160988734	0.39539885	chr12	23561258	23562882	-	118343945	glycine-rich cell wall structural protein-like
MSTRG.85473.1	XM_018973529.2	sense	genic	0	nested	intronic	0.207744442	0.27064167	chr16	12762851	12782074	+	108997312	trihelix transcription factor GT-1-like
XR_001997677.2	XM_018976980.2	antisense	intergenic	37790	divergent	upstream	0.219532504	0.24376686	chr2	20748327	20749326	+	109000150	lachrymatory-factor synthase-like
XR_004798428.1	XM_018993748.2	antisense	intergenic	190	divergent	upstream	0.423074778	0.01983748	chr15	12207412	12211092	-	109012216	uncharacterized LOC109012216
XR_004798428.1	XM_018993749.2	antisense	intergenic	189	divergent	upstream	-0.254164734	0.1753078	chr15	12207412	12211093	-	109012216	uncharacterized LOC109012216
MSTRG.140367.1	XM_018974825.2	antisense	intergenic	201	divergent	upstream	-0.199659482	0.29014243	chr6	23085923	23087566	-	108998302	glucan endo-1,3-beta-glucosidase-like
MSTRG.168917.1	XM_035692825.1	sense	intergenic	8454	same_strand	downstream	0.211536498	0.26179519	chr8	23653424	23654248	+	108990532	protein FD-like
XR_001996351.2	XM_018967207.2	antisense	intergenic	1858	divergent	upstream	0.007622354	0.96811239	chr4	12128377	12132676	-	108992606	probable aquaporin SIP2-1
XR_001999476.2	XM_018994296.2	antisense	intergenic	5875	divergent	upstream	0.246248552	0.18960269	chr13	35946675	35949440	-	109012584	F-box/kelch-repeat protein At1g22040
MSTRG.24321.16	XM_018997964.2	sense	intergenic	81150	same_strand	upstream	-0.094678254	0.61872405	chr10	26244094	26247277	-	109015490	IQ domain-containing protein IQM1-like
XR_002000950.2	XM_035689406.1	sense	intergenic	16395	same_strand	downstream	-0.044443828	0.81560594	chr1	2480748	2494078	-	109006352	CSC1-like protein HYP1
MSTRG.136219.1	XM_018958212.2	antisense	intergenic	4213	convergent	downstream	0.127860395	0.5007345	chr6	8999638	9001832	+	108985789	patatin-like protein 2
XR_001995701.2	XM_018961937.2	antisense	intergenic	144	convergent	downstream	-0.039190609	0.83709284	chr5	661116	664126	+	108988620	bifunctional endo-1,4-beta-xylanase XylA-like

XR_001995701.2	XM_018961975.2	sense	intergenic	1320	same_strand	downstream	-0.204299854	0.27884345	chr5	666671	675300	-	108988645	glycosyltransferase-like KOBITO 1
XR_001995701.2	XM_018961908.2	sense	intergenic	6588	same_strand	upstream	0.28366075	0.12874899	chr5	654771	657682	-	108988594	protein IQ-DOMAIN 14
XR_001995701.2	XM_018961946.2	antisense	intergenic	145	convergent	downstream	0.059243317	0.75582024	chr5	661116	664125	+	108988620	bifunctional endo-1,4-beta-xylanase XylA-like
MSTRG.125772.1	XM_019003274.2	antisense	intergenic	190	convergent	downstream	-0.074782086	0.69450812	chr4	32399405	32405709	+	109020747	DNA polymerase delta small subunit-like
MSTRG.125772.1	XM_035689899.1	sense	intergenic	3127	same_strand	downstream	-0.339178807	0.06671294	chr4	32409937	32413475	-	109015564	actin-1
XR_001996859.2	XM_018971035.2	antisense	intergenic	3949	divergent	upstream	0.738747638	3.14E-06	chr2	6961129	6965322	+	108995459	2-hydroxy-palmitic acid dioxygenase MPO1-like
XR_001999867.2	XM_035694424.1	sense	intergenic	6724	same_strand	downstream	0.69769138	1.82E-05	chr10	31172488	31174152	-	108990991	anthocyanidin 3-O-glucosyltransferase 7-like
XR_004797991.1	XM_018956959.2	sense	intergenic	16605	same_strand	downstream	-0.156596349	0.40858608	chr13	11062693	11065851	-	108984875	probable inactive purple acid phosphatase 2
XR_004797991.1	XM_018953052.2	sense	intergenic	13488	same_strand	upstream	0.027287557	0.88618301	chr13	11023018	11028776	-	108981804	probable WRKY transcription factor 57
XR_004802185.1	XM_018952232.2	sense	intergenic	1202	same_strand	downstream	0.532770169	0.0024371	chr8	6773988	6776886	+	108981148	uncharacterized LOC108981148
XR_004801698.1	XM_035690662.1	antisense	intergenic	41689	convergent	downstream	-0.295881242	0.11239341	chr6	11693661	11694374	+	118348613	uncharacterized LOC118348613
XR_004797931.1	XM_018963464.2	antisense	intergenic	4716	convergent	downstream	0.092795207	0.62574952	chr13	25941726	25945705	+	108989741	BTB/POZ domain-containing protein At1g67900
MSTRG.32003.1	XM_018971422.2	sense	intergenic	10250	same_strand	upstream	-0.284559786	0.12748935	chr11	12062705	12075230	-	108995793	putative receptor-like protein kinase At4g00960
XR_001998763.2	XM_018986965.2	antisense	intergenic	4403	convergent	downstream	0.460242	0.0104924	chr5	19554855	19558615	-	109007335	3'-5' exoribonuclease 1-like
MSTRG.73145.1	XM_018977590.2	sense	intergenic	5883	same_strand	upstream	-0.104084285	0.58413188	chr14	23131408	23132412	+	109000636	RING-H2 finger protein ATL57-like
MSTRG.125329.1	XM_018958581.2	antisense	intergenic	6286	divergent	upstream	-0.089784392	0.63704941	chr4	31084604	31089116	+	108986065	sulfoquinovosidase-like
MSTRG.125329.1	XM_018958580.2	sense	intergenic	2832	same_strand	upstream	0.371952695	0.0429726	chr4	31067114	31069627	-	108986064	F-box protein At2g32560-like
MSTRG.156420.1	XM_018951789.2	sense	intergenic	277	same_strand	downstream	0.281688222	0.13154471	chr7	37730176	37731433	+	108980780	endochitinase EP3-like
XR_004801737.1	XM_018971161.2	sense	intergenic	39540	same_strand	downstream	0.558566481	0.00133648	chr6	12313380	12317093	-	108995573	uncharacterized LOC108995573
XR_004801737.1	XM_018971162.2	sense	intergenic	39576	same_strand	downstream	0.518385079	0.00333959	chr6	12313416	12317117	-	108995573	uncharacterized LOC108995573
MSTRG.175157.1	XM_018969643.2	sense	intergenic	7980	same_strand	upstream	0.5516632	0.001577	chr9	15034472	15037589	+	108994427	monooxygenase 2
MSTRG.111599.5	XM_018977858.2	antisense	intergenic	82419	convergent	downstream	-0.243279524	0.19516769	chr3	22553155	22555614	-	109000849	transcription factor MYB52-like
XR_004797937.1	XM_018994296.2	antisense	intergenic	5871	divergent	upstream	0.598067227	0.00048208	chr13	35946675	35949440	-	109012584	F-box/kelch-repeat protein At1g22040
XR_004798329.1	XM_035687736.1	sense	intergenic	1858	same_strand	downstream	-0.246069612	0.18993492	chr2	23440101	23442596	+	109000255	MATH domain and coiled-coil domain-containing protein At2g05420-like
XR_001999091.2	XM_018990245.2	antisense	intergenic	454	convergent	downstream	0.604697281	0.00040094	chr1	3978995	3983057	+	109009665	uncharacterized LOC109009665
XR_001999091.2	XM_019001875.2	antisense	intergenic	7405	divergent	upstream	0.507510606	0.00420022	chr1	3991996	3994821	+	109019566	protein LOW PHOTOSYNTHETIC EFFICIENCY 1, chloroplastic-like
MSTRG.116423.3	XM_018970673.2	sense	intergenic	1390	same_strand	downstream	0.071582659	0.70699322	chr4	2096832	2101264	+	108995171	26S proteasome non-ATPase regulatory subunit 7 homolog B-like
MSTRG.116423.3	XM_018970672.2	sense	intergenic	1703	same_strand	upstream	-0.022232555	0.90716707	chr4	2107115	2119226	+	108995170	putative tRNA (cytidine(32)/guanosine(34)-2'-O)-methyltransferase
MSTRG.122776.1	XM_018964193.2	antisense	intergenic	347	convergent	downstream	-0.049441391	0.79528543	chr4	23938521	23940511	-	108990276	calcyclin-binding protein-like
MSTRG.122776.1	XM_035689811.1	sense	intergenic	3128	same_strand	downstream	-0.199792662	0.28981414	chr4	23925426	23934352	+	108990268	katanin p60 ATPase-containing subunit A-like 2
XR_001999338.2	XM_018992825.2	antisense	intergenic	9137	divergent	upstream	0.057005787	0.76478012	chr2	31700161	31701184	+	109011566	dehydration-responsive element-binding protein 1A-like
MSTRG.157498.1	XM_018963041.2	antisense	intergenic	8706	divergent	upstream	-0.269248673	0.15020742	chr7	40778002	40781106	+	108989438	putative calcium-transporting ATPase 13, plasma membrane-type
MSTRG.144536.3	XM_035690944.1	antisense	intergenic	7108	convergent	downstream	-0.146687638	0.43922654	chr6	38057985	38060650	-	109005979	transmembrane 9 superfamily member 11
XR_001998880.2	XM_018987897.2	sense	intergenic	18552	same_strand	downstream	0.056394281	0.76723405	chr10	16489399	16490268	+	109007977	uncharacterized LOC109007977
XR_001996832.2	XM_018970882.2	antisense	intergenic	5327	convergent	downstream	-0.370059076	0.04412834	chr7	3680372	3684832	-	108995333	probable protein phosphatase 2C 80
XR_001996832.2	XM_018970925.2	antisense	intergenic	7994	divergent	upstream	0.278302629	0.13644646	chr7	3662416	3664525	-	108995362	scarecrow-like protein 15
XR_001996832.2	XM_018970879.2	antisense	intergenic	5327	convergent	downstream	-0.070481543	0.71130758	chr7	3680372	3684834	-	108995333	probable protein phosphatase 2C 80
XR_001996832.2	XM_018970877.2	antisense	intergenic	5327	convergent	downstream	0.160236371	0.39764023	chr7	3680372	3684834	-	108995333	probable protein phosphatase 2C 80
XR_004802041.1	XM_018966220.2	antisense	intergenic	3963	convergent	downstream	-0.263119412	0.1600738	chr7	51736000	51737193	-	108991827	PRA1 family protein B1-like
XR_004802041.1	XM_018966270.2	sense	intergenic	5046	same_strand	downstream	0.077320827	0.68465648	chr7	51713947	51718390	+	108991872	sorting nexin 2B-like
MSTRG.71506.1	XM_035685058.1	sense	intergenic	237	same_strand	upstream	0.594192106	0.00053592	chr14	18647043	18648850	+	109014024	2-oxoglutarate-dependent dioxygenase DAO-like
XR_001999264.2	XM_018991765.2	antisense	intergenic	119	divergent	upstream	0.239159317	0.20307643	chr13	39326106	39333439	+	109010837	uncharacterized protein At3g49140-like
XR_001999264.2	XM_035684128.1	sense	intergenic	4128	same_strand	upstream	0.145056698	0.44438652	chr13	39316280	39320114	-	109010836	uncharacterized LOC109010836
XR_001999264.2	XM_018991764.2	antisense	intergenic	119	divergent	upstream	-0.06825443	0.72006043	chr13	39326106	39333439	+	109010837	uncharacterized protein At3g49140-like
XR_001999264.2	XM_035684127.1	sense	intergenic	4128	same_strand	upstream	-0.191869687	0.30975536	chr13	39316089	39320114	-	109010836	uncharacterized LOC109010836
MSTRG.3134.1	XM_035694799.1	sense	intergenic	6119	same_strand	downstream	0.013466001	0.94369594	chr1	9091333	9099182	+	108998579	probable transcriptional regulator SLK2
MSTRG.3134.1	XM_018975149.2	sense	intergenic	6119	same_strand	downstream	-0.144647627	0.44568584	chr1	9091324	9099182	+	108998579	probable transcriptional regulator SLK2
MSTRG.3134.1	XM_018975152.2	sense	intergenic	6119	same_strand	downstream	-0.131883504	0.48723988	chr1	9091324	9099182	+	108998579	probable transcriptional regulator SLK2
MSTRG.71734.1	XM_018961482.2	antisense	intergenic	38105	convergent	downstream	-0.14363321	0.44891674	chr14	19139010	19143131	+	108988270	BEL1-like homeodomain protein 1
XR_004801094.1	XM_018995766.2	sense	intergenic	6358	same_strand	upstream	0.341135128	0.06505779	chr3	13700310	13704682	+	109013621	probable LRR receptor-like serine/threonine-protein kinase At3g47570
XR_004798263.1	XM_035685041.1	sense	intergenic	493	same_strand	upstream	-0.021311785	0.91099634	chr14	2592020	2593808	+	109016244	hydroquinone glucosyltransferase-like
MSTRG.37049.1	XM_018992739.2	antisense	intergenic	5000	convergent	downstream	-0.135438561	0.4754717	chr11	27332781	27335888	+	109011493	protein PLASTID MOVEMENT IMPAIRED 1
MSTRG.37049.1	XM_018992743.2	sense	intergenic	7044	same_strand	downstream	-0.300316199	0.1068582	chr11	27348337	27350367	-	109011498	outer envelope pore protein 21, chloroplastic-like
MSTRG.37049.1	XM_035695688.1	sense	intergenic	9435	same_strand	downstream	-0.450893327	0.01239505	chr11	27350728	27357045	-	109011501	dolichyl-diphosphooligosaccharide--protein glycosyltransferase subunit 1A-like
MSTRG.135346.1	XM_018978392.2	antisense	intergenic	4340	convergent	downstream	-0.273659396	0.14338387	chr6	6128655	6131281	+	109001202	uncharacterized LOC109001202
XR_001998005.2	XM_018980103.2	antisense	intergenic	5065	divergent	upstream	-0.119794205	0.52834359	chr14	6777056	6788474	-	109002381	6-phosphofructo-2-kinase/fructose-2,6-bisphosphatase-like

XR_001994663.2	XM_018953441.2	antisense	intergenic	470	convergent	downstream	-0.294075265	0.11470761	chr12	8628591	8632087	+	108982144	metal tolerance protein 1-like
XR_001996083.2	XM_018965065.2	sense	intergenic	6369	same_strand	upstream	0.129764717	0.49432364	chr1	32612059	32612616	+	108990939	uncharacterized LOC108990939
XR_001998948.2	XM_018988632.2	sense	intergenic	9321	same_strand	upstream	-0.509234709	0.00405227	chr14	4695171	4697739	+	109008517	non-functional NADPH-dependent codeinone reductase 2-like
XR_004798497.1	XM_018967924.2	sense	intergenic	9582	same_strand	upstream	0.868529992	4.96E-10	chr15	18567676	18577735	-	108993143	lupeol synthase-like
MSTRG.44148.1	XM_018962090.2	antisense	intergenic	38261	divergent	upstream	0.070768283	0.71018323	chr12	9469494	9483059	-	108988749	MLO-like protein 1
MSTRG.41494.2	XM_018973911.2	sense	intergenic	3263	same_strand	downstream	0.084613392	0.65664298	chr12	2461615	2467119	+	108997572	sulfite exporter TauE/SafE family protein 3-like
XR_001999046.1	XM_035695530.1	sense	intergenic	8018	same_strand	downstream	-0.087965009	0.64391683	chr11	886377	926456	+	109002563	protein MON2 homolog
XR_001999046.1	XM_035695398.1	sense	intergenic	2610	same_strand	downstream	0.030065767	0.87468139	chr11	927853	931864	+	109009349	pre-rRNA-processing protein TSR2-like
XR_001999046.1	XM_018989795.2	sense	intergenic	5738	same_strand	upstream	-0.016019367	0.93304163	chr11	942516	955498	+	109009350	serine/threonine-protein kinase EDR1-like
XR_002000563.2	XM_035682813.1	antisense	intergenic	4142	convergent	downstream	0.063279342	0.73973747	chr11	31328915	31340519	+	108983177	calcium-transporting ATPase 1-like
MSTRG.22685.1	XM_018982234.2	antisense	intergenic	3003	convergent	downstream	0.043123778	0.82099357	chr10	21847482	21855904	+	109003896	U3 snoRNP-associated protein-like EMB2271
XR_004798070.1	XM_018965859.2	sense	intergenic	6138	same_strand	downstream	-0.273307081	0.14392051	chr13	25240111	25244963	-	108991553	E3 ubiquitin-protein ligase RGLG5-like
XR_004798070.1	XM_035684277.1	sense	intergenic	6138	same_strand	downstream	-0.031228716	0.86987413	chr13	25240111	25245231	-	108991553	E3 ubiquitin-protein ligase RGLG5-like
XR_004798070.1	XM_035684275.1	sense	intergenic	6138	same_strand	downstream	-0.191771288	0.31000827	chr13	25240111	25245080	-	108991553	E3 ubiquitin-protein ligase RGLG5-like
XR_001995455.2	XM_018959765.2	sense	intergenic	9776	same_strand	downstream	0.055516292	0.77076123	chr13	5954736	5955359	-	108986951	auxin-responsive protein SAUR21-like
XR_001995455.2	XM_018959761.2	sense	intergenic	2653	same_strand	downstream	-0.315774911	0.08915268	chr13	5947613	5948104	-	108986948	auxin-induced protein X15-like
MSTRG.91921.1	XM_018984537.2	antisense	intergenic	772	convergent	downstream	0.395150932	0.03068056	chr2	2205332	2206144	+	109005543	uncharacterized LOC109005543
MSTRG.91921.1	XM_018984530.2	sense	intergenic	7387	same_strand	downstream	0.145708692	0.44231982	chr2	2215384	2219851	-	109005540	protein JOKA2
MSTRG.21076.1	XM_035695061.1	sense	intergenic	23002	same_strand	upstream	-0.346167534	0.06094701	chr10	15994906	15998768	+	108982169	uncharacterized LOC108982169
MSTRG.144025.1	XM_018963190.2	sense	intergenic	7751	same_strand	upstream	-0.455900397	0.011343	chr6	35256778	35258654	+	108989548	uncharacterized LOC108989548
MSTRG.131507.1	XM_018959106.2	antisense	intergenic	5446	convergent	downstream	-0.332357708	0.07274141	chr5	16816369	16817819	-	108986478	NAC domain-containing protein 90-like
MSTRG.28486.1	XM_018988031.2	sense	intergenic	10639	same_strand	upstream	-0.06856701	0.71882982	chr11	2072644	2078013	+	109008078	probable linoleate 9S-lipoxygenase 5
MSTRG.144045.1	XM_018963185.2	antisense	intergenic	9349	convergent	downstream	-0.200909725	0.28706992	chr6	35214774	35230219	-	108989546	lupeol synthase-like
XR_001994911.2	XM_018993441.2	sense	intergenic	9086	same_strand	upstream	-0.514103489	0.00365836	chr12	30449350	30450149	-	109012015	uncharacterized LOC109012015
XR_004797573.1	XM_018978730.2	sense	intergenic	14448	same_strand	downstream	-0.202717172	0.28266491	chr1	23009277	23011409	+	109001448	uncharacterized protein At4g28440
XR_004797573.1	XM_018978727.2	sense	intergenic	19072	same_strand	downstream	-0.172062808	0.36325072	chr1	23004654	23006785	+	109001447	30S ribosomal protein S9, chloroplastic
MSTRG.2510.1	XM_018961307.2	antisense	intergenic	3055	divergent	upstream	0.862719984	8.76E-10	chr1	7334348	7337598	-	108988161	receptor-like protein kinase FERONIA
XR_001994163.2	XM_018949737.2	sense	intergenic	4241	same_strand	downstream	-0.329710796	0.07519103	chr3	5134345	5136464	-	108979139	polyphenol oxidase, chloroplastic-like
XR_002000661.2	XM_019000986.2	antisense	intergenic	345	convergent	downstream	0.225536251	0.23078554	chr10	3981839	3984463	-	109018802	nudix hydrolase 18, mitochondrial-like
XR_002000661.2	XM_019001860.2	sense	intergenic	2506	same_strand	downstream	0.370829116	0.0436554	chr10	3973764	3977435	+	109019554	ADP-ribosylation factor-like protein 8c
XR_004801774.1	XM_035690843.1	antisense	intergenic	1513	divergent	upstream	-0.312074266	0.09317226	chr6	446871	452151	-	108993261	leucoanthocyanidin reductase-like
XR_004802808.1	XM_018991491.2	antisense	intergenic	1574	divergent	upstream	-0.370540091	0.04383244	chr1	15050988	15054550	+	109010612	probable pectinesterase/pectinesterase inhibitor 51
MSTRG.43488.1	XM_018952387.2	antisense	intergenic	7367	convergent	downstream	0.001517613	0.99364963	chr12	8053815	8054280	-	108981280	auxin-responsive protein SAUR32-like
XR_001996012.2	XM_018964562.2	sense	intergenic	2407	same_strand	upstream	0.36919068	0.04466657	chr7	38540919	38546264	-	108990564	nuclear transport factor 2-like
XR_001999896.2	XM_018995575.2	antisense	intergenic	2971	convergent	downstream	0.106859509	0.57409082	chr4	32784744	32786604	+	109013475	40S ribosomal protein S28
XR_001999896.2	XM_018995571.2	sense	intergenic	5091	same_strand	upstream	0.2617424	0.16235265	chr4	32772014	32784484	-	109013473	homogentisate solanesyltransferase, chloroplastic
XR_004801425.1	XM_018960161.2	antisense	intergenic	9207	divergent	upstream	0.267059892	0.15367908	chr4	14029009	14030601	+	108987269	transcription factor MYB41-like
XR_004798427.1	XM_035687587.1	sense	intergenic	7020	same_strand	downstream	0.461430473	0.01026913	chr2	24187836	24197507	+	108982940	tobamovirus multiplication protein 2A-like
MSTRG.174897.1	XM_018969536.2	antisense	intergenic	5587	divergent	upstream	0.471282634	0.00856743	chr9	14620801	14627525	+	108994370	gamma carbonic anhydrase-like 2, mitochondrial
XR_001998574.1	XM_018985222.2	sense	intergenic	5602	same_strand	downstream	0.253228973	0.17695675	chr1	16652184	16666811	-	109006065	CLIP-associated protein
XR_001998574.1	XM_018985221.2	sense	intergenic	5602	same_strand	downstream	0.256591133	0.17108265	chr1	16652184	16666811	-	109006065	CLIP-associated protein
MSTRG.109627.1	XM_018996023.2	antisense	genic	0	nested	intronic	-0.161946087	0.39255723	chr3	17323951	17379251	+	109013811	probable LRR receptor-like serine/threonine-protein kinase At1g56130
MSTRG.109627.1	XM_035688276.1	antisense	intergenic	9600	divergent	upstream	-0.202514071	0.28315772	chr3	17347299	17379251	+	109013811	probable LRR receptor-like serine/threonine-protein kinase At1g56130
MSTRG.96292.1	XM_018971135.2	antisense	intergenic	1609	convergent	downstream	0.037305398	0.84483274	chr2	16125505	16125897	-	108995552	uncharacterized LOC108995552
MSTRG.22142.2	XM_018959852.2	sense	intergenic	45378	same_strand	upstream	0.149349545	0.43087511	chr10	18767107	18782485	+	108987017	protein MAIN-LIKE 2-like
XR_004798447.1	XM_018975968.2	antisense	intergenic	836	convergent	downstream	-0.145441967	0.44316466	chr2	4055461	4060989	+	108999166	TORTIFOLIA1-like protein 3
XR_004798447.1	XM_018975971.2	sense	intergenic	2431	same_strand	downstream	0.241006702	0.19950354	chr2	4066953	4073863	-	108999167	uncharacterized LOC108999167
XR_004797967.1	XM_018986501.2	sense	intergenic	677	same_strand	downstream	0.400870696	0.0281374	chr13	4052531	4055161	+	109007022	probable receptor-like protein kinase At1g11050
XR_004797936.1	XM_018966405.2	antisense	intergenic	17816	convergent	downstream	-0.814544207	4.31E-08	chr1	19301449	19306160	-	108991977	G-type lectin S-receptor-like serine/threonine-protein kinase At4g03230
XR_004798004.1	XM_018954534.2	antisense	intergenic	8261	convergent	downstream	-0.272865077	0.14459581	chr13	2518311	2526242	-	108983024	MADS-box transcription factor ANR1-like
MSTRG.135106.1	XM_018997031.2	antisense	intergenic	2178	convergent	downstream	-0.451820809	0.01219424	chr6	5644093	5651351	+	109014533	uncharacterized LOC109014533
MSTRG.135106.1	XM_018997030.2	antisense	intergenic	2178	convergent	downstream	0.414476834	0.02277213	chr6	5644087	5651351	+	109014533	uncharacterized LOC109014533
MSTRG.104704.1	XM_018950425.2	antisense	intergenic	2120	divergent	upstream	0.071759939	0.70629943	chr3	2742226	2743899	+	108979703	uncharacterized LOC108979703
MSTRG.104704.1	XM_018950419.2	sense	intergenic	2016	same_strand	upstream	0.579106909	0.00079924	chr3	2713164	2735581	-	108979699	protein PHOTOPERIOD-INDEPENDENT EARLY FLOWERING 1-like
XR_001996377.2	XM_035683491.1	sense	intergenic	13775	same_strand	upstream	0.234278489	0.21272721	chr1	39313674	39333051	+	108992674	casein kinase 1-like protein HD16
MSTRG.59243.1	XM_018996979.2	antisense	intergenic	474	divergent	upstream	-0.188907728	0.31742492	chr13	17799507	17806988	-	109014487	protein MAK16 homolog

MSTRG.820.6	XM_018985572.2	antisense	intergenic	6146	divergent	upstream	0.229089695	0.22332518	chr1	2123724	2131445	-	109006328	O-fucosyltransferase 9
MSTRG.82486.2	XM_018994138.2	sense	intergenic	1138	same_strand	downstream	0.073568902	0.69923331	chr16	3040731	3047211	+	109012474	uncharacterized protein At1g51745
XR_001996945.2	XM_018971741.2	sense	intergenic	1146	same_strand	upstream	0.333674158	0.0715463	chr1	42265528	42274496	+	108996020	U3 small nucleolar RNA-associated protein 18 homolog
MSTRG.167497.1	XM_018952771.2	antisense	intergenic	17799	divergent	upstream	-0.458167109	0.01089197	chr8	17336199	17344037	-	108981553	tyrosine-protein phosphatase DSP5-like
XR_001996394.2	XM_018967366.2	antisense	intergenic	5633	divergent	upstream	0.259585647	0.16596825	chr9	22293809	22300163	-	108992732	uncharacterized LOC108992732
XR_001999335.2	XM_018992739.2	antisense	intergenic	8774	divergent	upstream	0.378778415	0.03900582	chr11	27332781	27335888	+	109011493	protein PLASTID MOVEMENT IMPAIRED 1
MSTRG.34462.8	XM_019000744.2	antisense	intergenic	9386	convergent	downstream	0.356411617	0.05320881	chr11	21205303	21211204	+	109018587	sulfate transporter 3.1-like
XR_001999023.2	XM_035695610.1	sense	intergenic	9701	same_strand	upstream	0.712069073	1.02E-05	chr11	1143066	1153765	+	109005679	sodium/hydrogen exchanger 2-like
XR_001999023.2	XM_018989724.2	sense	intergenic	6695	same_strand	downstream	0.06916043	0.71649547	chr11	1097694	1125750	+	109009288	protein HASTY 1
MSTRG.134173.1	XM_018992629.2	sense	intergenic	3961	same_strand	upstream	-0.155618215	0.41155604	chr6	1885134	1887824	+	109011418	trihelix transcription factor GT-2-like
MSTRG.134173.1	XM_018992628.2	sense	intergenic	3961	same_strand	upstream	-0.059378043	0.75528172	chr6	1885134	1887824	+	109011418	trihelix transcription factor GT-2-like
XR_001994877.2	XM_035687181.1	sense	intergenic	4892	same_strand	upstream	0.30971268	0.09580829	chr2	37186509	37201884	+	108985410	E3 ubiquitin-protein ligase UPL1-like
MSTRG.24322.3	XM_018997964.2	sense	intergenic	56695	same_strand	upstream	0.273117364	0.14421008	chr10	26244094	26247277	-	109015490	IQ domain-containing protein IQM1-like
XR_002000951.2	XM_035689406.1	sense	intergenic	9874	same_strand	downstream	-0.254181505	0.17527834	chr1	2480748	2494078	-	109006352	CSC1-like protein HYP1
XR_001996557.2	XM_018968702.2	antisense	intergenic	1259	convergent	downstream	0.547939037	0.00172174	chr8	10000422	10002334	-	108993709	WRKY transcription factor 23-like
MSTRG.142849.1	XM_018995982.2	antisense	intergenic	608	divergent	upstream	-0.002775714	0.98838545	chr6	31544833	31557365	-	109013779	mitogen-activated protein kinase 9-like
XR_001999832.2	XM_018997560.2	antisense	genic	0	overlapping	exonic	-0.346859118	0.06039835	chr16	21638118	21646738	+	109015070	probable isoprenylcysteine alpha-carbonyl methylesterase ICMEL1
XR_001995437.2	XM_035690662.1	antisense	intergenic	32714	convergent	downstream	-0.285329624	0.12641794	chr6	11693661	11694374	+	118348613	uncharacterized LOC118348613
XR_001998193.2	XM_018982237.2	antisense	intergenic	9972	divergent	upstream	-0.076505417	0.68781526	chr10	21941212	21957939	+	109003899	3-hydroxyisobutyryl-CoA hydrolase-like protein 5
XR_001998193.2	XM_018982239.2	antisense	intergenic	9972	divergent	upstream	0.266063454	0.1552785	chr10	21941212	21957939	+	109003899	3-hydroxyisobutyryl-CoA hydrolase-like protein 5
XR_001995702.2	XM_018961937.2	antisense	intergenic	144	convergent	downstream	0.366451595	0.04639862	chr5	661116	664126	+	108988620	bifunctional endo-1,4-beta-xylanase XylA-like
XR_001995702.2	XM_018961908.2	sense	intergenic	6588	same_strand	upstream	0.483607954	0.00677944	chr5	654771	657682	-	108988594	protein IQ-DOMAIN 14
XR_001995702.2	XM_018961946.2	antisense	intergenic	145	convergent	downstream	-0.276444383	0.13919284	chr5	661116	664125	+	108988620	bifunctional endo-1,4-beta-xylanase XylA-like
XR_001995702.2	XM_018961975.2	sense	intergenic	1320	same_strand	downstream	-0.373705142	0.04192469	chr5	666671	675300	-	108988645	glycosyltransferase-like KOBITO 1
MSTRG.38318.1	XM_019003910.2	antisense	intergenic	4583	convergent	downstream	0.562276241	0.00122095	chr11	31037830	31040125	-	109021300	uncharacterized LOC109021300
MSTRG.89670.1	XM_018984778.2	antisense	intergenic	2603	convergent	downstream	0.388494423	0.03387177	chr16	24728187	24739154	+	109005733	V-type proton ATPase subunit a3-like
MSTRG.40823.1	XM_019003272.2	antisense	intergenic	5980	divergent	upstream	-0.144447872	0.44632107	chr12	865499	866351	+	109020745	F-box protein GID2-like
MSTRG.165141.1	XM_018968694.2	antisense	genic	0	nested	intronic	-0.184705354	0.32850634	chr8	9473887	9493295	+	108993702	proteasome subunit beta type-4-like
MSTRG.165141.1	XM_018968697.2	antisense	intergenic	557	convergent	downstream	0.455088159	0.01150839	chr8	9484298	9484812	+	108993705	uncharacterized LOC108993705
XR_001995964.2	XM_018964277.2	sense	intergenic	9807	same_strand	downstream	-0.397186797	0.02975481	chr15	4427280	4429293	+	108990337	uncharacterized LOC108990337
XR_001995964.2	XM_018964347.2	sense	intergenic	4553	same_strand	upstream	-0.677572943	3.90E-05	chr15	4445342	4446848	+	118343681	uncharacterized LOC118343681
XR_001994779.2	XM_018954387.2	sense	intergenic	2806	same_strand	upstream	-0.16992174	0.36934239	chr7	44278846	44282748	-	108982903	putative F-box/FBD/LRR-repeat protein At1g66290
MSTRG.93970.1	XM_018969282.2	antisense	intergenic	17568	convergent	downstream	0.458929692	0.01074366	chr2	8684152	8686220	+	108994163	homeobox-leucine zipper protein ATHB-21-like
MSTRG.9746.1	XM_035688027.1	antisense	intergenic	11384	convergent	downstream	0.71798871	7.94E-06	chr1	30363637	30367597	+	108979144	protein trichome birefringence-like 43
XR_001997676.2	XM_018976980.2	antisense	intergenic	37754	divergent	upstream	0.017254846	0.92789037	chr2	20748327	20749326	+	109000150	lachrymatory-factor synthase-like
MSTRG.73597.1	XM_018969955.2	antisense	intergenic	5380	convergent	downstream	0.355051345	0.0541892	chr14	24360267	24362754	+	108994657	scarecrow-like protein 3
MSTRG.87209.1	XM_035686752.1	sense	intergenic	24492	same_strand	upstream	-0.23976738	0.20189559	chr16	18273453	18280448	+	108987108	disease resistance protein At4g27190-like
MSTRG.87209.1	XM_035686754.1	sense	intergenic	24492	same_strand	upstream	0.423496268	0.01970205	chr16	18273453	18280861	+	108987108	disease resistance protein At4g27190-like
XR_002000880.2	XM_019002518.2	sense	intergenic	9139	same_strand	upstream	0.325195277	0.0795163	chr4	7661132	7666548	+	109020113	RNA polymerase sigma factor sigF, chloroplastic
MSTRG.52129.1	XM_018972190.2	sense	intergenic	5296	same_strand	upstream	-0.235324211	0.21063365	chr13	1149902	1151809	+	108996343	UDP-glycosyltransferase 84B1-like
MSTRG.67827.1	XM_035685151.1	antisense	intergenic	37422	convergent	downstream	0.127577229	0.50169132	chr14	8648239	8651197	+	109013004	laccase-2-like
XR_001995690.2	XM_018961814.2	sense	intergenic	749	same_strand	downstream	-0.257206933	0.17002191	chr13	23858898	23860501	-	108988529	geranyl diphosphate phosphohydrolase
MSTRG.165614.1	XM_018962443.1	antisense	intergenic	2961	divergent	upstream	0.777414789	4.33E-07	chr8	10753119	10754455	-	108989001	ethylene-responsive transcription factor ERF113-like
MSTRG.80437.1	XM_018967797.2	antisense	intergenic	4295	divergent	upstream	-0.069919568	0.71351287	chr15	17341188	17343381	+	108993035	UPF0481 protein At3g47200-like
MSTRG.80437.1	XM_018967798.2	antisense	intergenic	4295	divergent	upstream	0.004310526	0.98196411	chr15	17341188	17343381	+	108993035	UPF0481 protein At3g47200-like
XR_001996514.2	XM_035695552.1	sense	intergenic	3004	same_strand	downstream	0.482667578	0.00690366	chr11	7446527	7451689	-	108993549	disease resistance protein RPV1-like
XR_004802397.1	XM_018966537.2	sense	intergenic	3213	same_strand	upstream	0.603616995	0.00041328	chr1	4288085	4293510	+	108992089	probable inactive leucine-rich repeat receptor-like protein kinase At3g03770
MSTRG.48337.3	XM_018984936.2	antisense	intergenic	1236	convergent	downstream	-0.002315049	0.99031293	chr12	22165066	22166482	-	109005863	probable membrane-associated kinase regulator 1
MSTRG.90734.1	XM_019004350.2	antisense	intergenic	318	convergent	downstream	0.129340515	0.49574811	chr16	27722533	27724030	+	109021669	uncharacterized LOC109021669
MSTRG.90734.1	XM_035686487.1	sense	intergenic	7596	same_strand	upstream	-0.06563769	0.73038899	chr16	27706855	27716752	-	109021666	ADP-ribosylation factor-like protein 8a
MSTRG.90734.1	XM_035686489.1	sense	intergenic	7595	same_strand	upstream	0.393497931	0.03144929	chr16	27706855	27716753	-	109021666	ADP-ribosylation factor-like protein 8a
MSTRG.41494.1	XM_018973911.2	sense	intergenic	3240	same_strand	downstream	-0.043344505	0.82009214	chr12	2461615	2467119	+	108997572	sulfite exporter TauE/SafE family protein 3-like
MSTRG.118412.2	XM_018974327.2	sense	intergenic	14829	same_strand	downstream	0.446040406	0.01349142	chr4	7209152	7218675	+	108997941	OVARIAN TUMOR DOMAIN-containing deubiquitinating enzyme 12-like
XR_001996792.1	XM_018970420.2	antisense	intergenic	1969	convergent	downstream	-0.03412252	0.85793215	chr7	35725527	35728689	-	108994975	uncharacterized LOC108994975
XR_001996792.1	XM_018970577.2	antisense	intergenic	6750	convergent	downstream	-0.162340186	0.39139088	chr7	35730308	35733222	-	108995074	guanylate kinase 2, chloroplastic/mitochondrial
XR_001999403.2	XM_018993477.2	antisense	intergenic	7911	divergent	upstream	0.047977284	0.80122566	chr4	2764133	2765756	+	109012042	putative DEAD-box ATP-dependent RNA helicase 33

MSTRG.47082.1	XM_035683393.1	antisense	intergenic	1686	convergent	downstream	-0.106947965	0.57377205	chr12	18835954	18853779	+	109013039	calmodulin-binding transcription activator 3-like
MSTRG.80048.6	XM_018977293.2	antisense	intergenic	73260	convergent	downstream	-0.264602956	0.15764433	chr15	15788390	15790762	+	109000426	oxygen-evolving enhancer protein 2, chloroplastic
XR_002001046.2	XM_018962409.2	sense	intergenic	3124	same_strand	upstream	0.819167899	3.12E-08	chr5	5177366	5182628	+	108988974	protein PEP-RELATED DEVELOPMENT ARRESTED 1, chloroplastic
XR_001998713.2	XM_019005128.2	sense	intergenic	1696	same_strand	downstream	0.374159492	0.04165637	chr3	8783238	8786041	-	109022266	ankyrin repeat domain-containing protein 13C-like
MSTRG.144536.8	XM_035690944.1	antisense	intergenic	7684	convergent	downstream	-0.130521321	0.49178812	chr6	38057985	38060650	-	109005979	transmembrane 9 superfamily member 11
MSTRG.148119.1	XM_035691405.1	antisense	genic	0	nested	intronic	0.090258117	0.63526609	chr7	9525623	9526915	+	108998374	uncharacterized LOC108998374
MSTRG.100779.1	XM_018979688.2	antisense	intergenic	9886	convergent	downstream	-0.026534819	0.8893033	chr2	29028145	29037315	-	109002086	cellulose synthase-like protein G2
MSTRG.100779.1	XM_018979674.2	antisense	intergenic	7940	divergent	upstream	-0.12487056	0.51088309	chr2	29005772	29009914	-	109002081	protein ABIL2-like
MSTRG.100779.1	XM_018979673.2	antisense	intergenic	7676	divergent	upstream	0.363449266	0.04835806	chr2	29005772	29010178	-	109002081	protein ABIL2-like
XR_001996962.2	XM_018972062.2	sense	intergenic	9457	same_strand	upstream	0.322346992	0.08234131	chr1	12091391	12094464	-	108996275	actin-7
MSTRG.18848.1	XM_018951314.1	antisense	intergenic	5004	divergent	upstream	0.344737487	0.0620939	chr10	9243142	9243720	-	108980404	protein LATERAL ROOT PRIMORDIUM 1-like
MSTRG.18848.1	XM_035694426.1	sense	intergenic	5034	same_strand	upstream	-0.32492383	0.07978228	chr10	9257117	9259371	+	109010172	short-chain dehydrogenase RED1-like
XR_001997771.2	XM_018977828.2	antisense	intergenic	1526	divergent	upstream	0.299735999	0.1075705	chr7	44597853	44601898	-	109000814	UDP-glycosyltransferase 74F2-like
XR_001996164.2	XM_018965867.2	antisense	intergenic	5748	convergent	downstream	0.075780677	0.69062714	chr9	16108621	16110679	+	108991560	glycine-rich protein A3
XR_001996977.2	XM_018972139.2	antisense	intergenic	13653	divergent	upstream	-0.130768131	0.49096245	chr1	12145924	12154348	+	108996313	autophagy-related protein 13b
XR_001996977.2	XM_018972141.2	antisense	intergenic	13681	divergent	upstream	-0.258275039	0.16819312	chr1	12145952	12154348	+	108996313	autophagy-related protein 13b
XR_001995212.2	XM_018957894.2	antisense	intergenic	6690	convergent	downstream	-0.287975122	0.12278661	chr8	26437882	26442263	+	108985551	heat shock 70 kDa protein, mitochondrial-like
XR_002000562.2	XM_035682813.1	antisense	intergenic	4142	convergent	downstream	0.228351015	0.22486242	chr11	31328915	31340519	+	108983177	calcium-transporting ATPase 1-like
XR_001999024.2	XM_018989724.2	sense	intergenic	1162	same_strand	downstream	-0.099512556	0.60083905	chr11	1097694	1125750	+	109009288	protein HASTY 1
MSTRG.34464.1	XM_019000722.1	antisense	intergenic	4957	divergent	upstream	0.003949441	0.98347473	chr11	21264044	21274105	-	109018557	protein NETWORKED 1D-like
MSTRG.118627.1	XM_018976846.2	antisense	intergenic	36192	divergent	upstream	-0.037770886	0.84292029	chr4	8318178	8328917	-	108999982	FAD synthase
MSTRG.111024.2	XM_035688999.1	sense	intergenic	10355	same_strand	upstream	-0.065483162	0.7310004	chr3	23438295	23440351	+	109000854	cation/calcium exchanger 2
XR_004801701.1	XM_035690662.1	antisense	intergenic	41689	convergent	downstream	-0.265478259	0.15622337	chr6	11693661	11694374	+	118348613	uncharacterized LOC118348613
XR_004802373.1	XM_018982702.2	antisense	intergenic	3236	convergent	downstream	0.522446874	0.00305957	chr1	3787244	3790889	+	109004225	putative glucose-6-phosphate 1-epimerase
MSTRG.72701.1	XM_018958290.2	antisense	intergenic	15952	divergent	upstream	0.158930711	0.40154709	chr14	21881548	21894960	+	108985848	protein SUPPRESSOR OF K(+) TRANSPORT GROWTH DEFECT 1
MSTRG.72701.1	XM_018958292.2	antisense	intergenic	15952	divergent	upstream	0.173967415	0.3578821	chr14	21881548	21894535	+	108985848	protein SUPPRESSOR OF K(+) TRANSPORT GROWTH DEFECT 1
MSTRG.72701.1	XM_018958288.2	antisense	intergenic	15952	divergent	upstream	0.089351909	0.63867921	chr14	21881548	21894227	+	108985848	protein SUPPRESSOR OF K(+) TRANSPORT GROWTH DEFECT 1
XR_001996162.2	XM_018965867.2	antisense	intergenic	2159	convergent	downstream	0.155109486	0.41310549	chr9	16108621	16110679	+	108991560	glycine-rich protein A3
XR_001994164.2	XM_018949737.2	sense	intergenic	1812	same_strand	upstream	0.021904955	0.90852925	chr3	5134345	5136464	-	108979139	polyphenol oxidase, chloroplastic-like
MSTRG.90734.2	XM_019004350.2	antisense	intergenic	318	convergent	downstream	-0.094179126	0.62058307	chr16	27722533	27724030	+	109021669	uncharacterized LOC109021669
MSTRG.90734.2	XM_035686489.1	sense	intergenic	7595	same_strand	upstream	0.318069208	0.0867278	chr16	27706855	27716753	-	109021666	ADP-ribosylation factor-like protein 8a
MSTRG.90734.2	XM_035686487.1	sense	intergenic	7596	same_strand	upstream	0.144506645	0.44613412	chr16	27706855	27716752	-	109021666	ADP-ribosylation factor-like protein 8a
XR_004797800.1	XM_018972169.2	sense	intergenic	568	same_strand	downstream	-0.417839176	0.02158472	chr1	10721028	10755178	-	108996330	twinkle homolog protein, chloroplastic/mitochondrial
XR_004797800.1	XM_018972168.2	sense	intergenic	568	same_strand	downstream	-0.550522073	0.00162018	chr1	10721028	10755178	-	108996330	twinkle homolog protein, chloroplastic/mitochondrial
XR_001995902.1	XM_018963677.2	antisense	intergenic	11222	divergent	upstream	0.758186024	1.21E-06	chr2	18819380	18820707	-	108989913	uncharacterized LOC108989913
XR_002000537.2	XM_019000541.2	antisense	intergenic	18166	divergent	upstream	0.816642867	3.73E-08	chr11	21533061	21534482	+	109018384	uncharacterized LOC109018384
MSTRG.137084.1	XM_018990444.2	antisense	genic	0	nested	intronic	-0.428241363	0.01822932	chr6	11941541	11947610	-	109009821	uncharacterized LOC109009821
MSTRG.159471.1	XM_018995105.2	sense	intergenic	2685	same_strand	upstream	0.321847687	0.08284436	chr7	46296969	46301965	+	109013124	phospholipase D alpha 1-like
MSTRG.151770.1	XM_018955886.2	antisense	intergenic	1329	convergent	downstream	-0.244208895	0.19341369	chr7	19823270	19824774	-	108984057	F-box protein At2g02240-like
MSTRG.26671.1	XM_019004093.2	antisense	intergenic	1039	convergent	downstream	-0.034925458	0.85462397	chr10	34638929	34640589	-	109021469	uncharacterized LOC109021469
XR_001998388.2	XM_018983791.2	antisense	intergenic	2588	convergent	downstream	0.05227856	0.78380652	chr12	25132131	25137820	-	109005028	ankyrin repeat protein SKIP35
XR_001998388.2	XM_018983794.2	sense	intergenic	6451	same_strand	downstream	0.623348144	0.00023344	chr12	25117144	25121104	+	109005031	uncharacterized LOC109005031
MSTRG.2509.2	XM_018961307.2	sense	intergenic	2467	same_strand	upstream	0.434265107	0.01649207	chr1	7334348	7337598	-	108988161	receptor-like protein kinase FERONIA
XR_001994840.2	XM_018954888.2	sense	intergenic	2504	same_strand	downstream	0.047152186	0.80457809	chr3	7832092	7835760	-	109011543	putative receptor-like protein kinase At4g00960
XR_001996664.2	XM_035695675.1	antisense	intergenic	1702	divergent	upstream	-0.043345044	0.82008994	chr1	12994622	12997118	+	118343699	monooxygenase 2-like
XR_001994277.2	XM_035692135.1	sense	intergenic	7014	same_strand	upstream	0.337587196	0.06808355	chr7	2355795	2369500	-	108979794	peptidyl-prolyl cis-trans isomerase CYP40-like
MSTRG.51561.8	XM_018983435.2	sense	intergenic	2240	same_strand	downstream	-0.271580797	0.14657097	chr12	31233516	31237814	-	109004760	ribosome biogenesis protein NOP53
MSTRG.51561.8	XM_018983433.2	sense	intergenic	3369	same_strand	upstream	-0.404139399	0.0267629	chr12	31222604	31226013	-	109004759	uncharacterized LOC109004759
MSTRG.25940.1	XM_018983286.2	sense	intergenic	5307	same_strand	upstream	-0.138779883	0.46454694	chr10	32250824	32262128	+	109004654	aldehyde dehydrogenase family 2 member C4-like
MSTRG.16888.1	XM_018959927.2	antisense	intergenic	5311	divergent	upstream	-0.099818974	0.5997129	chr10	3185058	3186475	+	108987076	probable transmembrane ascorbate ferrireductase 2
MSTRG.140831.1	XM_035691122.1	sense	intergenic	2455	same_strand	downstream	0.083853422	0.6595419	chr6	25877167	25897936	-	108997805	serine protease SPPA, chloroplastic-like
XR_004797794.1	XM_018973921.2	sense	intergenic	18702	same_strand	upstream	-0.165256283	0.38282254	chr12	2589879	2592141	+	108997577	probable inactive serine/threonine-protein kinase fnkC
XR_004797794.1	XM_018973924.2	sense	intergenic	18702	same_strand	upstream	-0.340475071	0.06561261	chr12	2589879	2592141	+	108997577	probable inactive serine/threonine-protein kinase fnkC
MSTRG.112953.1	XM_035688217.1	antisense	intergenic	9057	convergent	downstream	0.407224641	0.0255163	chr3	27759990	27764140	-	118347920	putative E3 ubiquitin-protein ligase RING1a
MSTRG.112953.1	XM_019003309.2	sense	intergenic	7045	same_strand	upstream	-0.167302392	0.3768759	chr3	27757978	27759285	+	109020786	auxin-repressed 12.5 kDa protein-like
MSTRG.112953.1	XM_035688211.1	sense	intergenic	3408	same_strand	downstream	-0.128661363	0.498033	chr3	27734432	27738995	+	109018462	phosphatidylinositol N-acetylglucosaminyltransferase subunit C-like

XR_001998022.2	XM_018960797.2	sense	intergenic	12236	same_strand	downstream	-0.126002089	0.50703036	chr1	13963820	13965719	+	108987789	cytochrome P450 71AU50-like
XR_004802018.1	XM_035692374.1	antisense	intergenic	3942	divergent	upstream	0.094666622	0.61876734	chr7	6139801	6147783	-	108980876	receptor like protein 21-like
XR_001999772.2	XM_019002831.2	sense	intergenic	524	same_strand	downstream	0.278005399	0.13688307	chr3	8219223	8220970	+	109020382	polyubiquitin
MSTRG.124386.2	XM_018985732.2	antisense	genic	0	nested	intronic	-0.072815183	0.70217451	chr4	28530434	28548683	-	109006450	protein NARROW LEAF 1-like
MSTRG.118412.3	XM_018974327.2	sense	intergenic	14850	same_strand	downstream	0.061457206	0.74698546	chr4	7209152	7218675	+	108997941	OVARIAN TUMOR DOMAIN-containing deubiquitinating enzyme 12-like
XR_004801702.1	XM_035690662.1	antisense	intergenic	41689	convergent	downstream	-0.275561034	0.14051239	chr6	11693661	11694374	+	118348613	uncharacterized LOC118348613
XR_004798595.1	XM_018994144.2	sense	genic	0	nested	intronic	-0.514779674	0.00360635	chr16	2963227	2972655	+	109012478	ADP-ribosylation factor 1-like 2
MSTRG.171001.1	XM_018981118.2	antisense	intergenic	22445	convergent	downstream	-0.027292236	0.88616362	chr9	613706	616104	+	109003114	magnesium protoporphyrin IX methyltransferase, chloroplastic
MSTRG.171001.1	XM_018981109.2	antisense	intergenic	23035	convergent	downstream	-0.398100265	0.02934688	chr9	613705	615514	+	109003114	magnesium protoporphyrin IX methyltransferase, chloroplastic
XR_004801712.1	XM_018990444.2	sense	intergenic	33426	same_strand	downstream	0.164345358	0.38548735	chr6	11941541	11947610	-	109009821	uncharacterized LOC109009821
MSTRG.100220.2	XM_019003581.2	antisense	intergenic	511	divergent	upstream	-0.225930598	0.22994947	chr2	28085712	28088527	-	109021046	alcohol dehydrogenase-like 4
MSTRG.100220.2	XM_019003583.2	sense	intergenic	3716	same_strand	downstream	0.341708026	0.06457919	chr2	28078440	28085322	+	109021047	polyadenylate-binding protein-interacting protein 11
XR_001996057.2	XM_018964910.2	sense	intergenic	18744	same_strand	downstream	-0.149671107	0.42987218	chr10	27274218	27276653	-	108990815	L10-interacting MYB domain-containing protein-like
XR_004801170.1	XM_018978584.2	antisense	intergenic	5180	divergent	upstream	0.320110745	0.08461267	chr1	1144785	1149229	-	109001337	protein trichome birefringence-like 19
XR_004798313.1	XM_018969039.2	sense	intergenic	9751	same_strand	downstream	0.220576512	0.2414754	chr14	10393260	10401750	+	108993968	kinesin-like protein KIN-5D
MSTRG.100871.1	XM_018959997.1	antisense	intergenic	7358	convergent	downstream	-0.172716696	0.36140223	chr2	29480152	29480490	-	108987135	uncharacterized LOC108987135
XR_004802796.1	XM_018966504.2	antisense	intergenic	2605	convergent	downstream	0.209011989	0.26766333	chr11	8752363	8754969	+	108992054	cytochrome P450 84A1-like
XR_004802796.1	XM_018966443.2	sense	intergenic	4180	same_strand	downstream	0.440358891	0.01487669	chr11	8770598	8777661	-	108992009	protein FAR1-RELATED SEQUENCE 4-like
MSTRG.37463.1	XM_018953402.2	antisense	intergenic	9639	divergent	upstream	0.2036204	0.28047993	chr11	28430134	28436281	+	108982114	synaptotagmin-2
MSTRG.37463.1	XM_035682663.1	antisense	intergenic	9639	divergent	upstream	0.036761138	0.84706992	chr11	28430134	28436281	+	108982114	synaptotagmin-2
MSTRG.98044.1	XM_018954919.2	antisense	genic	0	nested	intronic	0.77909017	3.94E-07	chr2	19452380	19474857	+	108983325	beta-amylase 8
MSTRG.104630.1	XM_018993347.2	sense	intergenic	9641	same_strand	upstream	-0.089685154	0.63742324	chr3	2542281	2544472	-	109011940	thioredoxin-like 3-1, chloroplastic
MSTRG.104630.1	XM_018993346.2	sense	intergenic	9641	same_strand	upstream	0.538249322	0.00215363	chr3	2542281	2544472	-	109011940	thioredoxin-like 3-1, chloroplastic
MSTRG.122935.1	XM_018964197.2	antisense	intergenic	3914	convergent	downstream	-0.116499974	0.5398259	chr4	24358773	24365331	+	108990278	putative ALA-interacting subunit 2
XR_001995764.2	XM_018962422.2	sense	intergenic	772	same_strand	downstream	0.05261276	0.78245725	chr7	33413336	33423071	+	108988986	uncharacterized LOC108988986
MSTRG.129890.1	XM_018989772.2	sense	intergenic	2608	same_strand	downstream	0.112236447	0.55485975	chr5	9979644	9981954	+	109009326	LOB domain-containing protein 1-like
MSTRG.109897.16	XM_035695686.1	sense	intergenic	8256	same_strand	upstream	0.643278555	0.0019517	chr11	36778145	36781067	-	108987657	serine/threonine-protein kinase-like protein CCR4
MSTRG.109897.16	XM_035682624.1	sense	intergenic	9362	same_strand	downstream	-0.053394677	0.77930283	chr11	36753010	36757550	+	109005180	stromal 70 kDa heat shock-related protein, chloroplastic-like
MSTRG.109897.16	XM_018960630.2	antisense	intergenic	9534	convergent	downstream	0.430838002	0.01746275	chr11	36781824	36786788	-	108987664	receptor protein-tyrosine kinase CEPR2-like
MSTRG.109897.16	XM_018983982.2	antisense	intergenic	633	divergent	upstream	0.348022628	0.05948403	chr11	36760138	36766279	-	109005179	hydroxypyruvate reductase
MSTRG.161579.1	XM_018966159.2	sense	intergenic	1313	same_strand	upstream	-0.016103791	0.93268954	chr7	51832611	51835986	-	108991773	BTB/POZ domain-containing protein At3g56230
MSTRG.67831.1	XM_018994906.2	sense	intergenic	40088	same_strand	upstream	0.035701707	0.85142803	chr14	8756286	8761612	+	109013006	abscisic acid 8'-hydroxylase 2
XR_004801644.1	XM_018960548.2	sense	intergenic	1290	same_strand	upstream	-0.261032414	0.16353662	chr6	9639646	9640863	+	108987614	uncharacterized LOC108987614
XR_004801285.1	XM_018960797.2	sense	intergenic	12236	same_strand	downstream	-0.053898458	0.77727229	chr1	13963820	13965719	+	108987789	cytochrome P450 71AU50-like
XR_001997032.2	XM_018972562.2	antisense	intergenic	228	convergent	downstream	0.052665168	0.78224572	chr3	10415347	10418675	-	108996581	glucan endo-1,3-beta-glucosidase 5-like
XR_004802646.1	XM_035695038.1	antisense	intergenic	31838	convergent	downstream	-0.051337037	0.78761103	chr10	21123390	21127945	+	108999736	glycosyl hydrolase 5 family protein-like
XR_004801700.1	XM_035690662.1	antisense	intergenic	41689	convergent	downstream	-0.308480831	0.09720548	chr6	11693661	11694374	+	118348613	uncharacterized LOC118348613
MSTRG.132425.2	XM_018955560.2	sense	intergenic	628	same_strand	upstream	0.589193122	0.00061314	chr5	19081757	19085202	-	108983798	tubulin beta-4 chain
MSTRG.95784.1	XM_035687678.1	antisense	intergenic	9	convergent	downstream	0.73753512	3.32E-06	chr2	14350547	14351290	-	118347663	uncharacterized protein At4g02000-like
XR_004801872.1	XM_018970789.2	sense	intergenic	6566	same_strand	upstream	0.58496083	0.000686	chr7	3226540	3227340	+	108995258	STS14 protein
XR_004801872.1	XM_018970790.2	antisense	intergenic	8284	convergent	downstream	0.646429254	0.00011373	chr7	3228258	3234529	-	108995260	uncharacterized LOC108995260
XR_004802647.1	XM_035695038.1	antisense	intergenic	31838	convergent	downstream	-0.018241543	0.92377843	chr10	21123390	21127945	+	108999736	glycosyl hydrolase 5 family protein-like
XR_001999566.2	XM_018995272.2	antisense	intergenic	7350	divergent	upstream	-0.181401209	0.33738351	chr1	14606943	14610702	-	109013245	probable glucan 1,3-beta-glucosidase A
MSTRG.14168.1	XM_018971175.2	antisense	intergenic	8266	convergent	downstream	0.444339968	0.01389424	chr1	41383707	41386728	-	108995585	heptahelical transmembrane protein 1-like

Table S3. GO and KEGG analysis of the cis-target mRNAs

GO_accession	Description	GeneNames	Ontology	pValue	qValue	fg	bg
GO:0010207	photosystem II assembly	XM_019001875.2	BP	0.006	0.048	1	5
GO:0042447	hormone catabolic process	XM_035685058.1	BP	0.0072	0.0288	1	6
GO:0006814	sodium ion transport	XM_035695610.1	BP	0.0131	0.034933333	1	11
GO:0019684	photosynthesis, light reaction	XM_019001875.2	BP	0.0213	0.0426	1	18
GO:0009850	auxin metabolic process	XM_035685058.1	BP	0.0272	0.04352	1	23
GO:0009658	chloroplast organization	XM_018962409.2	BP	0.0319	0.042533333	1	27
GO:0009657	plastid organization	XM_018962409.2	BP	0.0353	0.040342857	1	30
GO:0006413	translational initiation	XM_019001875.2	BP	0.0434	0.0434	1	37
GO:0043022	ribosome binding	XM_019001875.2	MF	0.0061	0.0244	1	5
GO:0102229	amylopectin maltohydrolase activity	XM_018954919.2	MF	0.0074	0.0148	1	6
GO:0043021	ribonucleoprotein complex binding	XM_019001875.2	MF	0.0135	0.018	1	11
GO:0044877	protein-containing complex binding	XM_019001875.2	MF	0.0244	0.0244	1	20
GO:0042644	chloroplast nucleoid	XM_018962409.2	CC	0.016	0.053333333	1	15
GO:0042646	plastid nucleoid	XM_018962409.2	CC	0.016	0.053333333	1	15
GO:0009295	nucleoid	XM_018962409.2	CC	0.019	0.031666667	1	17
GO:0009570	chloroplast stroma	XM_018962409.2,X[illegible]	CC	0.021	0.02625	2	212
GO:0009532	plastid stroma	XM_018962409.2,X[illegible]	CC	0.022	0.022	2	214
PathwayID	PathwayName	GenesName	S gene number	B gene number	Pvalue	Qvalue	PathwayLink
ko00603	Glycosphingolipid biosynthesis - globo and isoglobo series	XM_018970292.2	1	14	0.012162413	0.105359863	http://www.genome.jp/kegg-bin/show_pathway?ko00603/K01988%09red
ko04540	Gap junction	XM_018955560.2	1	24	0.020190385	0.105359863	http://www.genome.jp/kegg-bin/show_pathway?ko04540/K07375%09red
ko05130	Pathogenic Escherichia coli infection	XM_018955560.2	1	36	0.02973996	0.105359863	http://www.genome.jp/kegg-bin/show_pathway?ko05130/K07375%09red
ko00909	Sesquiterpenoid and triterpenoid biosynthesis	XM_018967924.2	1	44	0.036055832	0.105359863	http://www.genome.jp/kegg-bin/show_pathway?ko00909/K20659%09red
ko00460	Cyanoamino acid metabolism	XM_018964275.2	1	46	0.037628522	0.105359863	http://www.genome.jp/kegg-bin/show_pathway?ko00460/K13051%09red
ko00250	Alanine, aspartate and glutamate metabolism	XM_018964275.2	1	59	0.047790162	0.111510378	http://www.genome.jp/kegg-bin/show_pathway?ko00250/K13051%09red
ko04145	Phagosome	XM_018955560.2	1	112	0.088145879	0.156569358	http://www.genome.jp/kegg-bin/show_pathway?ko04145/K07375%09red
ko01110	Biosynthesis of secondary metabolites	XM_018982702.2|X[illegible]	3	1299	0.089468205	0.156569358	http://www.genome.jp/kegg-bin/show_pathway?ko01110/K20659%09red/K01792%09red/K13051%09red
ko00010	Glycolysis / Gluconeogenesis	XM_018982702.2	1	142	0.110244025	0.169534812	http://www.genome.jp/kegg-bin/show_pathway?ko00010/K01792%09red
ko00500	Starch and sucrose metabolism	XM_018954919.2	1	157	0.121096294	0.169534812	http://www.genome.jp/kegg-bin/show_pathway?ko00500/K01177%09red
ko01100	Metabolic pathways	XM_018982702.2|X[illegible]	4	2532	0.150628284	0.191708725	http://www.genome.jp/kegg-bin/show_pathway?ko01100/K01988%09red/K01792%09red/K13051%09red/K01177%09red
ko04141	Protein processing in endoplasmic reticulum	XM_019002058.2	1	250	0.185554528	0.216480283	http://www.genome.jp/kegg-bin/show_pathway?ko04141/K10666%09red
ko01120	Microbial metabolism in diverse environments	XM_018982702.2	1	415	0.288751602	0.310963264	http://www.genome.jp/kegg-bin/show_pathway?ko01120/K01792%09red
ko01130	Biosynthesis of antibiotics	XM_018982702.2	1	544	0.360437862	0.360437862	http://www.genome.jp/kegg-bin/show_pathway?ko01130/K01792%09red

Table S4. The lncRNA-mRNA coexpression network analysis in the MEdarkturquoise module									
LncRNA	mRNA	weight	mRNA information						
			chromosome	start	end	strand	gene ID	gene description
XR_004800885.1	XM_018967924.2	0.180525806	chr15	18567676	18577735	-	108993143	lupeol synthase-like
MSTRG.142498.1	XM_019003778.2	0.172636079	chr2	30319348	30320312	-	109021187	late embryogenesis abundant protein At1g64065-like
XR_004800885.1	XM_035692760.1	0.169908222	chr1	18658866	18661714	+	109010137	uncharacterized LOC109010137
XR_004800885.1	XM_018989557.2	0.167927289	chr7	8806772	8807730	+	109009171	uncharacterized LOC109009171
XR_004800885.1	XM_018966836.2	0.16750495	chr15	6744949	6749594	+	108992307	glutamate receptor 2.2-like
MSTRG.142498.1	XM_018952208.2	0.164029899	chr4	20732565	20739290	-	108981125	probable LRR receptor-like serine/threonine-protein kinase At3g47570
XR_004801042.1	XM_018951732.2	0.16037326	chr8	19850173	19851939	+	108980731	uncharacterized LOC108980731
XR_004800885.1	XM_018961525.2	0.160065448	chr13	35130904	35135396	+	108988296	cyclic nucleotide-gated ion channel 1-like
MSTRG.109897.16	XM_035695686.1	0.156184094	chr11	36778145	36781067	-	108987657	serine/threonine-protein kinase-like protein CCR4
XR_004800885.1	XM_018974702.2	0.154710526	chr6	27221507	27232906	+	108998204	thaumatin-like protein 1b
XR_004801535.1	XM_035683883.1	0.154446409	chr1	33416777	33424440	-	118344063	uncharacterized LOC118344063
XR_004800885.1	XM_019003778.2	0.152825343	chr2	30319348	30320312	-	109021187	late embryogenesis abundant protein At1g64065-like
XR_004798497.1	XM_018991068.2	0.150440637	chr6	10519971	10524297	+	109010290	cytochrome P450 CYP82D47-like
XR_001994995.2	XM_035690805.1	0.150091126	chr6	36329659	36333134	+	118343755	disease resistance protein RUN1-like
MSTRG.2510.1	XM_018951732.2	0.149566388	chr8	19850173	19851939	+	108980731	uncharacterized LOC108980731
MSTRG.122249.3	XM_035683883.1	0.149053691	chr1	33416777	33424440	-	118344063	uncharacterized LOC118344063
MSTRG.109897.16	XM_035690204.1	0.148891617	chr5	19301466	19309978	+	109004040	uncharacterized LOC109004040
XR_001999201.2	XM_018951732.2	0.148528286	chr8	19850173	19851939	+	108980731	uncharacterized LOC108980731
XR_004802642.1	XM_035683649.1	0.14847492	chr12	3976006	3985355	+	108983218	cell division protein FtsY homolog, chloroplastic
XR_001994779.2	XM_018965639.2	0.147414862	chr11	29342015	29345253	-	108991400	probable ubiquitin-conjugating enzyme E2 16
XR_004801042.1	XM_035683649.1	0.147099534	chr12	3976006	3985355	+	108983218	cell division protein FtsY homolog, chloroplastic
XR_001994779.2	XM_018970957.2	0.1467142	chr7	4064116	4068193	+	108995385	phosphatidylinositol transfer protein 3
XR_004802642.1	XM_018994818.2	0.146312815	chr6	37006877	37008290	+	109012917	class V chitinase-like
MSTRG.102213.1	XM_035690805.1	0.145591152	chr6	36329659	36333134	+	118343755	disease resistance protein RUN1-like
MSTRG.102213.1	XM_035685276.1	0.145064173	chr14	28497998	28503114	+	109003650	YTH domain-containing protein ECT4
MSTRG.55875.1	XM_035683883.1	0.145063933	chr1	33416777	33424440	-	118344063	uncharacterized LOC118344063
XR_004802642.1	XM_035690204.1	0.14460802	chr5	19301466	19309978	+	109004040	uncharacterized LOC109004040
MSTRG.142498.1	XM_018991742.2	0.144001143	chr13	34050402	34054186	+	109010823	putative wall-associated receptor kinase-like 16
XR_001994779.2	XM_018989717.2	0.143783953	chr6	17738434	17750081	+	109009277	guanine nucleotide-binding protein alpha-1 subunit
XR_004802604.1	XM_018987438.2	0.143719743	chr13	3254668	3258039	+	109007672	probable leucine-rich repeat receptor-like protein kinase At1g35710
XR_004802642.1	XM_035683883.1	0.143582677	chr1	33416777	33424440	-	118344063	uncharacterized LOC118344063
XR_001994779.2	XM_035683883.1	0.142967697	chr1	33416777	33424440	-	118344063	uncharacterized LOC118344063
XR_004802642.1	XM_018951732.2	0.142693511	chr8	19850173	19851939	+	108980731	uncharacterized LOC108980731
XR_004800885.1	XM_018967935.2	0.141985491	chr15	18383149	18385058	-	108993149	ABC transporter I family member 17-like
XR_001998020.2	XM_018951732.2	0.141337877	chr8	19850173	19851939	+	108980731	uncharacterized LOC108980731
XR_004801042.1	XM_018958370.2	0.140189409	chr12	6587489	6596583	+	108985905	uncharacterized LOC108985905
MSTRG.109897.16	XM_018968936.2	0.139835169	chr10	17539263	17544420	+	108993885	CBS domain-containing protein CBSX1, chloroplastic-like
MSTRG.116423.3	XM_018991068.2	0.139461105	chr6	10519971	10524297	+	109010290	cytochrome P450 CYP82D47-like
XR_001994779.2	XM_018982562.2	0.139265895	chr10	9156607	9159414	-	109004129	cytochrome P450 71AU50-like
MSTRG.144914.1	XM_035683883.1	0.139133955	chr1	33416777	33424440	-	118344063	uncharacterized LOC118344063
XR_001999980.2	XM_035683649.1	0.138916486	chr12	3976006	3985355	+	108983218	cell division protein FtsY homolog, chloroplastic
XR_004798497.1	XM_035690204.1	0.138837185	chr5	19301466	19309978	+	109004040	uncharacterized LOC109004040
MSTRG.109897.16	XM_018994967.2	0.138644309	chr12	18754807	18765428	-	109013042	pleiotropic drug resistance protein 3-like
XR_004801042.1	XM_018958924.2	0.138203259	chr7	50434181	50439306	+	108986346	serine/arginine-rich SC35-like splicing factor SCL30
XR_004800885.1	XM_018991068.2	0.137991016	chr6	10519971	10524297	+	109010290	cytochrome P450 CYP82D47-like
XR_001999980.2	XM_035690204.1	0.137988152	chr5	19301466	19309978	+	109004040	uncharacterized LOC109004040
XR_001999980.2	XM_035683883.1	0.137834047	chr1	33416777	33424440	-	118344063	uncharacterized LOC118344063
MSTRG.142498.1	XM_018989557.2	0.137744363	chr7	8806772	8807730	+	109009171	uncharacterized LOC109009171

MSTRG.2510.1	XM_035683649.1	0.137692545	chr12	3976006	3985355	+	108983218	cell division protein FtsY homolog, chloroplastic
XR_004802196.1	XM_035685276.1	0.137589026	chr14	28497998	28503114	+	109003650	YTH domain-containing protein ECT4
MSTRG.142498.1	XM_018972248.2	0.137535421	chr1	12058395	12061891	+	108996378	receptor-like protein EIX2
XR_004801671.1	XM_035683649.1	0.137316149	chr12	3976006	3985355	+	108983218	cell division protein FtsY homolog, chloroplastic
XR_001995964.2	XM_018962761.2	0.136771048	chr14	8536278	8538423	-	108989223	probable L-type lectin-domain containing receptor kinase I.6
XR_004802604.1	XM_018956479.2	0.136679145	chr13	38279223	38281668	-	108984497	rust resistance kinase Lr10-like
MSTRG.152528.3	XM_018962761.2	0.13664359	chr14	8536278	8538423	-	108989223	probable L-type lectin-domain containing receptor kinase I.6
XR_001998573.2	XM_035683883.1	0.13659688	chr1	33416777	33424440	-	118344063	uncharacterized LOC118344063
XR_001998020.2	XM_018994818.2	0.136443265	chr6	37006877	37008290	+	109012917	class V chitinase-like
MSTRG.109897.16	XM_018951732.2	0.136338206	chr8	19850173	19851939	+	108980731	uncharacterized LOC108980731
MSTRG.109897.16	XM_035683649.1	0.135865597	chr12	3976006	3985355	+	108983218	cell division protein FtsY homolog, chloroplastic
XR_001999980.2	XM_018987330.2	0.135850369	chr16	7075619	7089046	+	109007582	uncharacterized LOC109007582
XR_004798497.1	XM_018994818.2	0.1356572	chr6	37006877	37008290	+	109012917	class V chitinase-like
XR_001996304.1	XM_035683883.1	0.135436404	chr1	33416777	33424440	-	118344063	uncharacterized LOC118344063
MSTRG.144914.1	XM_035695686.1	0.13529965	chr11	36778145	36781067	-	108987657	serine/threonine-protein kinase-like protein CCR4
XR_001995963.2	XM_035683883.1	0.13527562	chr1	33416777	33424440	-	118344063	uncharacterized LOC118344063
MSTRG.122249.3	XM_018959084.2	0.135265513	chr10	4905053	4907686	-	108986462	transcription factor TCP13-like
XR_001996083.2	XM_035685276.1	0.134886474	chr14	28497998	28503114	+	109003650	YTH domain-containing protein ECT4
XR_004801970.1	XM_035690668.1	0.134715568	chr6	12923505	12926199	-	108995545	receptor-like protein 7
MSTRG.109897.16	XM_018988367.2	0.134543592	chr2	26695615	26698934	-	109008319	disease resistance protein RPM1-like
MSTRG.153333.1	XM_018962761.2	0.134440828	chr14	8536278	8538423	-	108989223	probable L-type lectin-domain containing receptor kinase I.6
XR_001994779.2	XM_035691872.1	0.134096672	chr7	48872965	48879686	+	108997043	pumilio homolog 4-like
MSTRG.109897.16	XM_018987330.2	0.13405496	chr16	7075619	7089046	+	109007582	uncharacterized LOC109007582
MSTRG.122249.3	XM_018951732.2	0.133956034	chr8	19850173	19851939	+	108980731	uncharacterized LOC108980731
XR_001994779.2	XM_035691979.1	0.133766462	chr7	39113205	39115741	+	108991199	putative protein phosphatase 2C-like protein 44
XR_001998020.2	XM_018987325.2	0.133442675	chr16	7077657	7089046	+	109007582	uncharacterized LOC109007582
MSTRG.109897.16	XM_018997560.2	0.133337197	chr16	21638118	21646738	+	109015070	probable isoprenylcysteine alpha-carbonyl methylesterase ICMEL1
XR_004802642.1	XM_018958924.2	0.133075876	chr7	50434181	50439306	+	108986346	serine/arginine-rich SC35-like splicing factor SCL30
XR_004800885.1	XM_018963149.2	0.132827336	chr6	35275634	35278794	+	108989520	beta-amyrin 28-monooxygenase-like
XR_004800885.1	XM_018952208.2	0.132805363	chr4	20732565	20739290	-	108981125	probable LRR receptor-like serine/threonine-protein kinase At3g47570
XR_001995902.1	XM_035683883.1	0.132733992	chr1	33416777	33424440	-	118344063	uncharacterized LOC118344063
XR_004798497.1	XM_035683649.1	0.132652696	chr12	3976006	3985355	+	108983218	cell division protein FtsY homolog, chloroplastic
XR_004800885.1	XM_018979640.2	0.132489975	chr12	5228351	5232321	+	109002057	uncharacterized LOC109002057
XR_004801700.1	XM_018950215.2	0.132402068	chr12	17664769	17667678	+	108979521	receptor-like protein 7
XR_004801535.1	XM_018982562.2	0.132138452	chr10	9156607	9159414	-	109004129	cytochrome P450 71AU50-like
XR_001996304.1	XM_018965639.2	0.132118012	chr11	29342015	29345253	-	108991400	probable ubiquitin-conjugating enzyme E2 16
XR_004802604.1	XM_018962761.2	0.132030845	chr14	8536278	8538423	-	108989223	probable L-type lectin-domain containing receptor kinase I.6
XR_001999201.2	XM_018987325.2	0.13201936	chr16	7077657	7089046	+	109007582	uncharacterized LOC109007582
XR_001994779.2	XM_018971230.2	0.131766219	chr1	41589670	41591745	-	108995640	protein SRG1-like
XR_004802642.1	XM_018958370.2	0.131645041	chr12	6587489	6596583	+	108985905	uncharacterized LOC108985905
XR_001999201.2	XM_018968358.2	0.131457313	chr13	6148753	6152322	+	108993435	probable amidase At4g34880
MSTRG.142498.1	XM_018987438.2	0.131432086	chr13	3254668	3258039	+	109007672	probable leucine-rich repeat receptor-like protein kinase At1g35710
XR_004801042.1	XM_018987325.2	0.131362248	chr16	7077657	7089046	+	109007582	uncharacterized LOC109007582
XR_001996083.2	XM_035683883.1	0.131145078	chr1	33416777	33424440	-	118344063	uncharacterized LOC118344063
XR_004801535.1	XM_018959084.2	0.13089019	chr10	4905053	4907686	-	108986462	transcription factor TCP13-like
XR_004801671.1	XM_018951732.2	0.130618965	chr8	19850173	19851939	+	108980731	uncharacterized LOC108980731
MSTRG.142498.1	XM_018972624.2	0.130545438	chr4	20534888	20540216	-	108996628	probable LRR receptor-like serine/threonine-protein kinase At3g47570
XR_004798497.1	XM_035683883.1	0.13034774	chr1	33416777	33424440	-	118344063	uncharacterized LOC118344063
MSTRG.2510.1	XM_018959084.2	0.130214771	chr10	4905053	4907686	-	108986462	transcription factor TCP13-like
XR_004802642.1	XM_018991068.2	0.130188218	chr6	10519971	10524297	+	109010290	cytochrome P450 CYP82D47-like
XR_001996083.2	XM_018959084.2	0.130187055	chr10	4905053	4907686	-	108986462	transcription factor TCP13-like

XR_004801970.1	XM_018955260.1	0.129888831	chr7	5726972	5727550	+	108983577	receptor like protein 21-like
XR_001995963.2	XM_035691872.1	0.129442177	chr7	48872965	48879686	+	108997043	pumilio homolog 4-like
XR_004801535.1	XM_035691872.1	0.129287517	chr7	48872965	48879686	+	108997043	pumilio homolog 4-like
XR_001998161.2	XM_035683883.1	0.129225084	chr1	33416777	33424440	-	118344063	uncharacterized LOC118344063
XR_004801042.1	XM_035690204.1	0.129187885	chr5	19301466	19309978	+	109004040	uncharacterized LOC109004040
MSTRG.109897.16	XM_018959084.2	0.129151857	chr10	4905053	4907686	-	108986462	transcription factor TCP13-like
XR_001998020.2	XM_018958370.2	0.129038923	chr12	6587489	6596583	+	108985905	uncharacterized LOC108985905
MSTRG.142498.1	XM_018950215.2	0.128870501	chr12	17664769	17667678	+	108979521	receptor-like protein 7
XR_001996832.2	XM_018994818.2	0.128826772	chr6	37006877	37008290	+	109012917	class V chitinase-like
XR_004801671.1	XM_018987330.2	0.128777797	chr16	7075619	7089046	+	109007582	uncharacterized LOC109007582
XR_004801106.1	XM_035690204.1	0.128581783	chr5	19301466	19309978	+	109004040	uncharacterized LOC109004040
XR_004802604.1	XM_019003778.2	0.128519586	chr2	30319348	30320312	-	109021187	late embryogenesis abundant protein At1g64065-like
MSTRG.152528.3	XM_018956479.2	0.128235319	chr13	38279223	38281668	-	108984497	rust resistance kinase Lr10-like
MSTRG.112953.5	XM_035683649.1	0.128202435	chr12	3976006	3985355	+	108983218	cell division protein FtsY homolog, chloroplastic
MSTRG.122249.3	XM_035683649.1	0.12816017	chr12	3976006	3985355	+	108983218	cell division protein FtsY homolog, chloroplastic
XR_001998020.2	XM_035683883.1	0.128077259	chr1	33416777	33424440	-	118344063	uncharacterized LOC118344063
XR_001998020.2	XM_035690204.1	0.12806839	chr5	19301466	19309978	+	109004040	uncharacterized LOC109004040
XR_001994995.2	XM_035685276.1	0.128062078	chr14	28497998	28503114	+	109003650	YTH domain-containing protein ECT4
MSTRG.112953.5	XM_035695686.1	0.127645108	chr11	36778145	36781067	-	108987657	serine/threonine-protein kinase-like protein CCR4
XR_004802642.1	XM_018959084.2	0.127636259	chr10	4905053	4907686	-	108986462	transcription factor TCP13-like
XR_004801042.1	XM_018959084.2	0.127585259	chr10	4905053	4907686	-	108986462	transcription factor TCP13-like
MSTRG.109897.16	XM_018958370.2	0.127512228	chr12	6587489	6596583	+	108985905	uncharacterized LOC108985905
XR_004802642.1	XM_018987325.2	0.127490379	chr16	7077657	7089046	+	109007582	uncharacterized LOC109007582
XR_004798497.1	XM_035692041.1	0.12746178	chr7	40919411	40922968	-	118349009	1,2-dihydroxy-3-keto-5-methylthiopentene dioxygenase 2
XR_001996083.2	XM_018951732.2	0.127274243	chr8	19850173	19851939	+	108980731	uncharacterized LOC108980731
MSTRG.109897.16	XM_018958924.2	0.127217875	chr7	50434181	50439306	+	108986346	serine/arginine-rich SC35-like splicing factor SCL30
XR_004801535.1	XM_035683649.1	0.127026635	chr12	3976006	3985355	+	108983218	cell division protein FtsY homolog, chloroplastic
XR_004801535.1	XM_018970957.2	0.126865055	chr7	4064116	4068193	+	108995385	phosphatidylinositol transfer protein 3
MSTRG.102213.1	XM_018996109.2	0.126268549	chr7	1023081	1024482	+	109013875	probable esterase D14L
XR_004801905.1	XM_035690204.1	0.126243836	chr5	19301466	19309978	+	109004040	uncharacterized LOC109004040
XR_004802604.1	XM_018972248.2	0.126204286	chr1	12058395	12061891	+	108996378	receptor-like protein EIX2
XR_004802196.1	XM_018996109.2	0.12607239	chr7	1023081	1024482	+	109013875	probable esterase D14L
XR_004801159.1	XM_018987438.2	0.125989009	chr13	3254668	3258039	+	109007672	probable leucine-rich repeat receptor-like protein kinase At1g35710
XR_001998063.2	XM_018982562.2	0.125986104	chr10	9156607	9159414	-	109004129	cytochrome P450 71AU50-like
XR_004801970.1	XM_019001406.2	0.12597606	chr2	6156866	6160286	-	109019170	MDIS1-interacting receptor like kinase 2-like
XR_004801905.1	XM_018951732.2	0.125541099	chr8	19850173	19851939	+	108980731	uncharacterized LOC108980731
MSTRG.152528.3	XM_018991068.2	0.125457572	chr6	10519971	10524297	+	109010290	cytochrome P450 CYP82D47-like
MSTRG.122249.3	XM_018989717.2	0.125434658	chr6	17738434	17750081	+	109009277	guanine nucleotide-binding protein alpha-1 subunit
MSTRG.122249.3	XM_035685276.1	0.125283313	chr14	28497998	28503114	+	109003650	YTH domain-containing protein ECT4
MSTRG.109897.16	XM_018994051.2	0.125193887	chr10	3593134	3595209	-	109012426	putative glutamine amidotransferase GAT1_2.1
XR_004801700.1	XM_019003778.2	0.125177736	chr2	30319348	30320312	-	109021187	late embryogenesis abundant protein At1g64065-like
MSTRG.102213.1	XM_018984380.2	0.125071048	chr7	42851936	42854516	+	109005433	dormancy-associated protein homolog 4
MSTRG.109897.16	XM_018986529.2	0.12494133	chr13	3859017	3862496	-	109007037	transmembrane emp24 domain-containing protein p24delta3-like
MSTRG.116423.3	XM_035690204.1	0.124840591	chr5	19301466	19309978	+	109004040	uncharacterized LOC109004040
XR_001998020.2	XM_035683649.1	0.124613411	chr12	3976006	3985355	+	108983218	cell division protein FtsY homolog, chloroplastic
XR_004801042.1	XM_035683883.1	0.124542676	chr1	33416777	33424440	-	118344063	uncharacterized LOC118344063
MSTRG.2510.1	XM_035683883.1	0.124489263	chr1	33416777	33424440	-	118344063	uncharacterized LOC118344063
MSTRG.142498.1	XM_018973498.2	0.124487239	chr9	19567286	19568126	-	108997294	uncharacterized LOC108997294
MSTRG.27387.1	XM_018951732.2	0.124486225	chr8	19850173	19851939	+	108980731	uncharacterized LOC108980731
XR_001995963.2	XM_018959084.2	0.124282977	chr10	4905053	4907686	-	108986462	transcription factor TCP13-like
XR_001998063.2	XM_018970957.2	0.124278251	chr7	4064116	4068193	+	108995385	phosphatidylinositol transfer protein 3

XR_001996304.1	XM_018982562.2	0.124223506	chr10	9156607	9159414	-	109004129	cytochrome P450 71AU50-like
XR_001994779.2	XM_019002925.2	0.124098863	chr6	34464405	34467297	-	109020464	F-box protein At5g07610-like
XR_004801970.1	XM_018979100.2	0.123946432	chr14	27324233	27326650	+	109001703	receptor-like protein 2
XR_001996832.2	XM_035695686.1	0.123922075	chr11	36778145	36781067	-	108987657	serine/threonine-protein kinase-like protein CCR4
XR_001998063.2	XM_018965639.2	0.123841763	chr11	29342015	29345253	-	108991400	probable ubiquitin-conjugating enzyme E2 16
MSTRG.27387.1	XM_035683883.1	0.123833257	chr1	33416777	33424440	-	118344063	uncharacterized LOC118344063
MSTRG.122249.3	XM_035690204.1	0.123782084	chr5	19301466	19309978	+	109004040	uncharacterized LOC109004040
XR_004800885.1	XM_018972248.2	0.123780169	chr1	12058395	12061891	+	108996378	receptor-like protein EIX2
XR_004801700.1	XM_018987438.2	0.123753762	chr13	3254668	3258039	+	109007672	probable leucine-rich repeat receptor-like protein kinase At1g35710
XR_004801535.1	XM_018965639.2	0.123486128	chr11	29342015	29345253	-	108991400	probable ubiquitin-conjugating enzyme E2 16
XR_001994995.2	XM_035688887.1	0.123357942	chr3	28664621	28667043	+	118348056	MDIS1-interacting receptor like kinase 2-like
MSTRG.109897.16	XM_018991132.2	0.123228582	chr16	23641881	23650296	+	109010332	uncharacterized LOC109010332
MSTRG.109897.16	XM_018974902.2	0.123121421	chr6	22995067	22999378	-	108998362	isocitrate dehydrogenase [NADP]-like
XR_004801905.1	XM_035683649.1	0.12282283	chr12	3976006	3985355	+	108983218	cell division protein FtsY homolog, chloroplastic
XR_001994779.2	XM_018979100.2	0.122797179	chr14	27324233	27326650	+	109001703	receptor-like protein 2
XR_001999980.2	XM_018951732.2	0.1227502	chr8	19850173	19851939	+	108980731	uncharacterized LOC108980731
XR_004801905.1	XM_018987330.2	0.1227259	chr16	7075619	7089046	+	109007582	uncharacterized LOC109007582
MSTRG.97720.1	XM_035683883.1	0.122681927	chr1	33416777	33424440	-	118344063	uncharacterized LOC118344063
MSTRG.109897.16	XM_018966810.2	0.122649653	chr13	30887602	30901317	+	108992290	uncharacterized LOC108992290
XR_001996083.2	XM_018996109.2	0.122634749	chr7	1023081	1024482	+	109013875	probable esterase D14L
XR_004798497.1	XM_018991132.2	0.122634653	chr16	23641881	23650296	+	109010332	uncharacterized LOC109010332
XR_004801970.1	XM_018961666.2	0.122477504	chr5	508352	512482	+	108988401	protein RETICULATA-RELATED 4, chloroplastic-like
XR_004801970.1	XM_018952493.2	0.122452464	chr6	2762461	2764510	+	108981371	uncharacterized LOC108981371
MSTRG.102213.1	XM_035688887.1	0.122432837	chr3	28664621	28667043	+	118348056	MDIS1-interacting receptor like kinase 2-like
MSTRG.109897.16	XM_035689691.1	0.122250228	chr4	2229966	2235819	-	109000808	acetyl-coenzyme A carboxylase carboxyl transferase subunit alpha, chloroplastic-like
XR_004802642.1	XM_018991596.2	0.122177154	chr12	28817542	28820025	-	109010697	vestitone reductase-like
MSTRG.109897.16	XM_018954344.2	0.122140438	chr7	42590405	42601246	-	108982873	1-phosphatidylinositol-3-phosphate 5-kinase FAB1B-like
XR_004801535.1	XM_035690204.1	0.12199927	chr5	19301466	19309978	+	109004040	uncharacterized LOC109004040
MSTRG.55875.1	XM_035690204.1	0.121817481	chr5	19301466	19309978	+	109004040	uncharacterized LOC109004040
MSTRG.109897.16	XM_018963267.2	0.121623272	chr7	15444524	15465521	-	108989601	beta-amyrin synthase-like
XR_001995902.1	XM_035690204.1	0.121600745	chr5	19301466	19309978	+	109004040	uncharacterized LOC109004040
MSTRG.55875.1	XM_035683649.1	0.121534173	chr12	3976006	3985355	+	108983218	cell division protein FtsY homolog, chloroplastic
XR_004800885.1	XM_035692586.1	0.121431386	chr7	15331891	15338212	+	109004270	beta-amyrin synthase
XR_001998063.2	XM_035683883.1	0.121405548	chr1	33416777	33424440	-	118344063	uncharacterized LOC118344063
XR_004802642.1	XM_018966810.2	0.121300335	chr13	30887602	30901317	+	108992290	uncharacterized LOC108992290
MSTRG.152528.3	XM_018974702.2	0.121145275	chr6	27221507	27232906	+	108998204	thaumatin-like protein 1b
XR_004801905.1	XM_035683883.1	0.121067196	chr1	33416777	33424440	-	118344063	uncharacterized LOC118344063
XR_001994779.2	XM_018959084.2	0.120997429	chr10	4905053	4907686	-	108986462	transcription factor TCP13-like
XR_001999980.2	XM_018959084.2	0.120982265	chr10	4905053	4907686	-	108986462	transcription factor TCP13-like
XR_004802642.1	XM_018976664.2	0.120900143	chr4	5535854	5538370	-	108999760	40S ribosomal protein S23
MSTRG.109897.16	XM_035689049.1	0.120896322	chr4	32172405	32176587	+	109004592	transcription factor bHLH130-like
MSTRG.109897.16	XM_018991068.2	0.120875832	chr6	10519971	10524297	+	109010290	cytochrome P450 CYP82D47-like
XR_004800885.1	XM_018978225.2	0.120753493	chr2	27317301	27318907	-	109001090	F-box/kelch-repeat protein SKIP25-like
XR_002001178.2	XM_035683883.1	0.120686704	chr1	33416777	33424440	-	118344063	uncharacterized LOC118344063
MSTRG.109897.16	XM_018975487.2	0.120628422	chr11	32369081	32371095	+	108998788	RING-H2 finger protein ATL13-like
XR_004801042.1	XM_018954630.2	0.12058326	chr9	16238379	16243103	-	108983095	long-chain-alcohol oxidase FAO4A-like
XR_001998020.2	XM_018966810.2	0.120556152	chr13	30887602	30901317	+	108992290	uncharacterized LOC108992290
XR_001998020.2	XM_018958924.2	0.120503943	chr7	50434181	50439306	+	108986346	serine/arginine-rich SC35-like splicing factor SCL30
XR_004798497.1	XM_018958924.2	0.120472002	chr7	50434181	50439306	+	108986346	serine/arginine-rich SC35-like splicing factor SCL30
XR_004801970.1	XM_035691098.1	0.120450658	chr6	3176830	3189137	+	109003422	receptor-like protein 14
MSTRG.55875.1	XM_035691872.1	0.120376426	chr7	48872965	48879686	+	108997043	pumilio homolog 4-like

MSTRG.109897.16	XM_018994923.2	0.120284688	chr14	8864325	8867157	-	109013013	scarecrow-like protein 34
MSTRG.109897.16	XM_035685276.1	0.120107307	chr14	28497998	28503114	+	109003650	YTH domain-containing protein ECT4
XR_004798497.1	XM_018974702.2	0.120093589	chr6	27221507	27232906	+	108998204	thaumatin-like protein 1b
MSTRG.109897.16	XM_018956526.2	0.120049757	chr5	3235359	3236088	-	108984537	protein EARLY RESPONSIVE TO DEHYDRATION 15-like
XR_004801671.1	XM_018959084.2	0.119920816	chr10	4905053	4907686	-	108986462	transcription factor TCP13-like
XR_004800885.1	XM_018972624.2	0.119867196	chr4	20534888	20540216	-	108996628	probable LRR receptor-like serine/threonine-protein kinase At3g47570
XR_004801700.1	XM_018991068.2	0.119789598	chr6	10519971	10524297	+	109010290	cytochrome P450 CYP82D47-like
MSTRG.109897.16	XM_018954630.2	0.119733853	chr9	16238379	16243103	-	108983095	long-chain-alcohol oxidase FAO4A-like
MSTRG.109897.16	XM_035688528.1	0.119647476	chr3	32014223	32042402	+	109004210	mRNA-capping enzyme-like
MSTRG.109897.16	XM_018980134.2	0.119461884	chr5	5598076	5607876	-	109002406	probable LRR receptor-like serine/threonine-protein kinase At1g06840
MSTRG.153333.1	XM_035692957.1	0.119394203	chr8	22286180	22291042	-	109017678	rust resistance kinase Lr10-like
XR_004801159.1	XM_018962761.2	0.119383526	chr14	8536278	8538423	-	108989223	probable L-type lectin-domain containing receptor kinase I.6
XR_004801671.1	XM_035683883.1	0.119286691	chr1	33416777	33424440	-	118344063	uncharacterized LOC118344063
MSTRG.55875.1	XM_018987330.2	0.119242997	chr16	7075619	7089046	+	109007582	uncharacterized LOC109007582
MSTRG.55875.1	XM_018982562.2	0.11922165	chr10	9156607	9159414	-	109004129	cytochrome P450 71AU50-like
XR_004802196.1	XM_035688887.1	0.11913048	chr3	28664621	28667043	+	118348056	MDIS1-interacting receptor like kinase 2-like
MSTRG.27387.1	XM_035690204.1	0.119110252	chr5	19301466	19309978	+	109004040	uncharacterized LOC109004040
XR_001994779.2	XM_018989558.2	0.118992514	chr7	8798957	8801574	-	109009172	GEM-like protein 7
XR_004802196.1	XM_035690805.1	0.11897609	chr6	36329659	36333134	+	118343755	disease resistance protein RUN1-like
XR_001994779.2	XM_035690805.1	0.118967261	chr6	36329659	36333134	+	118343755	disease resistance protein RUN1-like
XR_004800885.1	XM_018971107.2	0.11891024	chr6	12986090	12988819	-	108995530	receptor-like protein 7
MSTRG.142498.1	XM_018980132.2	0.118719686	chr5	5598076	5607897	-	109002406	probable LRR receptor-like serine/threonine-protein kinase At1g06840
MSTRG.55875.1	XM_018959084.2	0.118702725	chr10	4905053	4907686	-	108986462	transcription factor TCP13-like
XR_004801324.1	XM_035690204.1	0.118470744	chr5	19301466	19309978	+	109004040	uncharacterized LOC109004040
XR_004798497.1	XM_018958370.2	0.118464561	chr12	6587489	6596583	+	108985905	uncharacterized LOC108985905
XR_004801700.1	XM_018952208.2	0.118294442	chr4	20732565	20739290	-	108981125	probable LRR receptor-like serine/threonine-protein kinase At3g47570
MSTRG.109897.16	XM_018987325.2	0.117861846	chr16	7077657	7089046	+	109007582	uncharacterized LOC109007582
XR_004801671.1	XM_035690204.1	0.117838003	chr5	19301466	19309978	+	109004040	uncharacterized LOC109004040
XR_001998020.2	XM_018959084.2	0.11775443	chr10	4905053	4907686	-	108986462	transcription factor TCP13-like
XR_001998161.2	XM_018959084.2	0.117539487	chr10	4905053	4907686	-	108986462	transcription factor TCP13-like
XR_001996304.1	XM_018970957.2	0.1172894	chr7	4064116	4068193	+	108995385	phosphatidylinositol transfer protein 3
MSTRG.116494.1	XM_035689691.1	0.117240849	chr4	2229966	2235819	-	109000808	acetyl-coenzyme A carboxylase carboxyl transferase subunit alpha, chloroplastic-like
MSTRG.109897.16	XM_018966008.2	0.11722217	chr7	43763455	43766818	-	108991675	uncharacterized LOC108991675
MSTRG.152528.3	XM_035692957.1	0.11717771	chr8	22286180	22291042	-	109017678	rust resistance kinase Lr10-like
MSTRG.51561.8	XM_018987330.2	0.11708282	chr16	7075619	7089046	+	109007582	uncharacterized LOC109007582
XR_001998656.2	XM_018991132.2	0.116764516	chr16	23641881	23650296	+	109010332	uncharacterized LOC109010332
XR_004800885.1	XM_018984048.2	0.116759533	chr11	36419332	36421415	+	109005227	probable WRKY transcription factor 40
XR_001998020.2	XM_018953739.2	0.116688094	chr7	19029889	19031361	+	108982381	protein CANDIDATE G-PROTEIN COUPLED RECEPTOR 7-like
MSTRG.2510.1	XM_018958924.2	0.116683022	chr7	50434181	50439306	+	108986346	serine/arginine-rich SC35-like splicing factor SCL30
XR_001994995.2	XM_018996109.2	0.116672541	chr7	1023081	1024482	+	109013875	probable esterase D14L
XR_004798497.1	XM_018986703.2	0.116603321	chr7	39752153	39754226	-	109007132	1-aminocyclopropane-1-carboxylate oxidase homolog 1-like
XR_004801042.1	XM_018966810.2	0.116563886	chr13	30887602	30901317	+	108992290	uncharacterized LOC108992290
XR_001996202.2	XM_018967836.2	0.116416837	chr15	18297792	18301590	+	108993071	receptor-like protein kinase 7
XR_001995964.2	XM_035683883.1	0.116348363	chr1	33416777	33424440	-	118344063	uncharacterized LOC118344063
XR_004801159.1	XM_018991068.2	0.116328226	chr6	10519971	10524297	+	109010290	cytochrome P450 CYP82D47-like
XR_001994779.2	XM_035691194.1	0.116265287	chr6	36333082	36334787	+	108982793	disease resistance protein RPV1-like
MSTRG.109897.16	XM_019003429.2	0.116254953	chr7	27713554	27723424	+	109020905	clathrin interactor EPSIN 2
XR_001999201.2	XM_018959084.2	0.116180908	chr10	4905053	4907686	-	108986462	transcription factor TCP13-like
MSTRG.109897.16	XM_018986357.2	0.116160421	chr3	12490028	12494584	+	109006916	two-pore potassium channel 3-like
MSTRG.27387.1	XM_018994818.2	0.116146573	chr6	37006877	37008290	+	109012917	class V chitinase-like
XR_001994779.2	XM_035695754.1	0.116110423	chr11	36905446	36924095	-	108981918	autophagy-related protein 3

MSTRG.116494.1	XM_018951732.2	0.116058807	chr8	19850173	19851939	+	108980731	uncharacterized LOC108980731
MSTRG.102213.1	XM_018989717.2	0.115993077	chr6	17738434	17750081	+	109009277	guanine nucleotide-binding protein alpha-1 subunit
XR_004798497.1	XM_018951732.2	0.1159777	chr8	19850173	19851939	+	108980731	uncharacterized LOC108980731
XR_001994779.2	XM_018964840.2	0.115954169	chr7	47496743	47507393	+	108990767	MADS-box protein SOC1-like
XR_004802604.1	XM_018967922.2	0.115927657	chr15	18636140	18650000	-	108993142	lupeol synthase-like
XR_004801970.1	XM_018960036.2	0.115923822	chr2	13457576	13459485	+	108987166	transcription factor JUNGBRUNNEN 1-like
MSTRG.152528.3	XM_018994818.2	0.115866466	chr6	37006877	37008290	+	109012917	class V chitinase-like
MSTRG.27387.1	XM_035685276.1	0.115833676	chr14	28497998	28503114	+	109003650	YTH domain-containing protein ECT4
XR_004800885.1	XM_035683357.1	0.11581237	chr1	10128920	10130940	-	118343954	ankyrin repeat-containing protein At5g02620-like
MSTRG.2510.1	XM_018987325.2	0.115689862	chr16	7077657	7089046	+	109007582	uncharacterized LOC109007582
XR_001995902.1	XM_035683649.1	0.115610956	chr12	3976006	3985355	+	108983218	cell division protein FtsY homolog, chloroplastic
XR_001998063.2	XM_035691979.1	0.115544684	chr7	39113205	39115741	+	108991199	putative protein phosphatase 2C-like protein 44
XR_001996304.1	XM_018955260.1	0.115442639	chr7	5726972	5727550	+	108983577	receptor like protein 21-like
XR_001996304.1	XM_018979100.2	0.115347902	chr14	27324233	27326650	+	109001703	receptor-like protein 2
XR_004801970.1	XM_018981331.2	0.115335182	chr16	26910055	26915634	-	109003256	metacaspase-3-like
XR_004802599.1	XM_018962761.2	0.115327577	chr14	8536278	8538423	-	108989223	probable L-type lectin-domain containing receptor kinase I.6
XR_004802196.1	XM_018968359.2	0.115223969	chr13	6152510	6155107	+	108993436	probable amidase At4g34880
XR_004802642.1	XM_018966008.2	0.115166267	chr7	43763455	43766818	-	108991675	uncharacterized LOC108991675
XR_001998020.2	XM_035686287.1	0.115131367	chr16	1638414	1640140	-	118344841	spermine synthase-like
XR_001998020.2	XM_019003755.2	0.115117841	chr11	30073566	30078607	+	109021176	E3 ubiquitin-protein ligase UPL1-like
XR_001996083.2	XM_035694627.1	0.115001061	chr10	36915098	36919941	-	109004711	receptor-like protein 7
MSTRG.102213.1	XM_018965441.2	0.114985984	chr7	4972465	4974453	+	108991266	phenylcoumaran benzylic ether reductase TP7-like
MSTRG.116494.1	XM_035683649.1	0.114928373	chr12	3976006	3985355	+	108983218	cell division protein FtsY homolog, chloroplastic
MSTRG.144914.1	XM_035683649.1	0.114834238	chr12	3976006	3985355	+	108983218	cell division protein FtsY homolog, chloroplastic
MSTRG.152528.3	XM_035692760.1	0.11478932	chr1	18658866	18661714	+	109010137	uncharacterized LOC109010137
XR_001994779.2	XM_035690204.1	0.11477107	chr5	19301466	19309978	+	109004040	uncharacterized LOC109004040
MSTRG.109897.16	XM_018994818.2	0.114714356	chr6	37006877	37008290	+	109012917	class V chitinase-like
XR_004802642.1	XM_018975487.2	0.114712836	chr11	32369081	32371095	+	108998788	RING-H2 finger protein ATL13-like
XR_004802196.1	XM_018967836.2	0.114699944	chr15	18297792	18301590	+	108993071	receptor-like protein kinase 7
MSTRG.76070.1	XM_018968016.2	0.114640544	chr15	18451385	18461281	+	108993195	auxin response factor 19-like
MSTRG.142498.1	XM_018971421.2	0.114600691	chr11	15797680	15799985	-	108995791	putative F-box protein PP2-B12
XR_004801712.1	XM_035690204.1	0.114598472	chr5	19301466	19309978	+	109004040	uncharacterized LOC109004040
MSTRG.76070.1	XM_018963149.2	0.114587388	chr6	35275634	35278794	+	108989520	beta-amyrin 28-monooxygenase-like
MSTRG.76070.1	XM_035692586.1	0.114545559	chr7	15331891	15338212	+	109004270	beta-amyrin synthase
XR_001996179.2	XM_035683883.1	0.11454419	chr1	33416777	33424440	-	118344063	uncharacterized LOC118344063
XR_001999201.2	XM_019001945.2	0.114490413	chr16	1041342	1044257	-	109019612	plastid division protein PDV1-like
XR_004801970.1	XM_018973209.2	0.114269654	chr9	20318318	20319292	-	108997094	protein P21-like
XR_004801712.1	XM_018991068.2	0.114253926	chr6	10519971	10524297	+	109010290	cytochrome P450 CYP82D47-like
MSTRG.153333.1	XM_018953805.2	0.114245691	chr7	20190928	20193977	-	108982432	receptor-like protein 7
XR_001994779.2	XM_018973918.2	0.114208656	chr12	2816907	2822586	+	108997575	DNA-directed RNA polymerases II and IV subunit 5A-like
MSTRG.76070.1	XM_019003281.2	0.114170453	chr9	20791268	20792982	-	109020760	AT-hook motif nuclear-localized protein 24-like
MSTRG.27387.1	XM_018959084.2	0.113989625	chr10	4905053	4907686	-	108986462	transcription factor TCP13-like
MSTRG.122249.3	XM_018987330.2	0.113950655	chr16	7075619	7089046	+	109007582	uncharacterized LOC109007582
XR_001998656.2	XM_018991068.2	0.113933982	chr6	10519971	10524297	+	109010290	cytochrome P450 CYP82D47-like
XR_001998161.2	XM_018951732.2	0.113891256	chr8	19850173	19851939	+	108980731	uncharacterized LOC108980731
MSTRG.97720.1	XM_035690204.1	0.113889571	chr5	19301466	19309978	+	109004040	uncharacterized LOC109004040
XR_004800885.1	XM_018975178.2	0.113839812	chr1	9012294	9015820	+	108998594	serine/arginine-rich splicing factor RS40-like
XR_001999980.2	XM_018986529.2	0.113771838	chr13	3859017	3862496	-	109007037	transmembrane emp24 domain-containing protein p24delta3-like

Table S5. Primers used for qRT-PCR			
**Gene**	**Forward primer (5′-3′)**	**Reverse primer (5′-3′)**	**Tm**
lncRNA109897	CCCAAGCATATCTCAAACAA	CAAGGAGTGAAGTGATGATAC	F-51.3/R-53.7
JrCCR4	CCATCTATTCGGACAACAG	CTTCAGAGCACAACTACG	F-49.9/R-50.1
JrTLP1b	TTACGATGTCAGCCTTGT	AGCAGTATTGTGGTTGAGT	F-50.3/R-50.9
AT3G18780-Actin2-realtime	GACCTTGCTGGACGTGACCTTAC	GTAGTCAACAGCAACAAAGGAGAGC	F-60.5/R-58.2
18S rRNA	GGTCAATCTTCTCGTTCCCTT	TCGCATTTCGCTACGTTCTT	F-55.6/R-53.4

Table S6. Primers used for the isolation and plasmids construction of the CDS and promoter sequences.		
Gene (plasmids)	Forward primer (5-3′)	Reverse primer (5-3′)
lncRNA109897(PRI101)	CCATATGATGAGTTCAGTTTATGTCACTTCTAGTATATTT	GGAATTCGTAAATTTGAAGCCTATACACAAATTGG
lncRNA109897(PYL156)	GGAATTCATGGCTACTGGTAGTGTATCATCACTT	GGGGTACCGTGACCTACTGTATTGATTTCCATAGG
JrCCR4(PRI101)	CGTCGACATGGCTTTCATCTTTAACAACT	CGGATCCCGTAGAAGACCCAGTTGTAGAT
JrCCR4(PYL156)	GGAATTCATGGAAGAATTGTTTGCTGGG	GGGGTACCCAGCCTTTACCTTTGCAATATCTG
TLP1b(PRI101)	ttgatacatatgcccgtcgacATGATTTTGAAGGGGCCAAGT	tcagaattcggtacccccgggAGGGCAAAAAGTGATAACGTAATTAG
TLP1b(PYL156)	agaaggcctccatggggatccGGACAGGTTGCATGCAATGG	gagacgcgtgagctcggtaccTTGAGGGCACTGGTTCTCAAA
TRV1	TTACAGGTTATTTGGGCTAG	CCGGGTTCAATTCCTTATC
TRV2	ACATTGTTACTCAAGGAAGCACG	AAGATCAGTCGAGAATGTCAATCTC
TLP1b(pGADT7)	gtaccagattacgctcatatgATGATTTTGAAGGGGCCAAGT	cagctcgagctcgatggatccTCAAGGGCAAAAAGTGATAACGT
JrCCR4(pGBKT7)	tcagaggaggacctgcatatgATGGCTTTCATCTTTAACAACTCCA	ccgctgcaggtcgacggatccCGTAGAAGACCCAGTTGTAGATCG
JrCCR4F1(pGBKT7)	tcagaggaggacctgcatatgATGGTATCGATTTCTGAGACTTCTGA	ccgctgcaggtcgacggatccCATCTTGTCATCCCAACCAGTG
JrCCR4F2(pGBKT7)	tcagaggaggacctgcatatgATGAGATATTGCAAAGGTAAAGGCT	ccgctgcaggtcgacggatccCGTAGAAGACCCAGTTGTAGATCG
JrCCR4(pSPYCE)	tggcgcgccactagtggatccATGGCTTTCATCTTTAACAACTCCA	agcggtaccctcgaggtcgacCGTAGAAGACCCAGTTGTAGATCG
TLP1b(pSPYNE)	CGGATCCATGATTTTGAAGGGGCCAA	GCGTCGACAGGGCAAAAAGTGATAACGTAATTAG
TLP1b (pET32a)	gccatggctgatatcggatccATGATTTTGAAGGGGCCAAGT	ttgtcgacggagctcgaattcAGGGCAAAAAGTGATAACGTAATTAG
JrCCR4(pGEX-4T-1)	gatctggttccgcgtggatccATGGCTTTCATCTTTAACAACTCCA	ctcgagtcgacccgggaattcCGTAGAAGACCCAGTTGTAGATCG
JrCCR4(PHB)	accagtctctctcaagcttATGGCTTTCATCTTTAACAACTCCA	gcccttgctcaccatggatccCGTAGAAGACCCAGTTGTAGATCG
lncRNA109897(pGreenII-62-SK)	cgctctagaactagtggatccATGAGTTCAGTTTATGTCACTTCTAGTATATTT	gtcgacggtatcgataagcttGTAAATTTGAAGCCTATACACAAATTGG
proJrCCR4 (pGreenII-0800-Luc)	gggccccccctcgaggtcgacGATTCAGGAATAATTTTGCATATGAAA	cgctctagaactagtggatccGGAAATAATGGGCAAGTTTTGATA
JrCCR4(pGreenII-62-SK)	cgctctagaactagtggatccATGGCTTTCATCTTTAACAACTCCA	gtcgacggtatcgataagcttCGTAGAAGACCCAGTTGTAGATCG
proTLP1b (pGreenII-0800-Luc)	gggccccccctcgaggtcgacCATAACTTTCAAGTAATGTTACTACTTCAGAA	cgctctagaactagtggatccAATCCTGACTTCATCCGGACTG

TLP1b (pGreenII-62-SK)	cgctctagaactagtggatccATGATTTTGAAGGGGCCAAGT	gtcgacggtatcgataagcttAGGGCAAAAAGTGATAACGTAATTAG
prolncRNA109897 (pGreenII-0800-Luc)	gggccccccctcgaggtcgacTTGTGTATCCTGGTAGTGATATCTTAAGTC	cgctctagaactagtggatccGTTGTAATGATGAGTTGAGATGAGTTGA
